# Molecular lead halide perovskite layer bridged AgBiS_2_ nanocrystals for efficient thin film solar cells

**DOI:** 10.1038/s41467-026-72272-4

**Published:** 2026-04-24

**Authors:** Wanpeng Yang, Tianyu Sun, Haixuan Yu, Haodan Shi, Yong Hu, Junyi Huang, Zhirong Liu, Ying Xu, Lei Wang, Bing Hu, Yan Shen, Mohammad Khaja Nazeeruddin, Mingkui Wang

**Affiliations:** 1 Wuhan National Laboratory for Optoelectronics, School of Optoelectronic Science and Engineering, Huazhong University of Science and Technology, Wuhan, Hubei P. R. China; 2 Wuhan Hero Optoelectronics Technology Co., LTD, 6 Huanglongshan North Road, East Lake High-Tech Development Zone, Wuhan, Hubei P.R. China; 3 Institut des Sciences et Ingénierie Chimiques, Ecole Polytechnique Fédérale de Lausanne, Lausanne, Switzerland; 4 Mechanical and Energy Engineering Department, College of Engineering, Imam Abdulrahman Bin Faisal University, Dammam, Saudi Arabia

**Keywords:** Nanoparticles, Electronic properties and materials

## Abstract

Ternary chalcogenide AgBiS_2_ nanocrystals have emerged as an environmentally friendly and stable material for ultra-thin film lightweight low-cost solar cells. However, their development is currently limited by the poor charge transport characteristics, mainly due to low carrier mobility and the prevalence of surface defects. This leads to a short carrier diffusion length, which severely restricts the thickness of the photoactive layer and the absorption of near-infrared photons. Here, we demonstrate ligand-mediated heteroepitaxial growth of a molecular lead halide perovskite layer bridges along the (100) facet of AgBiS_2_ nanocrystals, facilitating both efficient surface passivation and charge transport. The bridged nanocrystals enable the annealing process at elevated temperatures without inducing defect formation. This results in a greater cationic disorder, fully activating their light-absorption capability. The synergistic effect of structural modulation and cation disorder engineering addresses the long-standing trade-off between charge extraction and light absorption of AgBiS_2_ nanocrystal solar cells, enabling thick-film fabrication to compensate for losses in infrared absorption. Consequently, the resultant solar cells with a 185 nm-thick AgBiS_2_ nanocrystal layer achieve a certified power conversion efficiency of 11.22% and a short-circuit current of ~ 34 mA cm^-2^ under AM 1.5 G illumination (aperture area: 0.022 cm^2^), representing a record-high performance.

## Abstract (web summary)

AgBiS₂ nanocrystals show promise for thin, low-cost solar cells but are hindered by poor charge transport and limited infrared absorption. Yang et al. grow a molecular lead halide perovskite layer on the nanocrystals to passivate defects, improve transport, and enable highpower conversion efficiency.

## Introduction

AgBiS_2_ nanocrystals (NCs) have attracted considerable interest for high efficiency and stable photovoltaics due to their cost-effective, high absorption (>10^5 ^cm^−1^) and low-temperature solution processability^1–3^. Recent advances in surface chemistry^4,5^, bandgap engineering^6,7^, and optimization of the device architecture^8,9^, in particular the use of 3-chloro-1-propanethiol (CPT) ligands for defect passivation, have increased the power conversion efficiency (PCE) of AgBiS_2_ NC solar cells to 10.86% (9.4% certified)^10^. Charge transport in AgBiS_2_ NCs is still limited by carrier mobility (~10^-5 ^cm^2 ^V^-1^ s^-1^), surface trap-assisted recombination and disordered hopping pathways^1,5,11^, resulting in an ultrashort diffusion length (~25 nm)^1,12,13^. Therefore, as demonstrated by recent studies of AgBiS_2_ NC photovoltaic systems, the thickness of the photoactive layer should be kept below 40 nm in order to minimize the loss of bulk recombination^1,10,14,15^. Though this ultrathin film is sufficient to absorb most of the light in the visible range, significant losses of photons in the infrared range (reflection and transmission losses of up to 10.5 mA cm⁻²) were observed due to the insufficient optical path extension^1^. To address this limitation, Kim et al. implemented a V-groove-textured film in the device to enhance light trapping^7^. A remarkable photocurrent exceeding 31 mA cm⁻² was achieved using bulk AgBiS_2_ films over 200 nm thick^16^, benefiting from the superior charge-transport performance of the bulk material. However, thick AgBiS_2_ NC film (>180 nm) suffers from ultra-short diffusion length and exhibits a saturating current of ~16 mA cm⁻²^1,13,17^. Efforts to resolve this dilemma have therefore focused on two frontiers: (1) enhancing carrier mobility via device architecture innovations^7–9^, and (2) suppressing surface defects through refined synthesis processes^18–22^ or effective surface passivation techniques^5,10^.

AgBiS_2_ NCs exhibit the same crystal structure as CsPbBr_3_ perovskites, namely cubic rock-salt (space group: *Fm-3m*), with a noteworthy lattice mismatch of merely ~2.8% (5.69 Å *vs*. 5.85 Å)^23–25^. This structural compatibility opens up unprecedented opportunities for epitaxial heterointerface engineering in AgBiS_2_^26^. Here, we propose a ligand-mediated heteroepitaxial approach for bridging AgBiS_2_ NCs, where small amounts of CsPbX_3_ (X = I, Br) precursors are introduced during the SPLE to serve as surface-passivation ligands and epitaxial templates. The post-annealing and washing process triggers in situ growth of a molecular lead halide perovskite layer on the AgBiS_2_ (100) facet, effectively passivating the (100) facets and bridging the adjacent NCs to form bridged NCs with nearly defect-free, atomically coherent interfaces^27–29^. Leveraging the high thermal stability of inorganic perovskite bridges between NCs^30^, the prepared NC films can undergo high-temperature annealing at 150°C to achieve greater cation disorder, thereby fully activating their light-absorption capabilities. The synergistic effect of structural modulation and cation disorder engineering resolves the long-standing trade-off between charge extraction and light absorption in AgBiS_2_ NC solar cells, enabling the fabrication of thick NC films to compensate for infrared absorption losses. Ultimately, AgBiS_2_ NC solar cells with a 185 nm-thick photoactive layer achieved a certified PCE of 11.22%. This work presents a comprehensive framework for modulating AgBiS_2_ NC, paving the way for eco-friendly optoelectronics.

## Results

**Fig. 1:**
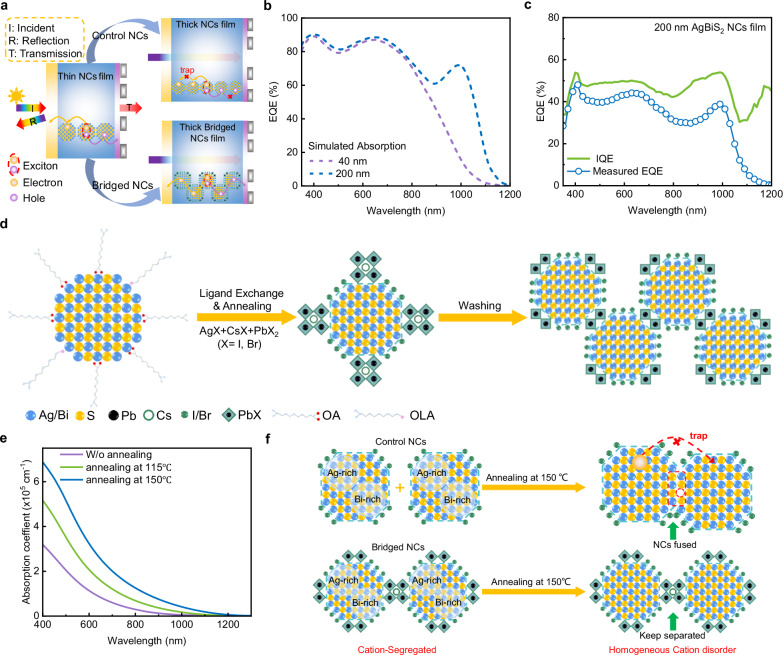
Light absorption loss and charge transport in ultrathin and thick nanocrystal (NC) films. **a** A schematic diagram of light loss through transmission and reflection in ultrathin films, as well as light absorption and charge transport of the thick films prepared from control NCs and bridged NCs. **b** The simulated external quantum efficiency (EQE) of AgBiS_2_ NC solar cell using the transfer matrix method with the photoactive layer thicknesses of 40 nm and 200 nm, respectively. **c** The measured EQE and calculated the internal quantum efficiency (IQE) of AgBiS_2_ NC solar cell with a 200 nm-thick photoactive layer. **d** A schematic diagram of the fabrication of CsPbX_3_ perovskite modified AgBiS_2_ NCs ink, and the AgBiS_2_ NCs bridged by a molecular perovskite layer. **e** Absorption of bridged NC films annealed at different temperatures. **f** A schematic representation of the homogeneous cation disorder obtained in control NCs and bridged NCs annealed at 150°C. Control NCs fuse along the (100) facet, generating vacancies and other defects at the boundaries, while bridged NCs remain independent from one another.

The adoption of ultrathin AgBiS_2_ NC films has been demonstrated to facilitate efficient charge transport by reducing the carrier’s diffusion distance. However, it has been observed that the films fail to fully exploit the incident photons via multiple reflection or optical path extension, particularly in the infrared spectral region where photon penetration is significant (Fig. 1a, left). We first performed a transfer matrix method simulation to evaluate the light-harvesting potential of a thick AgBiS_2_ NC absorber layer (Supplementary Fig. 1), in which the internal quantum efficiency (IQE) was assumed to be 100%. As shown in Fig. 1b, the device with a 200 nm-thick AgBiS_2_ NC layer exhibited a significantly enhanced infrared response (760–1200 nm) in comparison to its 40 nm counterpart, with a simulated maximum current density (J_SC_) of 32.6 mA cm⁻² in contrast to 27.5 mA cm⁻² for the device utilizing 40 nm-thick absorber. We fabricated a device with a 200 nm-thick AgBiS_2_ NCs film. However, the integrated current from the external quantum efficiency (EQE) measurement was found to be only 16.8 mA cm⁻² (Fig. 1c). Further analysis of the IQE (IQE = EQE/absorbance) shows that the IQE is less than 50% at 400–1200 nm, thus confirming the poor charge transport characteristics observed in thick AgBiS_2_ NC films (Fig. 1a, right). In order to address this issue, we introduced CsPbX_3_ (X = I^⁻^, Br^⁻^) perovskite components into a conventional ligand solution (AgX_2_⁻) to enable heteroepitaxial growth on the (100) facets of AgBiS_2_ NCs following annealing (Fig. 1d). However, an excessively thick perovskite matrix forms at this stage, which impedes charge transport and compromises device performance (Supplementary Fig. 2). It is imperative that the washing process is performed with acetonitrile (ACN) in order to remove excess perovskite matrix in NC films, thereby thinning the matrix to molecular-scale configurations (Supplementary Fig. 3). The objective of this approach is to achieve efficient passivation on nonpolar (100) facets of AgBiS_2_ NCs and to construct a reinforced charge transport network by bridging NCs with the molecular perovskite layer, thereby enabling the fabrication of thick absorbers with excellent charge transport and absorption in the infrared region (Fig. 1a, right). The high thermal stability of inorganic perovskite bridges between NCs is beneficial for this process, and the prepared NC films can be annealed above 150 °C to achieve greater cation disorder, thereby fully activating their light-absorption capability while avoiding defect formation triggered by NC fusing (Fig. 1e, f). Supplementary XRD patterns of films annealed at varying temperatures provide further evidence for the temperature-induced cation disordering process (Supplementary Fig. 4). The synthesis method documented in the extant literature^31^ was adapted for the purpose of producing uniform and monodisperse NCs, with a view to ensuring that the connections between the individual NCs are precisely aligned (Supplementary Fig. 5).

The two ligand ensembles (control: AgX and MAX; perovskite modified (denoted as P-modified): AgX, CsX, and PbX_2_) are soluble in N, N-dimethylformamide (DMF) (Supplementary Fig. 6a, b). Both the control and P-modified NC inks effectively process phase transfer from non-polar solvent (octane) to polar solvent (DMF) following the SPLE treatment, in which the P-modified NCs exhibit a substantial enhancement in ligand exchange efficiency. Fourier transform infrared (FTIR) spectroscopy further confirms complete desorption of oleic acid (OA)/oleylamine (OLA) ligands in the P-modified NCs (Supplementary Fig. 6c). In contradistinction to the conventional alkali metal effect, the enhanced phase transfer capability can be attributed to the increased adsorption of lead halide ligands on the surface of NCs in comparison to AgX_2_^-^ ligands (Supplementary Figs. 6d–f and 7, Supplementary Table 1)^5,32,33^.

Subsequent to a period of five hours of static storage at room temperature, the P-modified NCs ink exhibited a homogeneous black coloration, while the control NCs ink showed evident signs of aggregation and browning (Supplementary Fig. 8a). The enhanced colloidal stability can be attributed to the strong adsorption of lead halide ligands on the surfaces of AgBiS_2_ NCs, which significantly increases the ligand coverage density. This finding is corroborated by the halide-to-Ag ratio, as determined by X-ray photoelectron spectroscopy (XPS) (Supplementary Figs. 8b and 9, Supplementary Table 2). A lower zeta potential of −42 mV, along with the reduced time-dependent absorption decay in the P-modified NCs ink monitored at 900 nm, provides further confirmation of the superior stability and suppression of aggregation due to the excellent passivation effect of the P-modified ligands (Supplementary Fig. 8c–e). It is noteworthy that the freshly prepared inks of the control and P-modified NCs showed almost identical UV-Vis spectra, with no absorption features related to perovskite observed in the latter (Supplementary Fig. 8 f)^34–36^. Furthermore, the perovskite-modified NCs film before ACN washing exhibits a slight enhancement in light absorption at wavelengths below 550 nm, likely originating from minimal absorption by the trace CsPbX_3_ perovskite. The absorption became virtually identical to the control sample after washing with ACN.

**Fig. 2:**
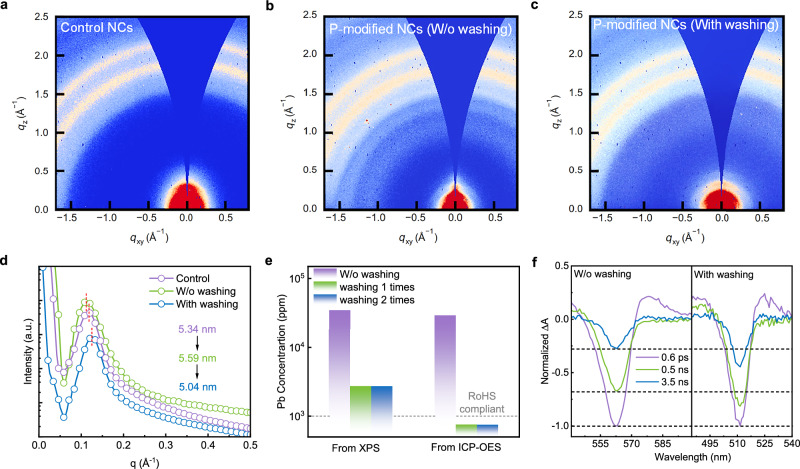
Molecular lead halide perovskite matrix formed via perovskite modified NCs ink after the washing process with ACN. Grazing-incidence wide-angle X-ray scattering (GIWAXS) measurements in 2D pattern of the control NC films (**a**), P-modified NC films without washing (**b**) and with washing (**c**). **d** The horizontal linecut GISAXS profiles of the corresponding NC films. **e** The Pb content in P-modified NC films before and after ACN washing, as determined by X-ray photoelectron spectroscopy (XPS) and inductively coupled plasma optical emission spectrometry (ICP-OES). **f** The comparison of normalized transient absorption spectra of P-modified NCs before and after ACN washing measured at 0.6 ps, 0.5 ns, and 3.5 ns time delays.

Grazing-incidence wide-angle X-ray scattering (GIWAXS) technique was utilized to monitor the perovskite formation within P-modified NC films with and without ACN washing (Fig. 2a–c). Despite the generally poor crystallinity of NCs and the limitations of the light source, which resulted in low-intensity diffraction rings exhibiting broad distribution, two distinct rings at ~19 nm⁻¹ and ~22 nm⁻¹ corresponding to the (111) and (200) planes of AgBiS_2_ were resolvable in all three NC films (Supplementary Fig. 10a), respectively^21^. The P-modified NC films clearly exhibited perovskite signals in the absence of washing, thereby substantiating the formation of cubic CsPbX_3_. After washing, the perovskite signals are almost completely absent. This phenomenon was also observed in the X-ray diffraction (XRD) patterns (Supplementary Fig. 10b), thus confirming the effective removal of excess perovskite matrix. The absence of a distinct signal of perovskite is consistent with the findings of previous studies of ultrathin molecular perovskite shell coatings. In these studies, surface modification at sub-nanometer resolution was found to minimally perturb bulk crystallinity^34–38^. It is noteworthy that the diffraction signals of P-modified NCs significantly manifest a considerable shift towards lower *q*-values or lower angles (Supplementary Fig. 10a, b). This phenomenon is attributed to the tensile strain induced by hetero-epitaxial growth of perovskite on the surface of NCs (Supplementary Fig. 10c). The P-modified NC films without a washing process exhibited a thicker perovskite matrix compared to those that underwent a washing process. This observation is indicative of higher tensile strain ^37,38^.

Grazing-incidence small-angle X-ray scattering (GISAXS) measurements were performed in order to track the changes in packing density of P-modified NC films with and without washing (Fig. 2d and Supplementary Fig. 10d–f). The P-modified films without a washing process exhibited increased inter-dot distance (5.59 nm compared to 5.34 nm in the control group), and this excessively thick matrix seriously hinders charge transport^37^. By contrast, the P-modified NC films that were washed achieved tighter packing (5.04 nm), effectively facilitating inter-NC carrier transport. The thickness of the perovskite matrix between the NCs (after washing) was estimated to be about 0.33 nm. This value was obtained by subtracting the diameter of the NC from the measured inter-dot distance of near-monodisperse spherical NCs. This value closely matches the dimensions of the perovskite monolayer, i.e., about half of its lattice constant.

XPS was employed to monitor changes in the signals of major elements in P-modified NC films following the washing process (Supplementary Fig. 9 and Supplementary Table 2). The results clearly demonstrate that the signal intensities of Cs 3 *d*, Pb 4 *f* and I/Br 3 *d* decreased after washing, particularly for Pb, thereby confirming the effective removal of excess perovskite matrix in the NCs film by ACN. Furthermore, semi-quantitative XPS analysis indicated a drastic decrease in Pb content, from 34,830 ppm to 2752 ppm after washing, which is below the detection limit of the XPS instrument (~3000 ppm). A more precise evaluation by inductively coupled plasma optical emission spectrometry (ICP-OES) analysis demonstrates that the Pb content is as low as 754 ppm after washing, thus surpassing RoHS compliance (<1000 ppm) and validating the eco-friendly claims made for the synthesized AgBiS_2_ system (Fig. 2e, Supplementary Table 3). A comparison was also made of the Pb content before and after the second ACN washing, and it was found that the value remained almost unchanged. It is clear that this phenomenon is attributable to the lattice anchoring effect between the AgBiS_2_ surface and the molecular perovskite layer (Supplementary Fig. 11). The perovskite matrix, which is hypothesized to exist as an independent entity, has been effectively dissolved during the initial washing process. However, the lattice anchoring effect between the molecular perovskite layer and the adjacent AgBiS_2_ surfaces stabilizes the perovskite and prevents its dissolution.

A series of transient absorption spectroscopy (TAS) measurements was conducted with the objective of detecting the presence of a molecular perovskite layer in the P-modified NC films following a washing process. As shown in Fig. 2f and Supplementary Fig. 12, the P-modified NC film, prior to being washed, displayed a characteristic ground state bleach (GSB) signal of CsPbX_3_ at a wavelength of 545–585 nm. The broadband GSB signal, centered at 800–1200 nm, is ascribed to AgBiS_2_^15^. Moreover, obvious carrier transfer processes were observed, thus confirming the formation of a type-I heterojunction structure between CsPbX_3_ perovskite and AgBiS_2_ NCs in the P-modified NC films without washing (Supplementary Note 1 for further details). Following the washing process, a weak perovskite GSB signal peak was observed in the range of 495-525 nm. In comparison with the perovskite signal in the P-modified NC film without a washing process, a substantial blue shift is observed. This phenomenon is attributed to exciton confinement, indicating that the perovskite exists in a highly confined state rather than a bulk phase ^39^.

**Fig. 3:**
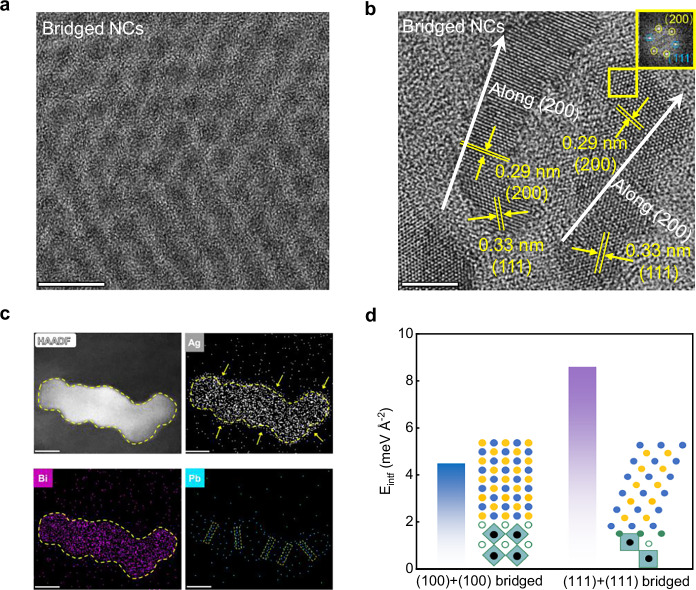
Molecular CsPbX_3_ layer bridge AgBiS_2_ NCs along the (200) facet. **a** High-resolution transmission electron microscopy (HR-TEM) image of the P-modified NCs after the washing process with ACN. Scale bar: 20 nm. **b** HRTEM images of the bridged NCs connected along the (200) facet. Scale bars: 5 nm. The inset shows the selected-area fast Fourier transformed (FFT) pattern. **c** High-resolution high-angle annular dark-field scanning TEM (HAADF-STEM) image and energy-dispersive X-ray spectroscopy (EDS) mapping of P-modified NCs after ACN washing. Scale bars: 5 nm. The dwell time for the Pb signal was increased to improve characterization of its spatial distribution at interfaces. **d** The interface energies of the (100) and (111) interface models. The inset (left) shows the interface model of CsPbBr_3_ (100) facet terminated by PbBr_2_ and AgBiS_2_ (100) facet. The inset (right) shows the interface model of CsPbBr_3_ (111) facet with Pb termination and AgBiS_2_ (111) facet passivated by Br atoms.

The epitaxial growth of the molecular perovskite layer on the surface of P-modified AgBiS_2_ NCs with washing was further verified with high-resolution transmission electron microscopy (HR-TEM). The HR-TEM images show that the NCs are effectively bridged to one another (Fig. 3a and Supplementary Fig. 13a), while the control NCs are significantly fused and aggregated (Supplementary Fig. 13b, c). The presence of such dimers has been shown to introduce numerous trap states at the interface, with the consequence that this affects the charge transport^5^. A partial magnified HR-TEM and fast Fourier transform analysis demonstrate that the NCs are connected along (200) facets, corresponding to a measured interplanar distance of 2.9 Å (Fig. 3b)^6^. The lattice spacing at the interface of two NCs is in good agreement with the values within an individual AgBiS_2_ NC, indicating the presence of a strict defect-free heteroepitaxial alignment at the (100) facet^37^. The high-angle annular dark-field scanning TEM image and energy-dispersive X-ray spectroscopy (EDS) elemental mapping in Fig. 3d reveal the uniform distributions of Ag and Bi throughout the bridged NCs. The Ag signal detected surrounding the bridged NCs (outside the yellow-framed area) originates from the adsorption of AgX_2_⁻ ligands on the NC surface. Concurrently, the trace Pb signal exhibited a pronounced concentration, manifesting specifically at the bridging regions between individual NCs (Fig. 3c and Supplementary Fig. 13a, d). This interfacial Pb signal accumulation is indicative of a configuration formed via NC bridging rather than NC fusion, as interfacial ligand depletion in fused structures would diminish Pb signal (Supplementary Fig. 13e, f). In summary, the measured thickness of the perovskite matrix between the NCs (after a thorough washing process), the blue shift of the GSB signal, and the TEM images confirm that a molecular perovskite layer bridges the NCs via epitaxial growth along the (100) facet. In the case of P-modified NCs without a washing process, TEM images revealed that perovskite also grows epitaxially on the surface of the NCs. However, the excessively thick perovskite matrix is unable to effectively bridge the NCs, thereby hindering charge transport (Supplementary Fig. 14)^40^. Furthermore, lattice matching is a prerequisite for bridging NCs (Supplementary Fig. 13 g).

The thermodynamic properties of the interface between AgBiS_2_ and perovskite were examined using density functional theory (DFT) calculations to facilitate understanding of the atomic-level mechanism of perovskite-bridged NCs. As illustrated in Supplementary Fig. 15, both AgBiS_2_ and perovskite are crystallized in a cubic crystal system, exhibiting comparable lattice constants and thus favoring epitaxial growth. As shown in Fig. 3d, this study has examined two probable (100) and (111) interfaces of AgBiS_2_ NCs for the purpose of epitaxial growth of perovskite^6,12^. The markedly reduced interfacial energy (E_intf_) of the (100) epitaxial interface (4.5 meV Å⁻² vs. 8.6 meV Å⁻²) substantiates its primacy in epitaxial growth, a finding that is in alignment with the experimental observations of the NCs being bridged along the (100) facet.

**Fig. 4:**
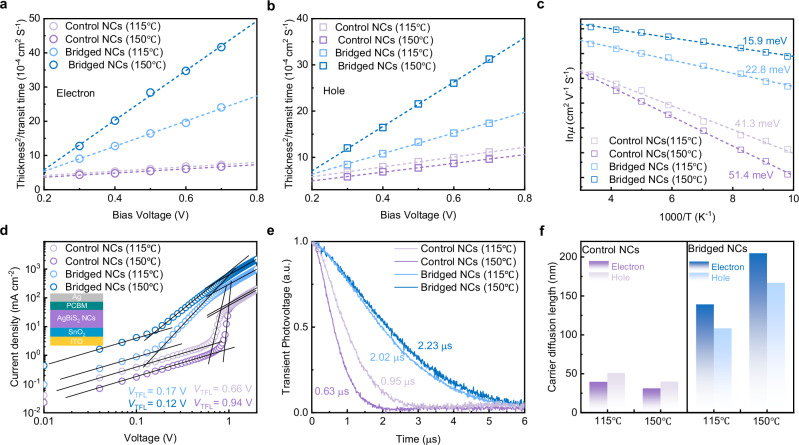
Improved charge transport and passivation by molecular lead halide perovskite bridges. Carrier mobility of control NC and bridged NC films under 115 °C and 150 °C annealing, derived from time-of-flight (TOF) measurements: **a** electron mobility and **b** hole mobility. **c** Carrier migration activation energy (*E*_a_) of control NC and bridged NC films under 115 °C and 150 °C annealing fitted by the Arrhenius equation. **d** The space charge limited conduction (SCLC) curves of electron-only devices fabricated by the control NC and bridged NC films under 115 °C and 150 °C annealing, respectively (from 0 to 4.0 V). The inset shows the schematic stack structure of an electron-only device. **e** Transient photovoltage measurements of devices fabricated by the control NC and bridged NC films under 115 °C and 150 °C annealing, respectively. **f** Carrier (electron and hole) diffusion length of control NC and bridged NC films under 115 °C and 150 °C annealing.

The resistivity of control NC and bridged NC films was measured by means of the four-probe method. The bridged NC film exhibited a reduced resistivity (94 Ω·cm) in comparison to the control (1835 Ω·cm). The carrier mobility was estimated using time-of-flight (TOF) measurements (Supplementary Fig. 16, Supplementary Tables 4–5). As shown in Fig. 4a-b, at the conventionally optimized annealing temperature of 115 °C, the electron mobility (μ_e_) of bridged NC films exhibited a 500% increase (from 6.36 × 10^-4^ to 3.67 × 10^-3 ^cm^2 ^V^-1^ s^-1^) and the hole mobility (μ_h_) a 100% increase (from 1.06 × 10^-3^ to 2.23 × 10^-3 ^cm^2 ^V^-1^ s^-1^) in comparison to the control NC films. This enhancement is attributed to the reduced inter-NC spacing in bridged NCs, thereby strengthening charge coupling. This result is higher than the mobilities measured in the contemporary devices (where μ_e_ = 8.13 × 10^-4 ^cm^2 ^V^-1^ s^-1^, μ_h_ = 1.1 × 10^-3 ^cm^2 ^V^-1^ s^-1^)^10^. Subsequently, an attempt was made to raise the annealing temperature to 150 °C, which resulted in a further doubling of the electron mobility of bridged NC films (from 3.67 × 10^-3^ to 7.24 × 10^-3 ^cm^2 ^V^-1^ s^-1^). This phenomenon can be attributed to the more delocalized electronic states present in NCs, which arise from greater cationic disorder at elevated temperatures^11^. In contrast, the mobility of control NC films exhibited a slight decrease at 150 °C. This is due to the enhanced delocalization of electronic states that are achieved at elevated temperatures. However, these states are unable to compensate for carrier trapping by defect states that are generated from NC fusing during 150 °C annealing (Supplementary Fig. 17). A systematic investigation was conducted into the carrier mobility of both NC films under varying annealing temperatures. The carrier mobility of the bridged NC films reached the maximum value at 150 °C, whereas that of the control NC films peaked at approximately 115–130°C (Supplementary Fig. 18a, with a detailed analysis provided in Supplementary Note 2). Temperature-dependent mobility analysis (Supplementary Fig. 18b) reveals thermally activated “hopping” transport in both bridged NC films and control NC films^41,42^, with carrier mobility decreasing as temperature declines. As shown in Fig. 4c, the markedly diminished activation energy (E_a_) derived from Arrhenius fitting in bridged NCs elucidates their enhanced mobility, which is attributed to the potentiated inter-NC charge coupling via atomically continuous molecular perovskite bridges and reduced carrier trapping at (100) interfaces (Supplementary Fig. 18c).

Electron-only devices (the inset in Fig. 4d) were fabricated in order to estimate the trap density (*N*_trap_) and *μ*_e_ of control NC and bridged NC films in the space-charge-limited current (SCLC) regions (Fig. 4d). The results indicate a significant decrease in trap density (from 6.95 ×  10^17^ to 1.76 × 10^17^cm^-3^) and an augmentation in electron mobility by an order of magnitude (from 5.42  × 10^-4^ to 3.25 × 10^-3 ^cm^2 ^V^-1^ s^-1^) in bridged NC films undergoing annealing at 115 °C (Supplementary Table 6 for further details). Increasing the annealing temperature to 150 °C further reduces defect-state density in bridged NC films, likely due to enhanced crystallinity, while substantially improving carrier mobility, consistent with TOF measurements. In contrast, the control NC films exhibited a marked increase in defect density. Analogous trends were observed in hole-only devices (Supplementary Fig. 19 and Supplementary Table 7). Furthermore, analysis of O 1 *s* XPS signals further confirmed effective (100)-facet passivation of bridged NCs via the epitaxial growth of perovskite (Supplementary Fig. 20). Transient photocurrent/photovoltage (TPC/TPV), in addition to the light intensity-dependent open-circuit voltage (V_OC_) and J_SC_, serve to confirm the superior charge transport and suppressed charge recombination in the devices initially fabricated with bridged NCs (Fig. 4e, Supplementary Figs. 21–22, with a detailed analysis provided in Supplementary Note 3). Correlating TPV-derived carrier lifetimes with TOF-measured mobility enables the valuation of carrier diffusion lengths. This approach has been shown to result in a substantial enhancement in bridged NC films (for electron change from 39.5 to 204.3 nm; for hole change from 50.9 to 166.5 nm, Fig. 4f and Supplementary Table 8).

**Fig. 5:**
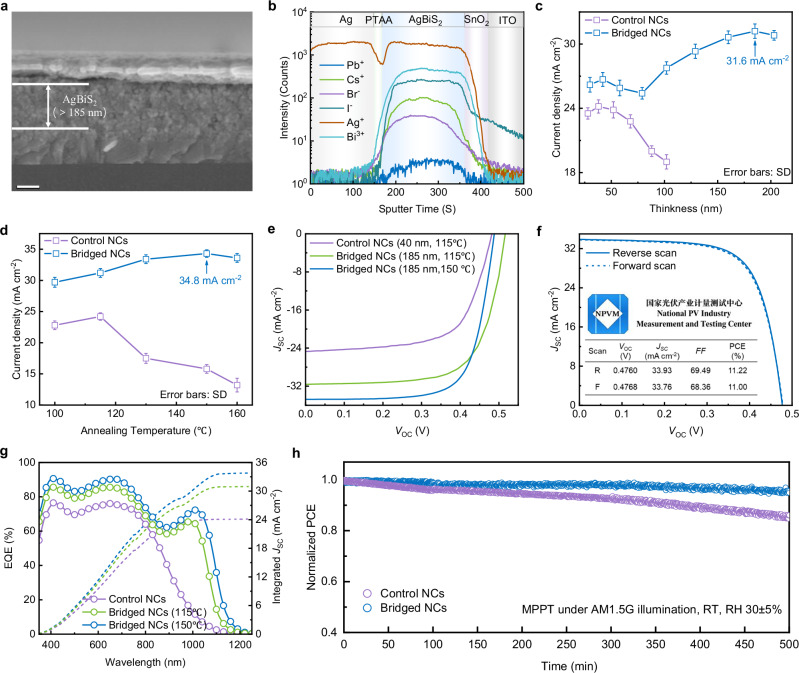
Photovoltaic performance of devices employing control NC and bridged NC films. **a** Cross-section scanning electron microscopy (SEM) image of a typical device structure. Scar bar, 500 nm. **b** Time-of-flight secondary ion mass spectrometry (TOF-SIMS) depth profile of solar cell device using bridged NC films. **c** *J*_SC_ of control NC and bridged NC devices as a function of active layer thickness, plotted with the average for the fifteen devices. The annealing temperature was fixed at 115°C. Error bars: standard deviation (SD). **d** *J*_SC_ of control NC and bridged NC devices as a function of annealing temperature, plotted with the average for the fifteen devices. The control NC and bridged NC films thickness was fixed to ~ 40 nm and ~ 185 nm, respectively. Error bars: SD. **e** *J-V* curves of champion control NC devices (~ 40 nm, annealing at 115°C) and bridged NC devices (~185 nm, annealing at 115°C or 150 °C). **f** Certified *J-V* curves of the champion bridged AgBiS_2_ NC devices. **g** External quantum efficiency (EQE) spectra (left) and integrated *J*_SC_ plots (right) of champion control NC devices (~ 40 nm, annealing at 115 °C) and bridged NC devices (~ 185 nm, annealing at 115°C or 150 °C). **h** Maximum power point tracking (MPPT) test for the control NC and bridged NC devices under continuous AM1.5 G illumination in ambient air and RT.

This outcome clearly demonstrates the potential of bridged NCs for fabricating thicker photoactive layers, thereby enhancing infrared-harvesting capability and boosting J_SC_. To verify this hypothesis, we investigated the relationship between film thickness and AgBiS_2_ NC ink concentration for both NC films, aiming to achieve thickness-controlled NC film solar cells (Supplementary Fig. 23 and Supplementary Table 9). Photovoltaic devices were fabricated with a structure comprising indium tin oxide (ITO)/SnO_2_/AgBiS_2_ NCs/PTAA/MoO_X_/Ag, as illustrated in Fig. 5a. Time-of-flight secondary ion mass spectrometry (TOF-SIMS) analysis demonstrates that within the entire bridged NCs film, which has a thickness of approximately 185 nm, the signals of Cs, Pb, and halide (Br, I) exhibit relatively stable, thereby indicating a homogeneous distribution of molecular perovskite layer within the bridged NCs film (Fig. 5b). The scanning electron microscopy (SEM) images and elemental mapping of the bridged NCs film in Supplementary Fig. 24 further corroborate these results. An optimal halide ratio of I to Br at approximately 5:1 was used in the ligand solution for device fabrication, complemented by the addition of 0.02 mmol CsPbBr_3_ (Supplementary Fig. 25). Control experiments with alternative ligand combinations demonstrate that the performance enhancement specifically stems from the designed molecular perovskite bridge structure, rather than from simple lead halide ligand passivation (Supplementary Table 10).

As shown in Fig. 5c, the initial annealing temperature was set at 115°C to examine the *J*_SC_’s dependence on the thickness of the photoactive layer. It was observed that the J_SC_ of the bridged NC devices was significantly higher than that of the control, despite having an almost equivalent photoactive layer thickness, indicating improved charge-carrier transport in the bridged NC devices. The control NC devices achieved the maximum *J*_SC_ of 24.7 mA cm^-^² at a thickness of approximately 40 nm of the photoactive layer, while the bridged NC devices exhibited a *J*_SC_ as high as 31.6 mA cm^-^² at approximately 185 nm of the active layer. The dip observed at approximately 80 nm can be attributed to optical interference effects, which align consistently with our theoretical predictions. The results demonstrate unequivocally that the bridged NCs enable the production of thicker, optimized NC films, thereby enhancing light harvesting. Furthermore, the passivation effect in the bridged NC devices exhibited a synergistic effect, resulting in enhanced *V*_OC_ and fill factor (FF) (Supplementary Fig. 26)^6^.

Subsequently, the optimized photoactive layer thicknesses of the control NC devices and bridged NC devices were fixed at approximately 40 nm and 185 nm, respectively, in order to study the performance of the two devices under different annealing temperatures (Fig. 5d and Supplementary Fig. 27). For the control NC devices, optimal performance was achieved at 115°C. The annealing at higher temperatures decreased its performance^1^. Conversely, the *J*_SC_ of bridged NC devices exhibited a continuous increase with rising annealing temperature, ultimately attaining a noteworthy *J*_SC_ of 34.8 mA cm⁻² at 150 °C. This enhancement can be attributed to an increase in light absorption, which is induced by elevated cation disorder (Fig. 1e). Notably, the *V*_OC_ of both devices exhibited a consistent decline with increasing annealing temperature. However, this decline was gentler for bridged NCs. This voltage loss is attributable to bandgap narrowing, which is itself caused by enhanced cation disorder^1,43^. This represents a trade-off, whereby disorder increases absorption at the expense of photo-voltage (Supplementary Fig. 28). Control NCs are confronted not only with this issue but also subject to defect states engendered by NC coalescence, leading to a precipitous *V*_OC_ decline. Combining the band gap obtained from the Tauc plot and ultraviolet photoelectron spectroscopy (UPS) measurements, the corresponding energy level diagram was plotted in Supplementary Fig. 29. It was found that the bridged NC films exhibited a transition from weak p-type to n-type characteristics, which is consistent with the electron-rich Ag and Bi observed in the XPS of bridged NC films (Supplementary Fig. 9).

Figure 5e shows the *J-V* curves of champion control NC and bridged NC devices. It was established that, at an annealing temperature of 115 °C, the PCE of devices with a thickness of approximately 185 nm, prepared from bridged NCs, exhibited an increase from 7.77% to 11.23%, in comparison with devices with a thickness of approximately 40 nm, prepared from control NCs. Subsequent optimization of the annealing temperature to 150 °C resulted in the bridged NCs device attaining a PCE of 12.13%, with a *V*_OC_ of 0.489 V, a *J*_SC_ of 34.8 mA cm^-^², and an FF of 71.3% (Table 1 for details). This establishes a record PCE for AgBiS_2_ NC-based solar cells, which show reproducible high performance (Supplementary Fig. 30 and Supplementary Table 11). One of the champion devices was dispatched to the National PV Industry Measurement and Testing Center for the purpose of obtaining certification, and the certified efficiency was found to be 11.22%, with negligible hysteresis (Fig. 5f and Supplementary Fig. 31). This result far exceeds the current highest certified PCE of 9.4%^10^. The discrepancy between the laboratory-measured efficiency and the certified efficiency may be attributed to the differences in measurement conditions.

**Table 1:** Photovoltaic parameters of AgBiS_2_ NC solar cell characterized under illumination of AM 1.5 G (100 mW cm^-2^) in reverse scan through a shadow mask with 0.022 cm^-2^ area^a^

Sample	PCE (%)	*V*_OC_ (V)	*J*_SC_ (mA cm^-2^)	FF (%)
Control NCs (40 nm, 115°C)	7.77 (7.43 ± 0.41)	0.482 (0.479 ± 0.004)	24.7 (24.2 ± 0.6)	65.3 (64.1 ± 0.9)
Bridged NCs (185 nm, 115°C)	11.23 (10.84 ± 0.37)	0.516 (0.512 ± 0.005)	31.6 (31.2 ± 0.7)	68.9 (67.9 ± 1.2)
Bridged NCs (185 nm, 150 °C)	12.13 (11.68 ± 0.53)	0.489 (0.486 ± 0.004)	34.8 (34.3 ± 0.6)	71.3 (70.1 ± 1.1)

^a^ Values in parentheses indicate average and standard deviation of each parameter collected from 15 devices.

The enhancement in *J*_SC_ was further validated by EQE analysis. The integrated *J*_SC_ values derived from the EQE spectra (Fig. 5g) show excellent agreement with those extracted from the *J*-*V* curves, with discrepancies of less than 3%. As shown in Fig. 5g, the bridged NCs device (115°C) exhibited an improvement in *J*_SC_ within the 350–750 nm range in comparison with the control NCs device. This enhancement was ascribed to the enhanced carrier transport in the bridged NCs film. The enhancement of *J*_SC_ is primarily attributable to the substantial improvement in EQE within the near-infrared region (800–1200 nm), far outside the absorption range of CsPbX_3_ perovskite. It is important to note that near-infrared light is generally absorbed in the rear part of a photovoltaic device^18^. Therefore, the extended diffusion length of the bridged NCs film is particularly suitable for the transport of the photoexcited carriers generated in this region, thus improving the *J*_SC_. Increasing the annealing temperature to 150 °C resulted in enhanced light absorption and carrier transport, thereby improving the EQE response. This ultimately leads to the attainment of an integrated *J*_SC_ of ~34 mA cm^-2^, signifying a substantial enhancement in efficiency. A red shift in the EQE spectrum was observed, thus confirming the reduction of bandgap.

The enhanced surface passivation and lattice anchoring of bridged NCs have been demonstrated to significantly improve the operational stability of the bridged NCs device^23^. Under continuous maximum power point tracking at 1 sun illumination in ambient air and room temperature, the bridged NCs device retained 95.2% of its initial PCE after 500 min, whereas the control device degraded to 83.5% of its initial value (Fig. 5h). The unencapsulated bridged NCs device exhibited 99.7% retention of its initial PCE after a 150-day storage period at 25-35% relative humidity and room temperature. Furthermore, the bridged NC devices also exhibited excellent stability under high humidity conditions (75–85%) over a one-month period (Supplementary Fig. 32). We further synthesized AgBiS_2_ using (TMS)₂S in conjunction with the implementation of our strategic approach for comparison, yielding a PCE of 10.76% (Supplementary Fig. 33). While exhibiting lower values than the monodisperse NCs examined in this study, it substantially surpasses conventional passivation methodologies, thereby demonstrating both universality and advancement.

## Discussion

In this study, we show a ligand-mediated heteroepitaxial strategy to address the long-standing thickness-efficiency paradox in the AgBiS_2_ NC solar cells. The preparation of bridged AgBiS_2_ NC films involves the growth of a molecular perovskite layer on the surface of NCs. This facilitates the utilization of a more substantial photoactive layer in solar cell devices and annealing at temperatures in excess of 150 °C, thereby markedly enhancing the infrared light response. As a result, the device with a 185 nm thick photoactive layer achieves a record certified PCE of 11.22% under AM1.5 G illumination, representing the highest value ever attained. We believe that the findings presented in this study signify a major step towards the development of efficient, sustainable, and environmentally friendly optoelectronics.

## Methods

### Materials

Silver acetate (Ag(OAc), 99.99%), bismuth acetate (Bi(OAc)_3_, 99.99%), sulfur (S, 99.99%, powder), Bis(trimethylsilyl) sulfide ((TMS)_2_S, 1-octadecene (ODE, >90%), oleic acid (OA, >90%), oleylamine (OLA, ≥98%), methylammonium iodide (MAI, >99%), methylammonium bromide (MABr, >99%), Silver(I) iodide (AgI, 99.999%), Silver(I) bromide (AgBr, 99.999%), N, N-dimethylformamide (DMF, 99.8%) were purchased from Sigma-Aldrich. The remaining chemicals are purchased from Xi’an Polymer Light Technology Corp.

### Synthesis of (TMS)_2_S Based AgBiS_2_ NCs

(TMS)_2_S based AgBiS_2_ NCs were synthesized by hot-injection method employing standard Schlenk techniques. 3.2 mmol of Ag(OAc), 4 mmol of Bi(OAc)_3_, 24 mL of oleic acid and 18 mL of ODE were stirred under vacuum at 90 °C for 1 h to remove oxygen and moisture. Then, the temperature of reaction vessel was raised to 100°C and further degassed for extra 2 h to form Bi and Ag oleate. Then, the atmosphere of reaction vessel was changed to N_2_ and 4 mmol of (TMS)_2_S dissolved in 2 mL of ODE was swiftly injected into the flask. The heating was immediately stopped by removing the heating mantle, and the reaction flask was rapidly cooled down to room temperature using a cold-water bath and stirred for 1 h. Synthesized AgBiS_2_ NCs were initially purified with acetone and centrifugation at 7500 × *g* for 3 min. Precipitated AgBiS_2_ NCs were dispersed in toluene and reprecipitated by adding acetone. This process was repeated two times and the final precipitate was dried overnight. Finally, the precipitated NCs were dispersed in anhydrous octane (20 mg mL^-1^).

### Synthesis of S-OLA Based AgBiS_2_ NCs

S-OLA based AgBiS_2_ NCs were synthesized by hot-injection method. 4 mmol of Ag(OAc), 3 mmol of Bi(OAc)_3_, 8 mL of OA, and 12 mL of ODE were stirred and degassed under vacuum at 90 °C for 2 h. Then, the temperature was raised to 140 °C and maintained under an N_2_ atmosphere. 4 mmol of S and 4 mL of OLA were added to another three-neck round-bottom flask. The mixture was heated to 100 °C under an N_2_ atmosphere and then kept under vacuum for 2 hours to complete degassing. After that, the temperature was raised to 150 °C under a N_2_ flow, and then 10 mL of the Ag-Bi oleate precursor solution was injected into the solution. The reaction mixture was maintained at 150 °C for an additional 20 minutes before the reaction flask was allowed to cool to room temperature. Synthesized AgBiS_2_ NCs were initially purified with methanol/acetone mixture (v/v, 1:1) and centrifugation at 7500 × *g* for 3 min. Precipitated AgBiS_2_ NCs were dispersed in toluene and reprecipitated by adding methanol/acetone mixture. This process was repeated two times and the final precipitate was dried overnight. Finally, the precipitated NCs were dispersed in anhydrous octane (20 mg mL^-1^).

### Preparation of AgBiS_2_ NCs ink through solution-phase ligand exchange

The same preparation process was conducted to both control and target inks, except ligand solutions. For the control ligand solution, 1 mmol of AgI, 1 mmol of MAI, 0.2 mmol of AgBr and 0.2 mmol of MABr were dissolved in 10 mL DMF. For the P-modified ligand solution, 1 mmol of AgI, 1 mmol of CsI, 0.2 mmol of AgBr, 0.22 mmol of CsBr, and 0.02 mmol of PbBr_2_ were dissolved in 10 mL DMF. Next, 5 mL of OA/OLA-AgBiS_2_ NCs solution (6 mg mL^-1^) was added to an equal volume of prepared ligand solutions and vigorously shaken for 3 min. After the occurrence of phase separation between octane and DMF, the octane phase on the top of the solutions was discarded. The AgBiS_2_ NCs dispersion in the DMF ligand solution was further washed twice with pure octane. Subsequently, toluene was added, and the solution was centrifuged. The precipitated AgBiS_2_ NCs were finally dispersed in DMF.

### Device fabrication

Unless otherwise specified, the fabrication of the solar cell device was performed in ambient air. Highly transparent ITO were prepared via magnetron sputtering (MS) with an optimized O_2_/Ar ratio (Supplementary Fig. 34a). The sputtering rate is at 2 Å s^-1^ under room temperature. The MS ITO-coated glass substrates were ultrasonically cleaned in soapy water, acetone, and isopropanol for 20 minutes each, and then dried with nitrogen. This was followed by ultraviolet/ozone treatment for 0.5 hours. The SnO_2_ electron transport layer was then spin-cast from diluted Alfa SnO_2_ colloid solution (1:1 v/v with H_2_O) at a rotation speed of 3500 rpm and annealed at 150 °C for 30 min (Supplementary Fig. 34b). Then, as-synthesized AgBiS_2_ NCs ink solution (200 mg mL^-1^ for control NCs and 450 mg mL^-1^ for bridged NCs) was spin-casted at 3000 rpm for 30 s. For the control NC films, the substrates were sequentially transferred into an N_2_-filled glove box and annealed at 115 °C for 5 minutes. For the bridged NC films, the substrates were annealed at 150 °C for 5 minutes. Then, the bridged NC films were rinsed with ACN. After assembling AgBiS_2_ NC films, a buffer layer of 1,2-ethanedithiol (EDT)-capped AgBiS_2_ NCs, prepared via the SSLE method, was subsequently deposited on top. The resultant AgBiS_2_ NC films were annealed at 80 °C in the glove box and then stored in dry air overnight before spin-coating PTAA solution (3 mg ml^-1^ in toluene) at 3000 rpm for 30 s. Finally, 3 nm MoO_3_ and 120 nm Ag were deposited by thermal evaporation under vacuum at 2 × 10^-5 ^Torr.

### Characterization

The details of characterizations are described in Supporting Information.

### Reporting summary

Further information on research design is available in the Nature Portfolio Reporting Summary linked to this article.

## Supplementary information

Supplementary Information

Reporting Summary

Transparent Peer Review file

## Data Availability

The experimental data that support the findings of this study are available in a public repository^44^. (10.6084/m9.figshare.31122340).
