# Supplementary material for: Molecular lead halide perovskite layer bridged AgBiS2 nanocrystals for efficient thin film solar cells
Source: Nat Commun. 2026 Apr 24;17:5687. doi: 10.1038/s41467-026-72272-4 (PMC13319432; doi:10.1038/s41467-026-72272-4)
Supplement: Supplementary file 1 — Supplementary Information [file 41467_2026_72272_MOESM1_ESM.pdf]

Supplementary Information for

Molecular Lead Halide Perovskite Layer Bridged $AgBiS_2$ Nanocrystals for Efficient Thin Film Solar Cells

Wanpeng Yang [1†], Tianyu Sun [1,†], Haixuan Yu [1], Haodan Shi [1], Yong Hu [1], Junyi Huang [1], Zhirong Liu [1], Ying Xu [1], Lei Wang [2], Bing Hu [1,2], Yan Shen [1], Mohammad Khaja Nazeeruddin [3,4], and Mingkui Wang [1,5,*]

[1] Wuhan National Laboratory for Optoelectronics, School of Optoelectronic Science and Engineering, Huazhong University of Science and Technology, Wuhan, Hubei, 430074, P. R. China

[2] Wuhan Hero Optoelectronics Technology Co., LTD, 6 Huanglongshan North Road, East Lake High-Tech Development Zone, Wuhan, Hubei, 430070, P.R. China

[3] Institut des Sciences et Ingénierie Chimiques, Ecole Polytechnique Fédérale de Lausanne, Lausanne, 1015, Switzerland

[4] Mechanical and Energy Engineering Department, College of Engineering, Imam Abdulrahman Bin Faisal University; Dammam, 34212, Saudi Arabia

[5] Optics Valley Laboratory, Wuhan, Hubei, 430074, P.R. China.

[†] Contribution equally

* Corresponding author E-mail: mingkui.wang@mail.hust.edu.cn

Supplementary Methods

Characterization

The X-ray diffraction spectrometry pattern was measured using a Shimadzu XRD-6100 diffractometer (CuK radiation). XPS measurements were performed using a Nexsa instrument (Thermo Fisher). The Femi energies and valence band edges of the materials were determined by ultraviolet photoelectron spectroscopy (UPS, Escalab 250Xi, Thermo Fisher). The optical measurements for the films were examined using a UV spectrophotometer (MAPADA UV-6100s) operating in the ultraviolet-visible-near infrared (UV-Vis-NIR) range. PL was measured by a fluorescence spectrophotometer (LabRAM HR800). The GIWAXS and GISAXS measurement were carried out with the Xeuss 3.0. The incident angle was 0.2°. The HR-TEM images were obtained with a high-resolution transmission electron microscope (JEM-2100F, Japan) in high-resolution mode. The photocurrent density–voltage (*J*–*V*) characteristics of devices were measured under 1 sun illumination using a programmable Keithley 2400 digital source meter under AM1.5 G simulated sunlight at 100 mW cm^{-2} (Oriel, model 91192). The intensity of the simulated light was precisely calibrated using an NREL-certified KG5-filtered Si photodiode detector. FTIR spectra were measured using an FTIR spectrometer with a diamond ATR (Nicolet iS50R, Thermo Scientific). The cross-section of the device was imaged using an in-lens secondary electron detector of a Zeiss Auriga FIB-SEM microscope operated at 5 kV. The mask area was measured by Automatic Image Measuring Instrument.

Diffusion length analysis

we determined the diffusion coefficient through the Einstein relation:

$$D_n = \frac{kT}{q}\mu_n \quad \text{(S1)}$$

where D_n is the diffusion coefficient, k is the Boltzmann constant, q is the elementary charge, T is the temperature, and μ_n is carrier mobility. By substituting the measured lifetime and the calculated diffusion coefficient into the formula:

$$L_n = \sqrt{D_n \tau_n} \quad \text{(S2)}$$

where L_n is the carrier diffusion length, D_n is the diffusion coefficient, and τ_n is the

carrier lifetime, we can obtain the carrier diffusion length.

Activation energy analysis

The activation energy (E_a) of bridged NC films could be obtained according to the Arrhenius equation:

$$\mu = \mu_0 \exp\left(-\frac{E_a}{k_B T}\right) \quad (S3)$$

where k_B and μ_0 are Boltzman constant and Pre-exponential factor, respectively.

Space-charge-limited current (SCLC) method

SCLC measurement was conducted based on electron-only devices with an architecture of ITO/SnO_2/$AgBiS_2$/PCBM/Ag and hole-only devices with the structure of ITO/ PEDOT:PSS/$AgBiS_2$/PTAA/Ag, which was measured in a dark environment using a Keithley 2400 source meter. The applied voltages started from 0 V and rose to 4 V. Three regions were evident in the SCLC data. *I–V* characteristics show three different regions: a linear ohmic region at low voltage, a trap filling region from mediate voltage to the trap filled limit voltage (V_{TFL}), a Child's region, respectively. The relationship between the defect density (N_{trap}) and the onset voltage of the trap-filled limit (V_{TFL}) of the devices is shown as follows:

$$N_{trap} = \frac{2\varepsilon_0 \varepsilon V_{TFL}}{qL^2} \quad (S4)$$

where ε_0 and ε represent the vacuum permittivity (8.8542×10^{-14} F cm^{-1}) and the relative dielectric constants of $AgBiS_2$, respectively. L is the thickness of the $AgBiS_2$ film, and q is the elementary charge.[1]

The electron/hole mobility was calculated by SCLC measurement using Mott-Gurney formula:

$$J = \frac{9\mu_e \varepsilon_0 \varepsilon V^2}{8L^3} \quad (S5)$$

where J is the current density from the SCLC region, μ_e is the electron mobility, ε_0 and ε represent the vacuum permittivity (8.8542×10^{-14} F cm^{-1}) and the relative dielectric constants of $AgBiS_2$, respectively, V is the applied voltage and L the thickness of the $AgBiS_2$ film.

Time-of-flight (TOF) measurements

TOF measured using a nanosecond laser with a wavelength of 520 nm. The electron-only devices and hole-only devices were fabricated using the same device architecture as described in Section 2.2. The thickness of $AgBiS_2$ was regulated over 250 nm and evaluated with thickness profilometer. Photocurrent decay was recorded with varying biases using an oscilloscope. Carrier mobility (μ) was calculated using the formula:

$$\mu = \frac{L^2}{V t_{tr}} \quad \text{(S6)}$$

where L is the thickness of $AgBiS_2$ NCs film and V is bias voltage, and t_{tr} is the transit time. The extracted t_{tr} under each bias condition was plotted L^2/t_{tr} versus V.

Computational Method

Spin-polarized DFT calculations were performed using the projector-augmented wave pseudopotential,[2] as implemented in the Vienna Ab initio simulations package,[3] to obtain the electronic energies and relaxed atomic structures of the $AgBiS_2$ crystals. The geometry was optimized using the conjugate gradient method, and the Hellmann–Feynman forces on all unfixed atoms were reduced to <0.005 eV/Å. The surface models of the $AgBiS_2$ crystals were constructed for the (100) and (111) slabs with a vacuum gap of at least 20 Å between the slabs to avoid artificial interactions between the adjacent cells along the plane-normal direction. The crystal structure diagram was created by VESTA.[4] $AgBiS_2\{100\}/CsPbBr_3\{100\}$ and $AgBiS_2\{111\}/CsPbBr_3\{111\}$ systems with 14 atomic layers in total and a vacuum layer of 15 Å were used for interface calculations. The interfacial energy (E_{intf}) widely used to describe interface properties, and E_{intf} could be determined by the following definitions[5]:

$$W_{ad}(AgBiS_2\text{-}CsPbBr_3) = [E_{slab}(AgBiS_2) + E_{slab}(CsPbBr_3) - E_{slab}(AgBiS_2\text{-}CsPbBr_3)]/A \quad \text{(S7)}$$

$$E_{intf} = [E_{surface}(AgBiS_2) + E_{surface}(CsPbBr_3) - W_{ad}(AgBiS_2\text{-}CsPbBr_3)]/A \quad \text{(S8)}$$

where $W_{ad}(AgBiS_2\text{-}CsPbBr_3)$ was defined as the reversible work to separate an interface into two free surfaces, A was the interface area, $E_{slab}(AgBiS_2)$ and $E_{slab}(CsPbBr_3)$ were the energies of the relaxed $AgBiS_2$ and $CsPbBr_3$ slab respectively, $E_{slab}(AgBiS_2\text{-}CsPbBr_3)$ was the energy of the relaxed $AgBiS_2\text{-}CsPbBr_3$ interfacial structure, and

$E_{surface}(AgBiS_2)$ and $E_{surface}(CsPbBr_3)$ were surface energies of the corresponding surfaces.

Optical Modelling

A self-developed Python script was employed to conduct the transfer matrix calculation. During the calculation, each layer was assumed to be optically flat, and scattering effects were neglected. The short-circuit current density was estimated under the assumption that the internal quantum efficiency was 100%.

$$J_{sc} = e\int_0^{+\infty} \mathrm{EQE}_{\mathrm{TMM}} \times \emptyset_{AM1.5G}(E)dE \quad \text{(S9)}$$

where EQE_{TMM} is the estimated total absorption in the $AgBiS_2$ NCs layer and ∅AM1.5G is the solar spectra AM1.5G.

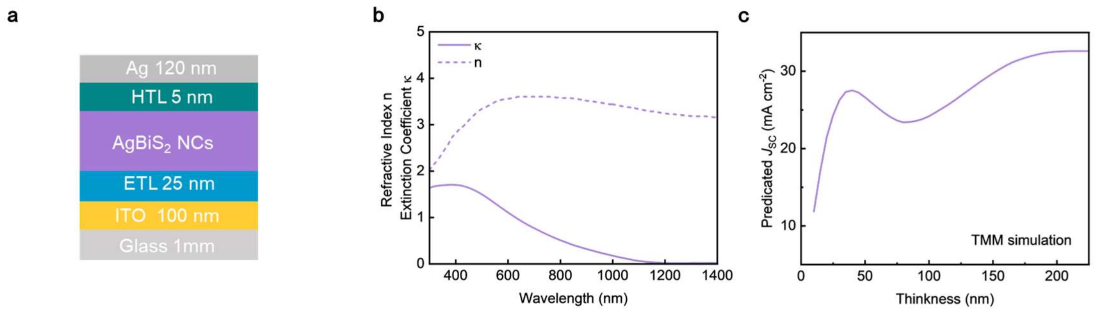

Supplementary Figure 1. Optical modelling via the Transfer Matrix Method (TMM). (a) Device structure used for TMM calculations. (b) Refractive indices *n* (dashed lines) and extinction coefficients *κ* (solid lines) of $AgBiS_2$ NC films (annealing at 115°C) measured by ellipsometry. (c) Predicted J_{SC} of $AgBiS_2$ NCs using the TMM as a function of film thickness.

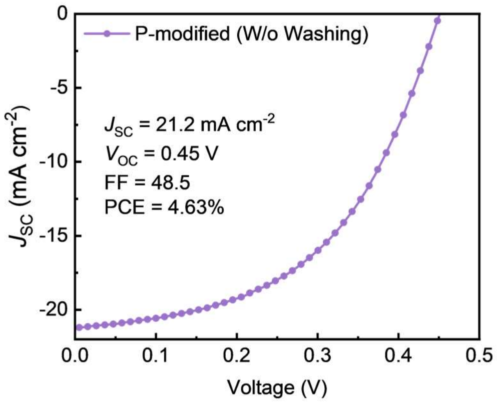

Supplementary Figure 2. Photovoltaic performance of devices employing perovskite modified NCs without washing with ACN. The thick perovskite matrix in the NC film strongly impedes charge transport and compromises device performance.

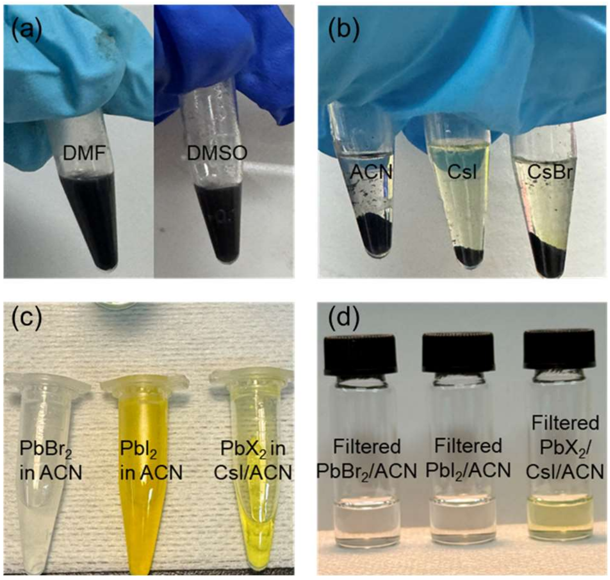

Supplementary Figure 3. (a) AgX+PbX capped $AgBiS_2$ NC solids merged in pure DMF and DMSO solvent, respectively. Both solvents completely dissolved the NC solids. (b) AgX+PbX capped $AgBiS_2$ NC solids merged in pure ACN solvent (left), CsI/ACN (middle, 5mg/mL), and CsBr/ACN (right, 5mg/mL); this indicates that ACN does not dissolve PbX ligand. To verify the insolubility of PbX ligands on the NC surface in ACN, we replaced CsX in the ligand solution with NaX to eliminate interference from color changes caused by perovskite dissolution. (c) 20mg $PbBr_2$ (left) pellets, 20mg PbI_2 (middle) pellets in 1 mL of ACN solvent and CsI/ACN (5mg/mL) after 30 s vortex; this indicates that CsI /ACN dissolves PbX_2 and dynamically converts into perovskite. (d) Colorless solution of $PbBr_2$ (left) and PbI_2 (middle) in ACN after filter, transparent light-yellow solution of PbX_2 in CsI/ACN (right) after filter. (c) and (d) indicate that ACN can partially dissolve perovskite.

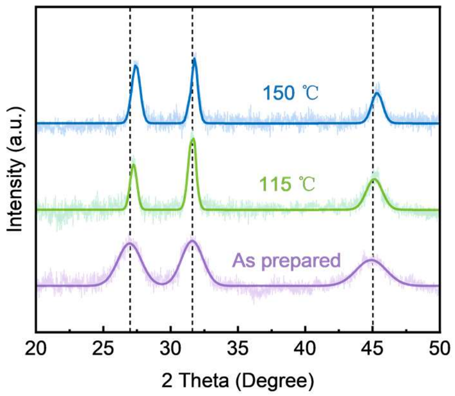

Supplementary Figure 4. XRD patterns of the $AgBiS_2$ NCs films at various annealing temperatures. A cation disorder-induced contraction of the average bond length is evidenced by a consistent shift of the diffraction peaks to higher angles.

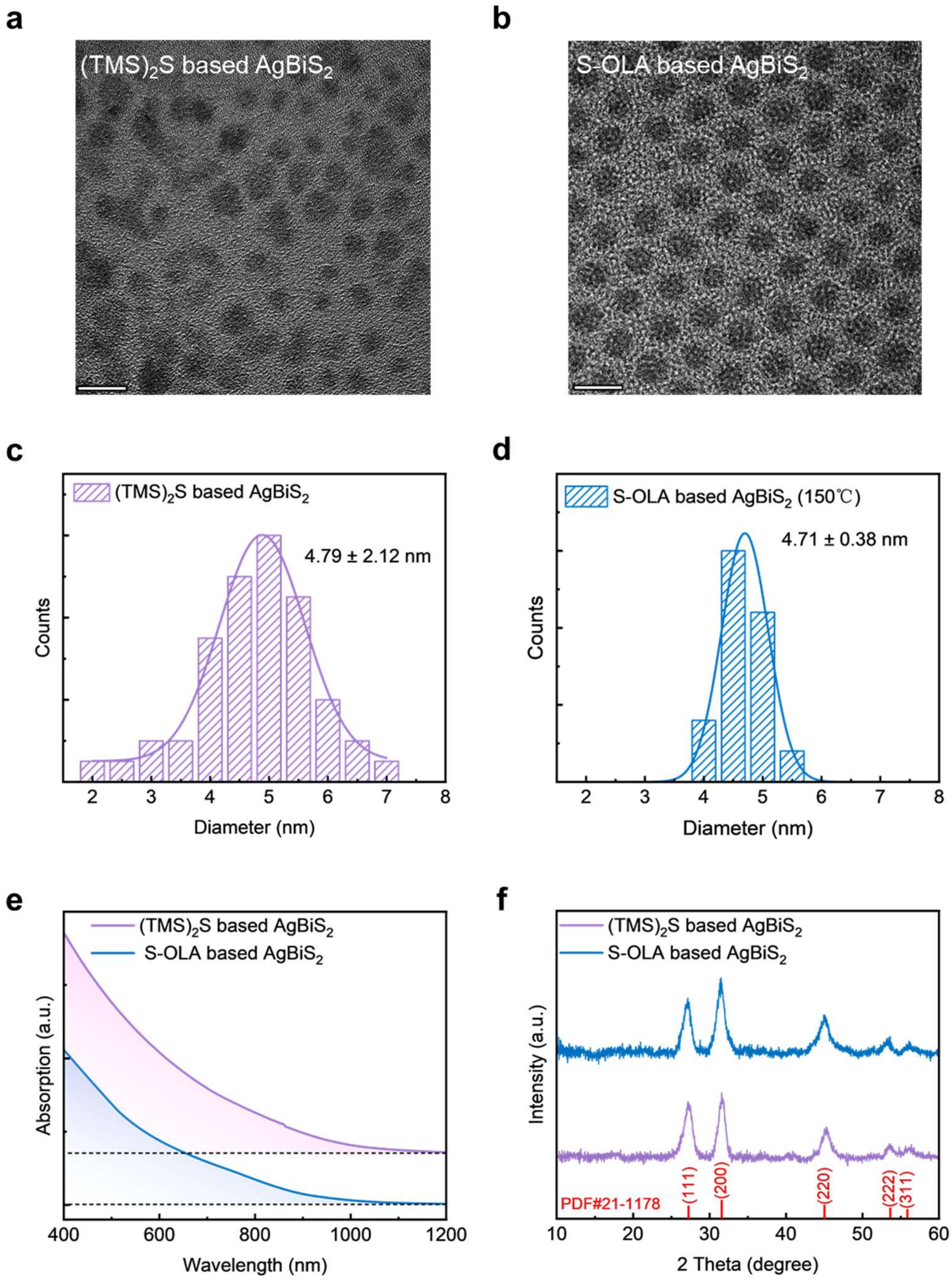

Supplementary Figure 5. (a-d) HRTEM images of $(TMS)_2S$-based $AgBiS_2$ NCs and S-OLA-based $AgBiS_2$ NCs, with their corresponding size distribution statistics. Scale bar: 5 nm. The significant narrowing of the size distribution indicates the monodispersity and uniformity of S-OLA-based NCs. (e) XRD patterns and (f) Absorption spectra of $(TMS)_2S$-based $AgBiS_2$ NCs and S-OLA-based $AgBiS_2$ NCs. Both NCs exhibit a cubic phase (*Fm-3m*).

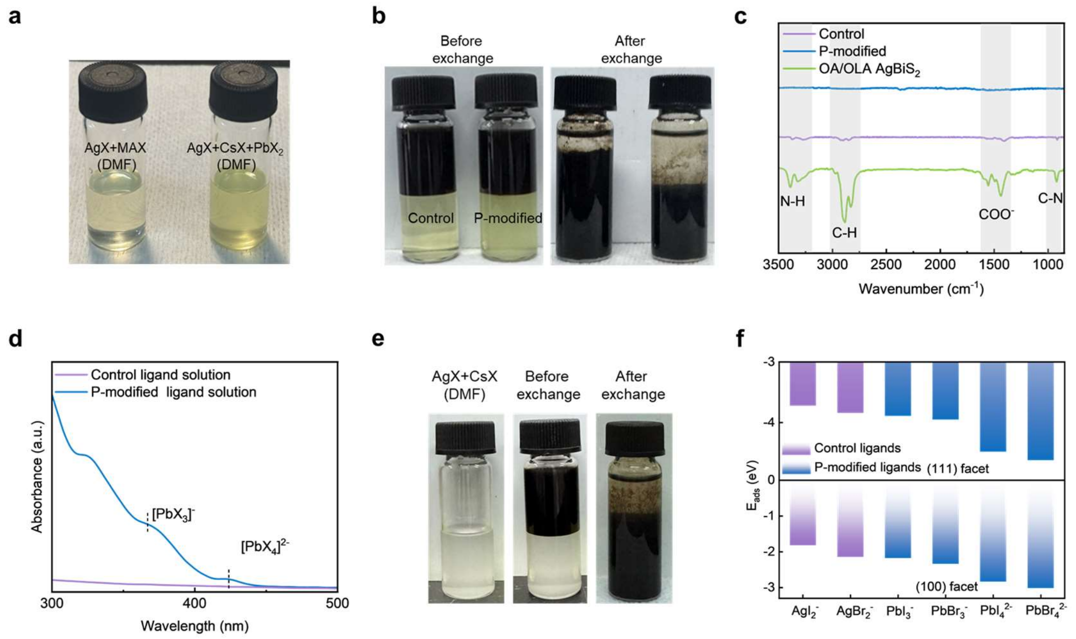

Supplementary Figure 6. The enhanced SPLE efficiency of the P-modified NCs ink. (a) The images of control and P-modified ligand solutions. (b) SPLE process images before and after using the control and P-modified ligands. (c) FTIR spectra of control and P-modified $AgBiS_2$ NCs inks, and oleic acid (OA)/OLA capped $AgBiS_2$ NC films. (d) UV-vis absorption spectroscopy of the control and P-modified ligand solutions. (e) The images of the SPLE process before and after using the AgX + CsX (X = I, Br) ligand systems. The phase transfer from octane to DMF is insufficient. (f) The adsorption energies (E_{ads}) of $[AgI_2]^-$, $[AgBr_2]^-$, $[PbI_3]^-$, $[PbBr_3]^-$, $[PbI_4]^{2-}$ and $[PbBr_4]^{2-}$ ligands onto (100) and (111) surfaces, respectively. The corresponding Density Functional Theory (DFT) calculation model is shown in Supplementary Fig. 7.

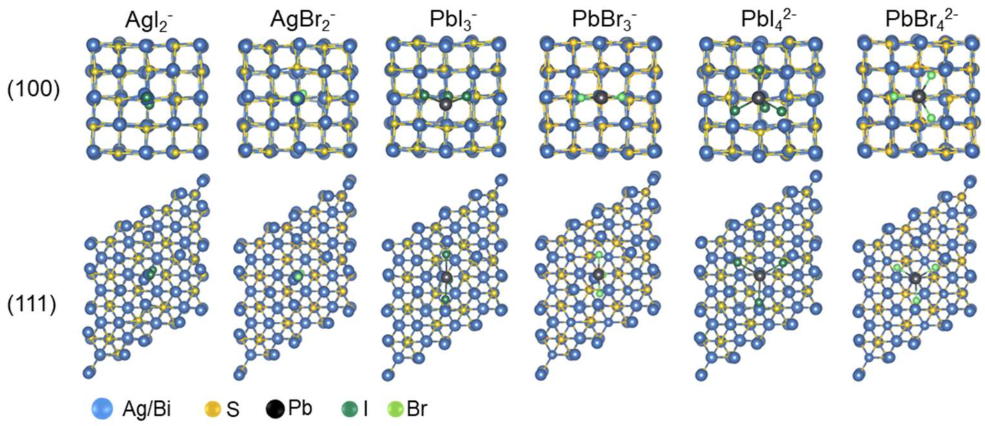

Supplementary Figure 7. The DFT calculation models of $[AgI_2]^-$, $[AgBr_2]^-$, $[PbI_3]^-$, $[PbBr_3]^-$, $[PbI_4]^{2-}$and $[PbBr_4]^{2-}$ adsorption onto (100) and (111) facets, respectively. The calculated values are listed in Supplementary Table 1. The blue, orange, black, dark-green and light green spheres represent the Ag/Bi, S, Pb, I and Br, respectively.

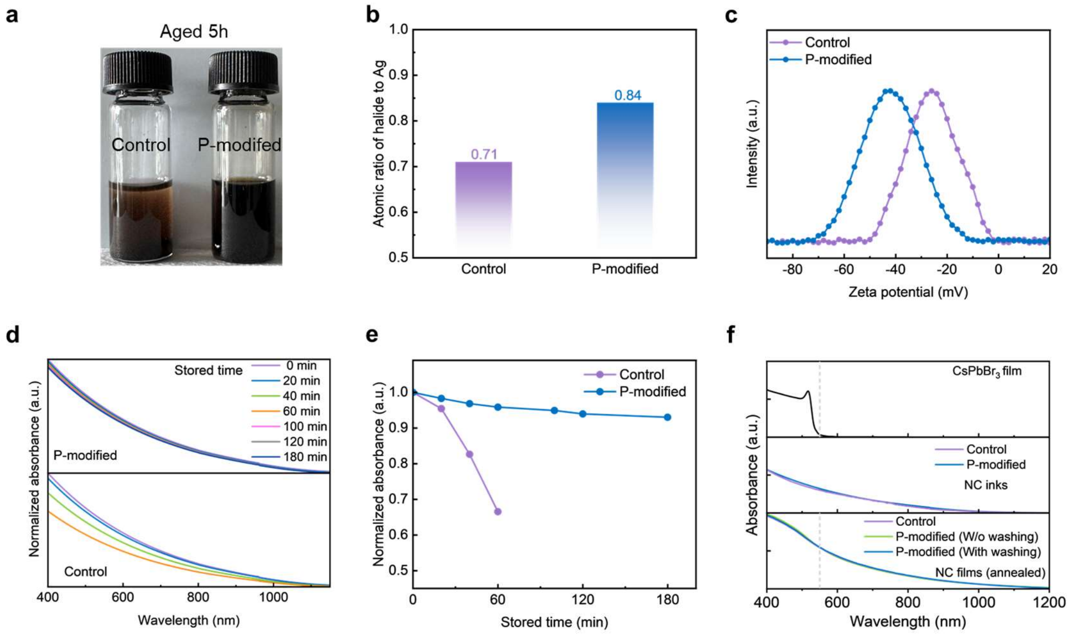

Supplementary Figure 8. The enhanced colloidal stability of the P-modified NCs ink. (a) The optical images of the P-modified NCs ink and the control NCs ink after 5 h storage at room temperature. (b) The ratio of halide to Ag atoms obtained from the XPS data (Supplementary Table 2) on the control and P-modified NCs films. (c) Zeta potential of the control and P-modified NCs ink. Typically, a zeta potential below −30 mV ensures stable dispersion of NCs in polar solvents via electrostatic repulsion.[6] (d-e) Full absorbance spectra of the control and P-modified NC inks at different storage times, and the changes in their respective normalized absorbances at a wavelength of 900 nm over time. The P-modified NC inks retained 95% of their initial absorbance after 180 min, whereas the control NC inks degraded to 66% within 60 min. (f) (Top) Absorption spectra of $CsPbBr_3$ perovskite films. (Middle) Absorption spectra of the control and P-modified NC inks in the freshly prepared state. The almost identical UV-Vis spectra can be attributed to the fact that the perovskite precursors are unable to adsorb on the surface of NCs in the form of photoactive phase, and the amount of adsorber is too small to affect absorption. (Bottom) Comparison of the light absorption between the P-modified NC film before and after washing, and the control NCs film. All samples were annealed at 150°C.

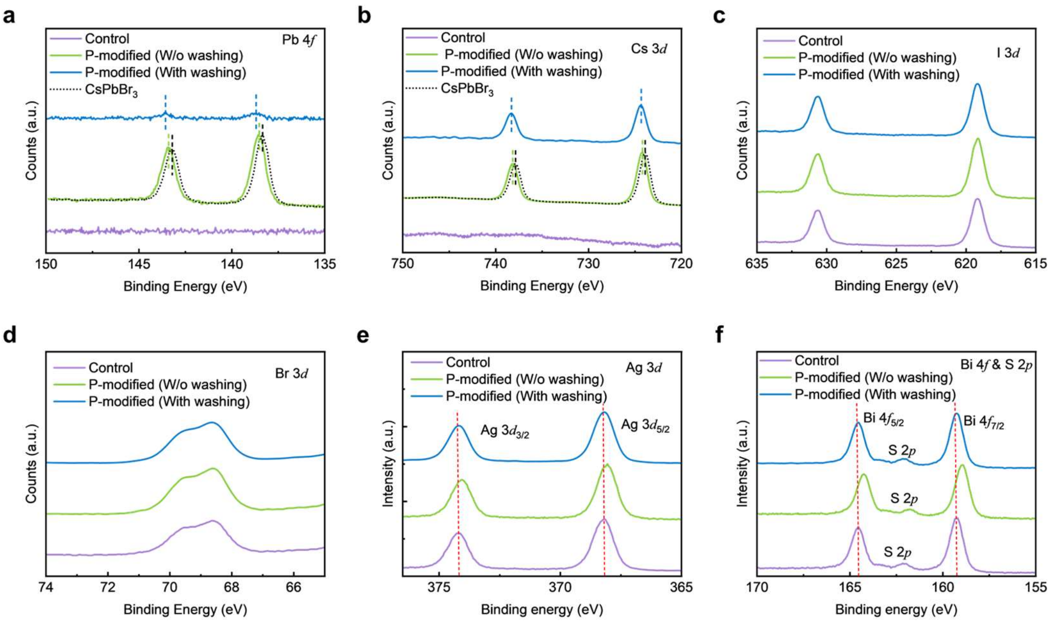

Supplementary Figure 9. High-resolution XPS spectra of (a) Pb 4*f*, (b) Cs 3*d*, (c) I 3*d*, (d) Br 3*d*, (e) Ag 3*d*, and (f) Bi 4*f* & S 2*p* for the control NCs film, P-modified NC films with/without ACN washing. Supplementary Fig. 9a-b additionally display the Cs 3*d* and Pb 4*f* XPS signals from a pure $CsPbBr_3$ thin film for reference. Comparison with the corresponding peaks in the P-modified NC films reveals that both the Cs and Pb signals in the latter shift toward higher binding energies. This observation confirms the interaction between the molecule perovskite layer and $AgBiS_2$ NCs. Furthermore, the binding energies of Ag 3*d* and Bi 4*f* in the P-modified NC films shift significantly to lower values compared to those in the control NC films. This shift is attributed to Ag and Bi atoms becoming electron-rich due to the introduction of perovskite within the NC films. These results collectively demonstrate the modified electronic environment at the interface.

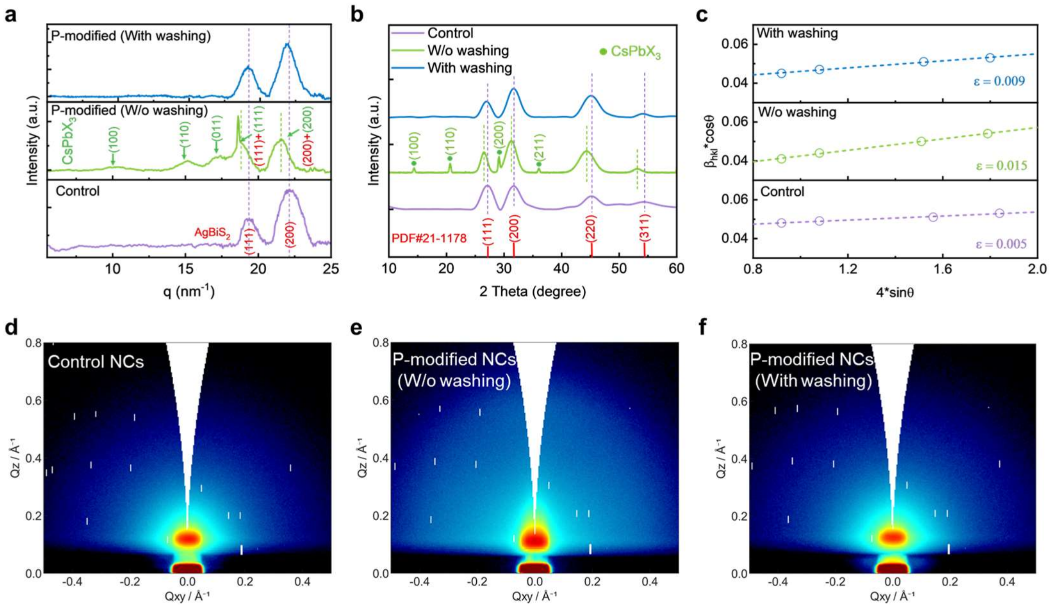

Supplementary Figure 10. (a) GIWAXS line profiles of the control NCs film, P-modified NC films with/without ACN washing. The shift of the patterns (dash line marked, corresponding to $AgBiS_2$ diffraction) to lower q values in P-modified NC films with /without ACN washing suggests the $CsPbX_3$ perovskite layer growth on NC surfaces, forming the strain due to the lattice mismatch between perovskite and $AgBiS_2$, where the lattice constant of $CsPbBr_3$ (5.85 Å) and $CsPbI_3$ (6.21 Å) have larger value than $AgBiS_2$ (5.69 Å). (b) XRD results of control NC films and P-modified NC with /without ACN washing films. (c) Williamson-Hall plot of control NC films and P-modified NC films with /without ACN washing. Williamson-Hall (W-H) method: $\beta_{hkl} * \cos\theta = \varepsilon * 4\sin\theta + \frac{k\lambda}{d}$, where β_{hkl} is the angular line width at half of the maximum intensity, ε is the lattice strain of the nanocrystal, λ is the wavelength of the X-ray (CuK_α) radiation used and is 0.15406 nm, θ is Bragg diffraction angle, k is the sharp factor (k=0.94), and d is the average crystallite size.[7] The calculated ε for the control NC films, P-modified NC films without ACN washing and P-modified NC films with ACN washing are 0.005, 0.015, and 0.009, respectively. (d-f) GISAXS 2D pattern of control NC films (d), P-modified NC films without washing (e) and with washing (f). The horizontal linecut GISAXS profiles of the corresponding NC films are presented in Fig. 2d. The inter-NC distance (d) was calculated using the formula $d = 2\pi/q$, q is the peak positions in the I(q) curve exhibited in Fig. 2d.

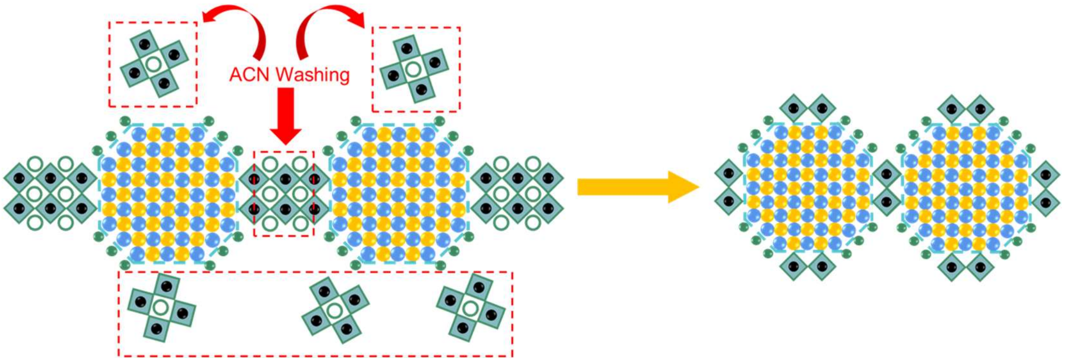

Supplementary Figure 11. Schematic illustration of ACN washing for dissolving excess perovskite matrix. During the washing process, any multilayer perovskite and independently existing perovskite matrix have been effectively dissolved. The lattice anchoring effect between the single-layer perovskite and the adjacent $AgBiS_2$ surfaces stabilizes the perovskite and prevents its dissolution. This solubility difference can be used to retain the single molecular layer.

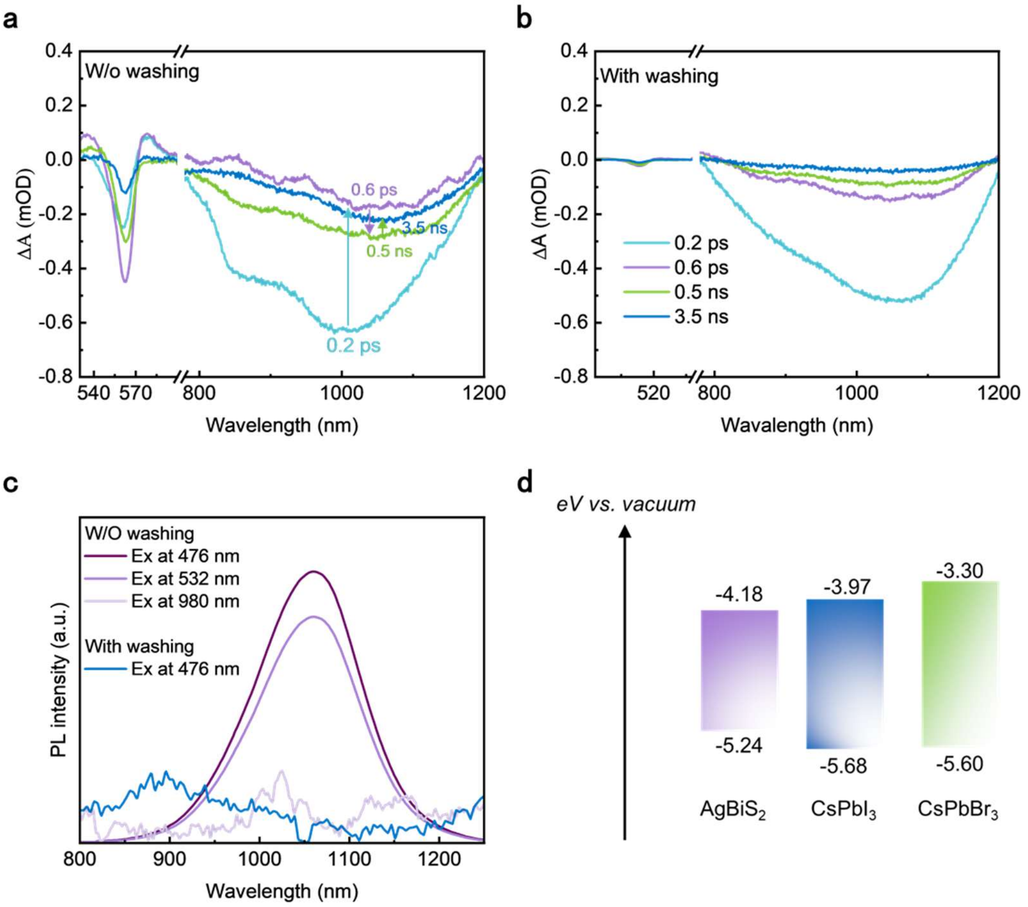

Supplementary Figure 12. TA spectra of P-modified NC films (a) with ACN washing, (b) without ACN washing. (c) Photoluminescence (PL) spectra of the P-modified films with and without washing under different excitation wavelengths. (d) Bandgap edge position of $AgBiS_2$ NCs[8], α-$CsPbI_3$[9], and α-$CsPbBr_3$ perovskite[10] from references.

Supplementary Note 1

We employed ultrafast TA spectra ranging from 0.1 ps to 3.5 ns, to investigate the transient carrier dynamics. In this analytic process, TA spectra with 0.2 ps (Cyan line), 0.6 ps (Purple line), 0.5 ns (green line), and 3.5 ns (blue line) were selected. A 400 nm pump laser was used to excite the ground-state electrons in both $CsPbBr_3$ and $AgBiS_2$ domains, enabling them to initially occupy high-density energy states. Supplementary Fig. 12a shows the evolution of TA signals for the P-modified NC without washing. The TA spectra of $CsPbBr_3$ exhibit strong GSB signals in the range of 545–585 nm (centered around 560 nm). The broadband GSB signals at 780–1200 nm originates from the $AgBiS_2$ domain, consistent with the reported GSB signals of $AgBiS_2$ NCs. We fixed

the ordinate range from −0.8 to 0.4 mOD to track the transient bleach decay of the $CsPbBr_3$ and $AgBiS_2$ domains. The GSB signal of the $CsPbBr_3$ domain reached a negative maximum at 0.6 ps, followed by a continuous recovery process as the excited-state electrons returned to the ground state. The GSB signal of the $AgBiS_2$ domain reached a negative maximum within 0.2 ps, rapidly recovered from 0.2 to 0.6 ps, and then exhibited a new negative maximum from 0.6 ps to 0.5 ns. This new negative maximum in the $AgBiS_2$ GSB signal can be attributed to the transfer of conduction band (CB) electrons from $CsPbBr_3$ to $AgBiS_2$, which attributed to the formation of a type-I heterojunction structure between $CsPbX_3$ perovskite and $AgBiS_2$ NCs (Supplementary Fig. 12d). The increased electron density in the CB of $AgBiS_2$ reduces the absorption of the probe laser, leading to an enhanced GSB signal. In contrast, as shown in Supplementary Fig. 12b, the extremely low perovskite content in the washed NC films was insufficient to alter the absorption properties of $AgBiS_2$ NCs, and no carrier transfer phenomenon was observed.

Subsequently, we measured the photoluminescence (PL) spectra of the p-modified NC films with and without washing. Extensive prior studies have demonstrated that $AgBiS_2$ typically does not exhibit PL at room temperature, primarily due to its indirect bandgap characteristics. However, under excitation with 476 nm and 532 nm lasers, our unwashed p-modified NC films displayed significant PL within the 800–1200 nm wavelength range. No PL signal was observed when using a 980 nm laser excitation. In this study, the bandgap of $CsPbX_3$ perovskite was determined to lie between those of $CsPbI_3$ (1.71 eV) and $CsPbBr_3$ (2.33 eV). Consequently, both 476 nm and 532 nm lasers possess sufficient energy to simultaneously excite $CsPbX_3$ and $AgBiS_2$ NCs. Combined with the Type-I band alignment configuration, excitons generated in the perovskite material transfer to adjacent $AgBiS_2$ NCs, where they undergo radiative recombination, thereby enhancing the PL emission from $AgBiS_2$. The 980 nm laser lacks the energy required to excite $CsPbX_3$ perovskite, preventing additional exciton generation and resulting in the absence of PL. Notably, after washing, the p-modified NC films

exhibited no detectable PL even under 476 nm laser excitation. This observation can be attributed to the drastic reduction of perovskite content leading to insufficient generation and transfer of excitons, resulting in a negligible contribution to the PL.

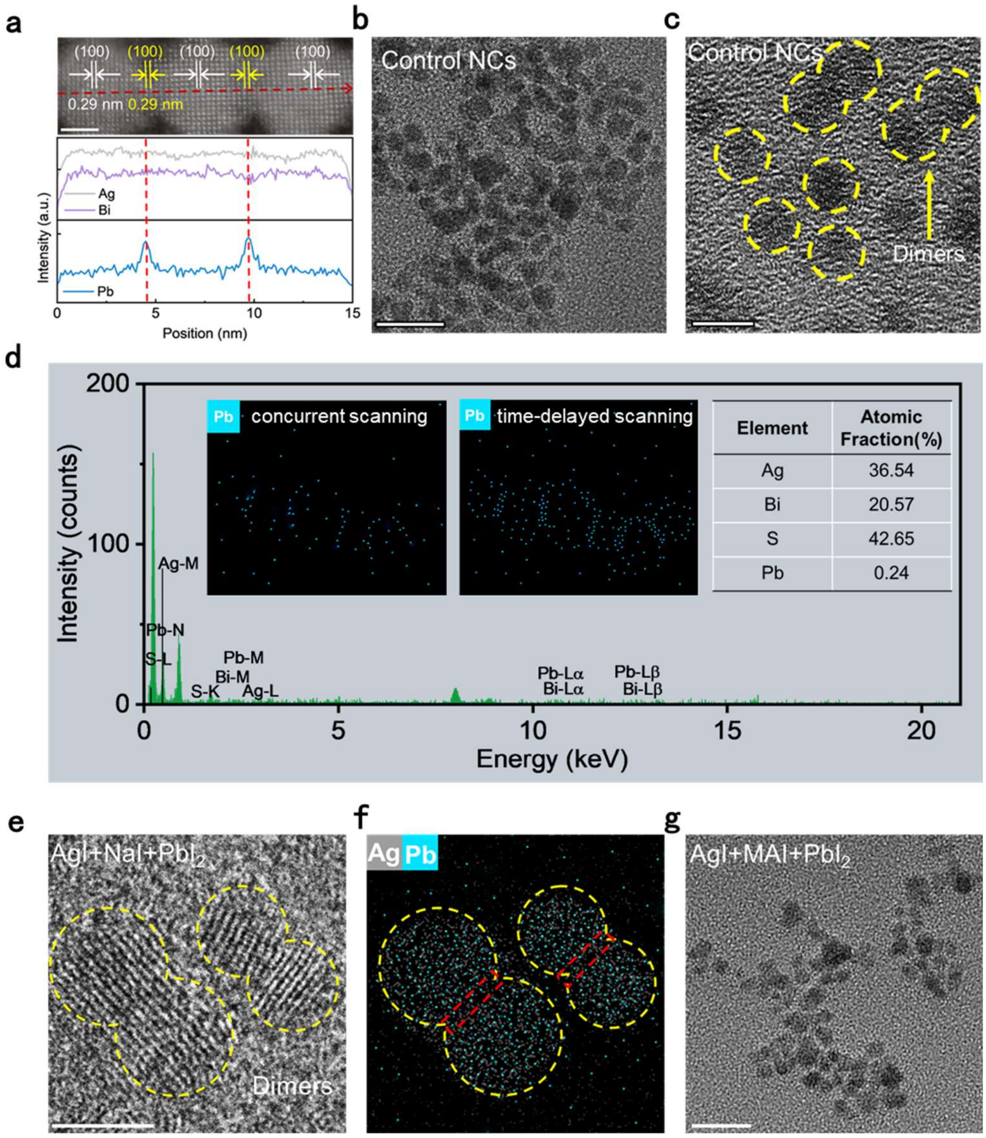

Supplementary Figure 13. (a) Dark-field scanning TEM image of bridged NCs and EDS line scanning of Ag, Bi, Pb elements. Pb accumulates at the bridging region. Scale bars: 2 nm. HRTEM images of the (b) Control NCs, and (c) Dimers. Scale bars: 20 nm and 5 nm, respectively. (d) EDS analysis of P-modified NCs after washing with ACN. The inset presents EDS mapping of Pb scanned simultaneously with Ag and Bi, along with time-delayed EDS mapping of Pb (adapted from Fig. 3c). The table shows the atomic percentages of each element during simultaneous scanning. Compared to the Ag content in the $AgBiS_2$-OA/OLA sample before ligand exchange (Supplementary Table 2), the significant excess of Ag is attributable to the presence of silver halide ligands adsorbed on the NC surface. (e) HRTEM image of the NCs dimer subjected to ligand exchange with (AgI+NaI+PbI_2). This particular ligand combination does not permit the

formation of a perovskite phase, but does allow for the adsorption of lead halide ligands onto the NC surfaces. Scale bars: 5 nm. (f) The corresponding EDS mapping for Pb and Ag elements. The images reveal a scarcity of Pb element at the fusion site, as highlighted within the red rectangular area. This can be attributed to the lack of ligand coverage at the fusion interface and the presence of spatial environments unfavorable for ligand adsorption, such as grooves formed during the process. (g) HRTEM image of the NCs subjected to ligand exchange with (AgI+MAI+PbI_2). The absence of oriented bridging in the NCs confirms the importance of lattice matching ($MAPbI_3$: 6.31 Å vs. $AgBiS_2$: 5.69 Å). Scale bars: 20 nm.

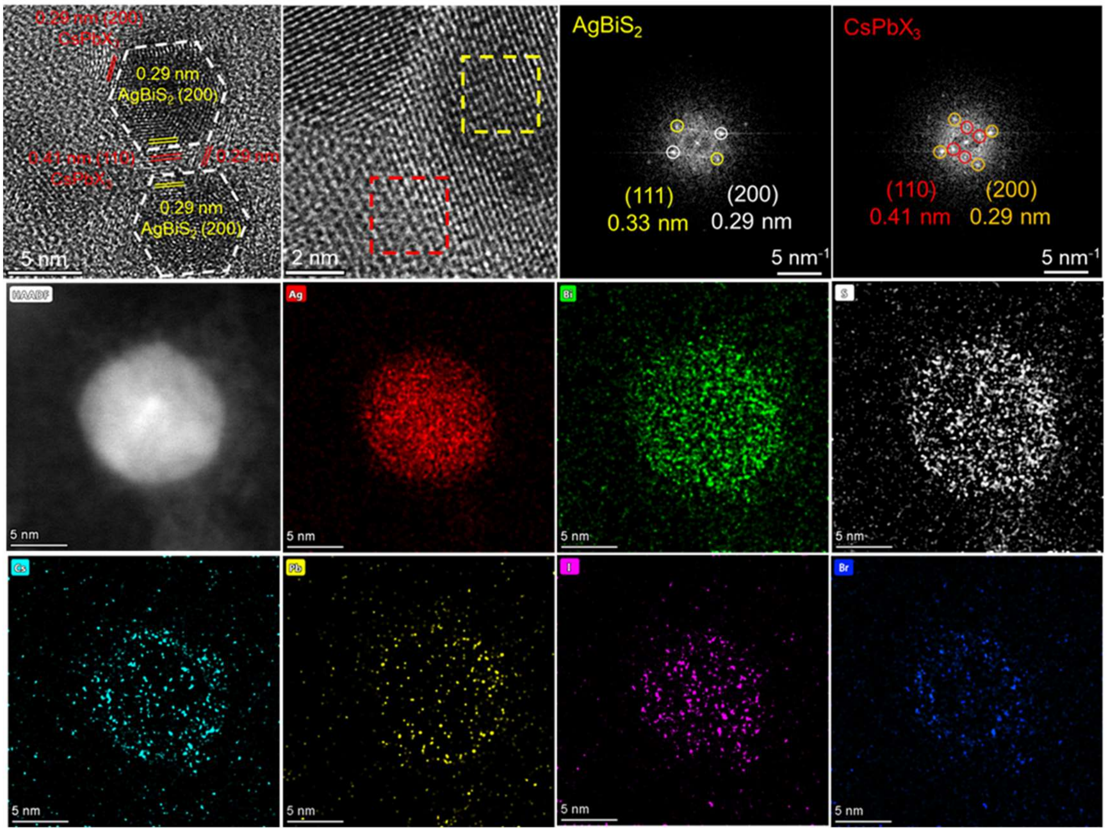

Supplementary Figure 14. HRTEM and high-resolution high-angle annular dark-field scanning transmission electron microscopy (HAADF-STEM) image of P-modified NCs without washing. Two different lattice fringes appeared between the NCs as indicated in the HRTEM image. Among these, the lattice spacing of 0.41 nm can be ascribed to the (110) facet of cubic $CsPbX_3$. The lattice spacing of 0.29 nm precisely corresponds to the (200) facet of cubic $CsPbX_3$. which supports the epitaxial growth of $CsPbX_3$ along the (200) facet of $AgBiS_2$ NCs. The elemental mapping localized Ag, Bi, and S signals to the NC cores, while Cs, Pb, and halides (I/Br) predominantly occupy peripheral regions. This spatial segregation confirms the $CsPbX_3$ epitaxial grows on the surface of $AgBiS_2$ NC cores. However, the excessively thick perovskite matrix is unable to effectively bridge the NCs.

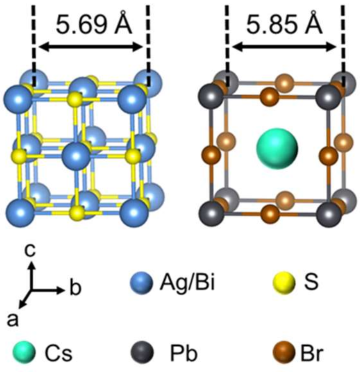

Supplementary Figure 15. (a) The crystal structures of $AgBiS_2$ and $CsPbBr_3$.

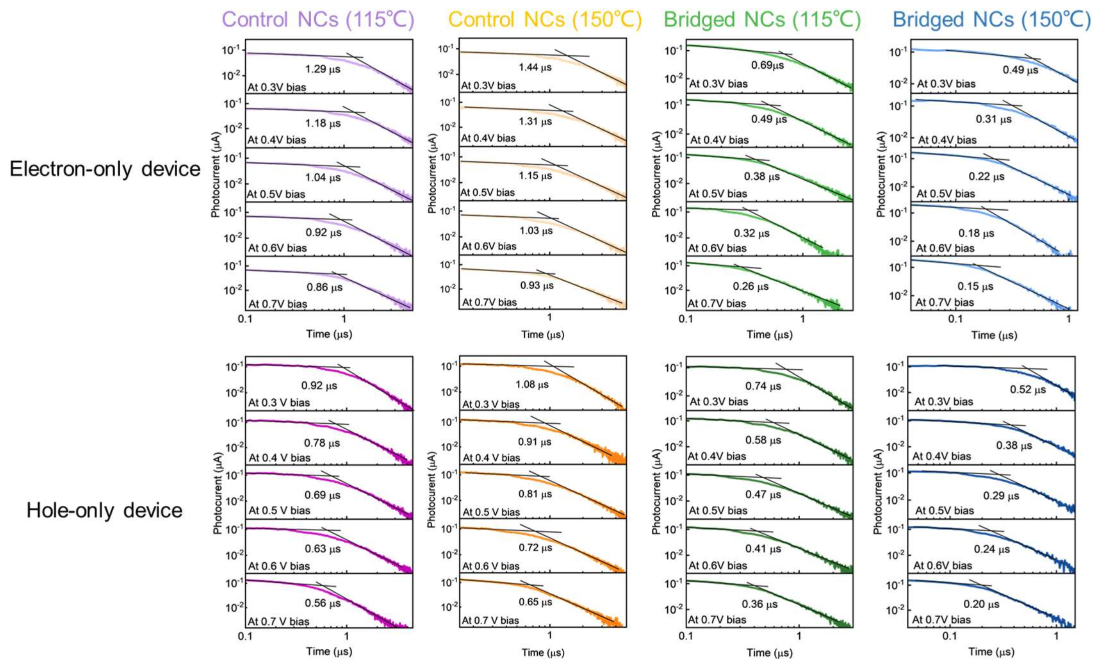

Supplementary Figure 16. Time-of-flight (TOF) analysis of electron- and hole-only devices for control NC and bridged NC films after annealing at 115 °C and 150 °C, respectively.

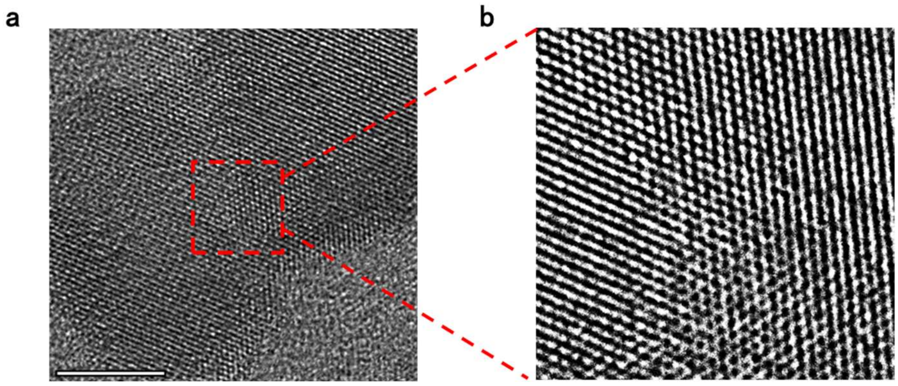

Supplementary Figure 17. HRTEM images of control NCs annealed at 150 °C. (a) HRTEM image shows two control NCs fusing together. Scale bars: 5 nm. (b) Inverse FFT image of the red-framed region in (a). Point defects can be seen in the boundary of NCs due to uncontrolled nanocrystals fusing.

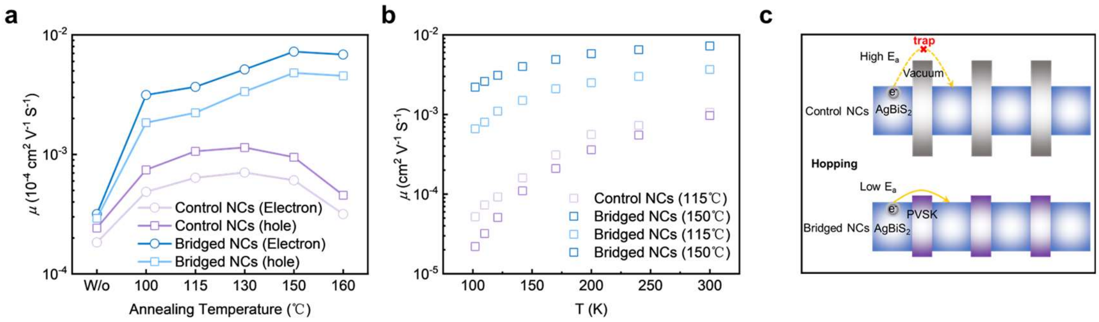

Supplementary Figure 18. (a) Carrier (electron and hole) mobility of control NC and bridged NC films obtained at different annealing temperatures. (b) Temperature-dependence of charge carrier mobilities for control NC (hole) and bridged NC (electron) films under 115 °C and 150 °C annealing, respectively. (c) Schematic diagrams of charge transport in the control NC and bridged NC films. The height of the potential barrier (activation energy) that needs to be overcome for hopping transport in the control NC films is significantly higher than that in the bridged NC films, making the transport process prone to being captured by defect states. Bridging reduces the spacing, which drastically lowers the tunneling barrier and activation energy. This, in turn, enhances transport efficiency despite the presence of Type-I band alignment.

Supplementary Note 2

Bridged NC films exhibited slightly higher mobility than control NCs in the unannealed state. It is noteworthy that after undergoing 100°C annealing, bridged NC films achieved a tenfold mobility enhancement relative to both the unannealed bridged NC film and the control NC film annealed at identical temperatures. This abrupt increase thus confirms the formation of perovskite molecular bridges at this temperature. As demonstrated in the relevant literature, an increase in annealing temperature resulted in a progressive enhancement of mobility in bridged NC films. [11] This phenomenon was attributed to an elevated level of cation disorder. The maximum observed effect was recorded at 150°C. The subsequent marginal decline at 160°C is likely attributable to thermally induced defects *via* cation exchange processes. In contrast, the control NC films showed an enhancement in mobility at temperatures ranging from 115 to 130°C, in comparison to the unannealed samples, followed by a decline in mobility. This non-

monotonic behavior can be attributed to the competition between two opposing mechanisms: (1) disorder-enhanced carrier transport and (2) defect trapping from NC fusing at high temperatures annealing (above 115°C). It can be concluded that the bridged NC films enable high temperature annealing to achieve greater cation disorder by virtue of the high thermal stability of the inorganic molecular perovskite layer between bridged NCs.

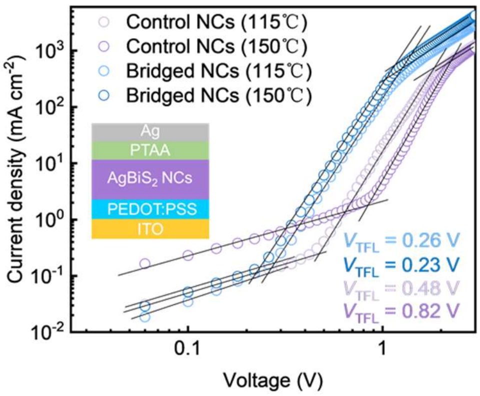

Supplementary Figure 19. The space charge limited conduction (SCLC) curves of hole-only devices fabricated with the control NC and bridged NC films under 115 °C and 150 °C annealing, respectively (from 0 to 4.0 V). The inset displays the schematic stack structure of the hole-only device.

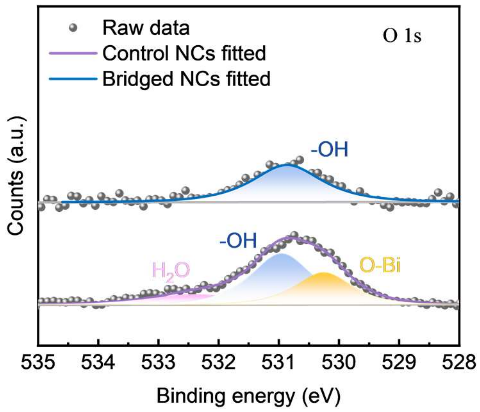

Supplementary Figure 20. High resolution XPS core-level O 1*s* spectra of the control NC and bridged NC films. XPS analysis of O 1*s* reveals that the oxygen content in the bridged NC films is significantly lower than that in the control NC films. Moreover, the O-Bi peak at low binding energy and the H_2O peak at high binding energy completely disappear,[11] confirming the efficient surface passivation of $AgBiS_2$ NCs by molecular perovskite layer epitaxial growth on (100) facets. This passivation significantly suppresses surface oxidation and detrimental group adsorption, thereby reducing the probability of carrier trapping.

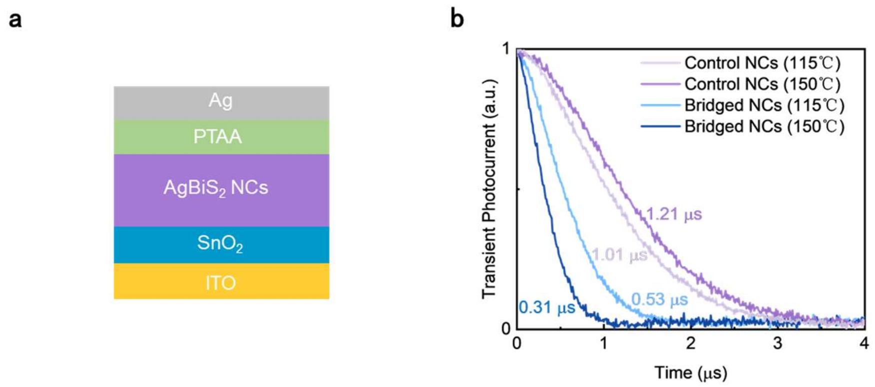

Supplementary Figure 21. (a) The structure of $AgBiS_2$ NC solar cells. (b) TPV measurements of control NC and bridged NC devices under 115 °C and 150 °C annealing, respectively.

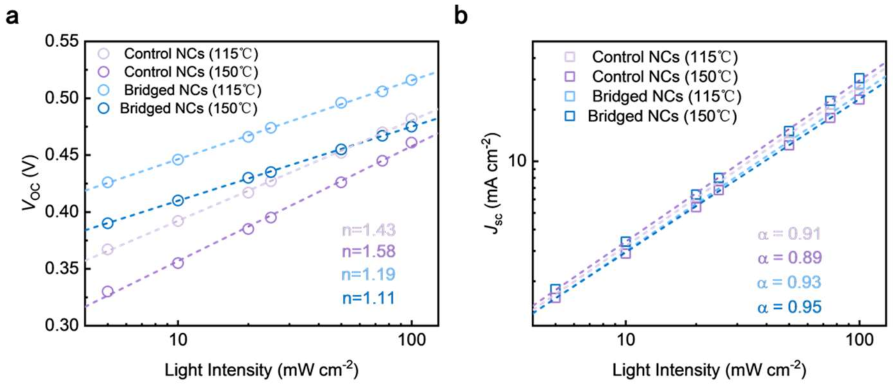

Supplementary Figure 22. Intensity-dependent (a) V_{OC} and (b) J_{SC} variation for the control NC and bridged NC devices under 115 °C and 150 °C annealing, respectively.

Supplementary Note 3

We initially fabricated solar cells and conducted transient photocurrent (TPC) and photovoltage (TPV) measurements, which provided further insight into the charge transport and recombination dynamics of the two types of NC films in solar cell devices. As shown in Fig. 4e, under 115 °C annealing, the bridged NC devices exhibited significantly prolonged TPV decay (2.02 μs *vs.* 0.95 μs), indicating suppressed charge recombination. Further increasing the annealing temperature to 150 °C, the bridged NC devices still showed a slight improvement in decay, which can be attributed to stronger crystallinity at this temperature effectively inhibiting defect state generation. In contrast, the TPV of control NC devices decayed rapidly at high temperature, indicating the generation of numerous defect states. As shown in Supplementary Fig. 21b, under 115 °C annealing, the bridged NC devices showed faster TPC decay (0.53 μs vs. 1.01 μs), demonstrating enhanced carrier extraction efficiency. When the annealing temperature was further increased to 150 °C, the TPC decay of bridged NC devices was further accelerated, indicating a further improvement in extraction efficiency, while that of control NC devices decreased. This was further supported by reduced ideality factor (n) in bridged NC devices from light intensity-dependent V_{OC} analysis within the range of 0.05 to 1 sun intensity (Supplementary Fig. 22a), approaching the theoretical limit of n = 1 for minimal trap-assisted recombination. In addition, we used the power-law

expression $J_{SC} \propto I^{\alpha}$ to fit the relationship between J_{SC} and light intensity, where I represents the light intensity and α is the power factor (Supplementary Fig. 22b). Higher α values confirmed the enhanced charge extraction capability of bridged NCs.

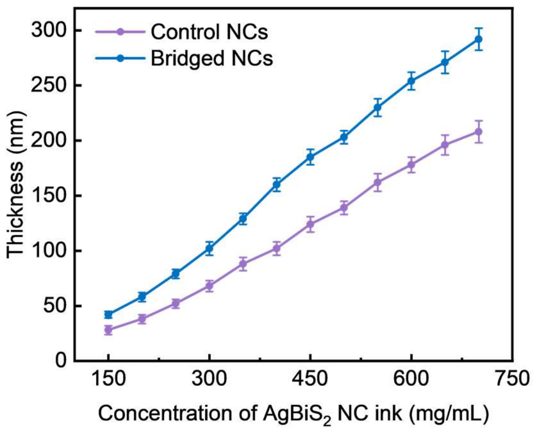

Supplementary Figure 23. Thickness of control NC and bridged NC films according to NC ink concentration, respectively. The estimated thicknesses are listed in Table S4. The bridged NC films consistently had a thicker thickness than the control NC films at the same concentrations, and a steep increase in thickness was observed in the bridged NC films as the NC concentration increased. In contrast, the control NC films displayed a much more gradual thickness-concentration dependence. This disparity can be attributed to the inferior colloidal dispersibility of the control NCs ink (Supplementary Fig. 8), which typically limits the film thickness due to aggregation effects.[12]

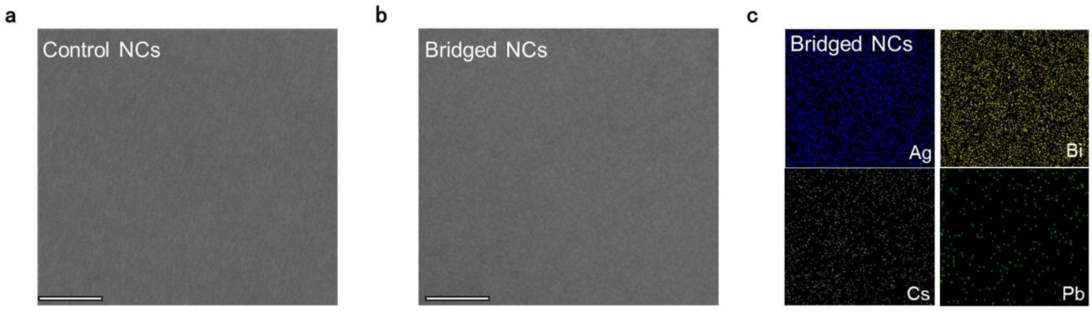

Supplementary Figure 24. SEM image of (a) control NCs and (b) bridged NCs film. Scale bar: 500 nm. Both the ~40 nm-thick control NCs film and ~185 nm bridged NCs film are relatively flat and smooth. (c) Elemental mappings of Ag, Bi, Cs and Pb in the scanned area of bridged NCs films. Perovskite elements (Cs, Pb) are uniformly dispersed in the bridged $AgBiS_2$ NCs film.

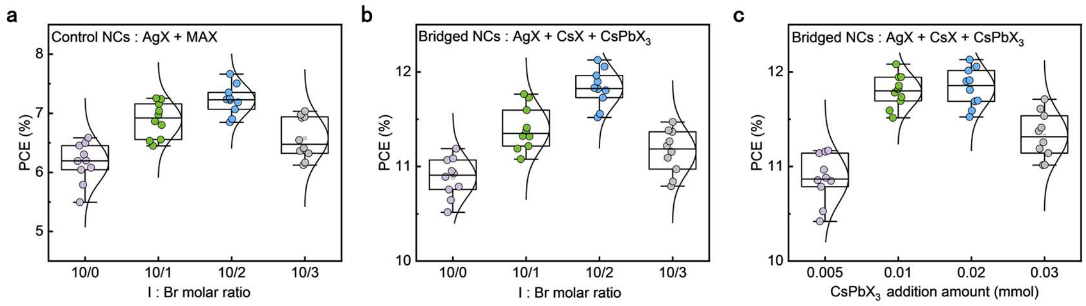

Supplementary Figure 25. Optimization of halide composition in ligand systems for (a) control and (b) bridged NCs, with optimal AgI: AgBr ratio of 5:1 for both systems. The perovskite component in the bridged NCs ligand system was fixed at 0.02 mmol CsBr + 0.02 mmol $PbBr_2$. (c) Optimization of perovskite component dosage in bridged NCs, showing optimal performance at 0.02 mmol $CsPbBr_3$ equivalent.

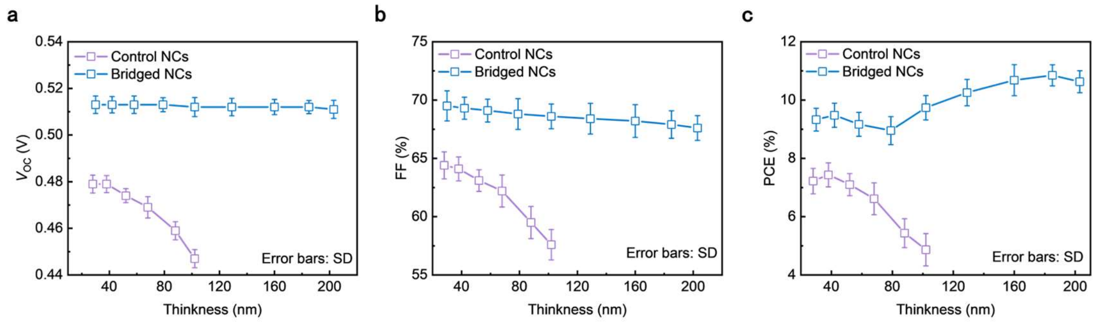

Supplementary Figure 26. Device performances of the control NC and bridged NC devices with different active layer thicknesses, including (a) V_{OC}, (b) FF, and (c) PCE. Fixed the annealing temperature at 115 °C. The average values are taken from fifteen devices in each group.

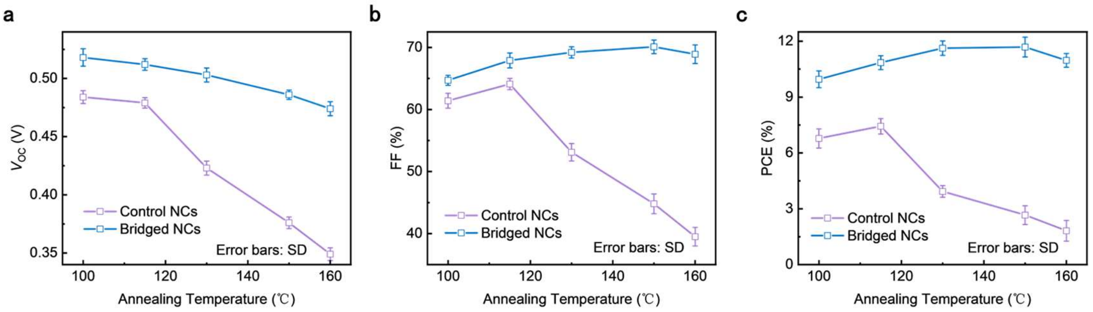

Supplementary Figure 27. Device performances of the control NC and bridged NC devices with annealing under different temperatures, including (a) V_{OC}, (b) FF, and (c) PCE. fixed the photoactive layer thickness of the control NC devices and bridged NC devices at ~40 nm and ~185 nm, respectively. The average values are derived from fifteen devices in each group.

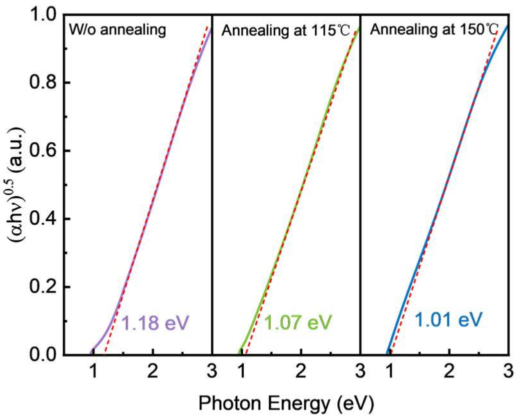

Supplementary Figure 28. The band gaps of bridged NC films at different annealing temperatures fitted by the Tauc plot. As the annealing temperature increases, the band gap of NCs gradually decreases, a phenomenon attributed to band gap narrowing caused by cationic disorder.[13]

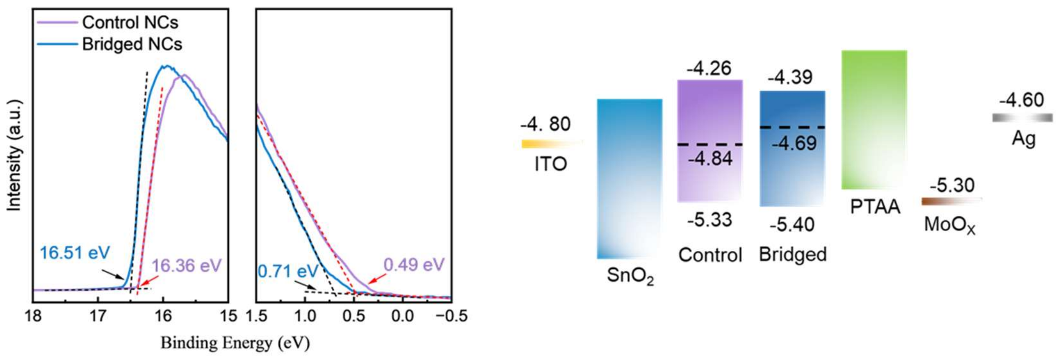

Supplementary Figure 29. Ultraviolet photoelectron spectroscopy (UPS) of the control NC (annealing at 115°C) and bridged NC films (annealing at 150°C), and the corresponding energy band diagrams obtained by combining the bandgaps determined from Tauc analysis (Supplementary Fig. 28).[1]

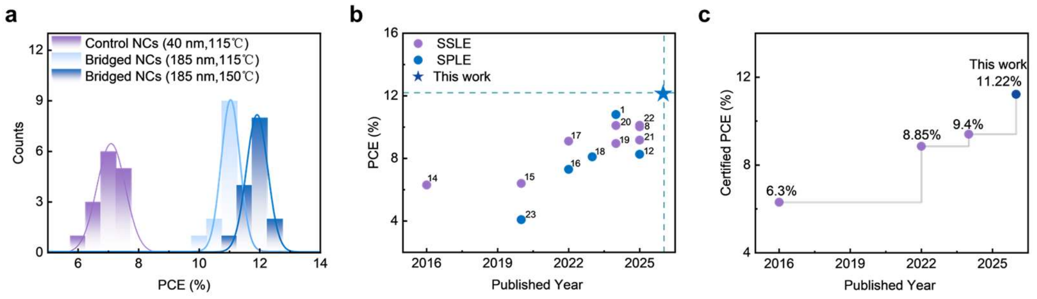

Supplementary Figure 30. (a) Statistical Histogram of PCE collected from each of 15 different control NC devices (~40 nm, annealed at 115°C) and bridged NC devices (~185 nm, annealed at 115°C or 150°C). (b) An overview of PCE evolution in $AgBiS_2$ NCs solar cells categorized by ligand-exchange methods. The corresponding values and references are listed in Supplementary Table 11 and in the brackets at the upper right corner, respectively. (c) An overview of certified PCE evolution in $AgBiS_2$ NCs solar cells.

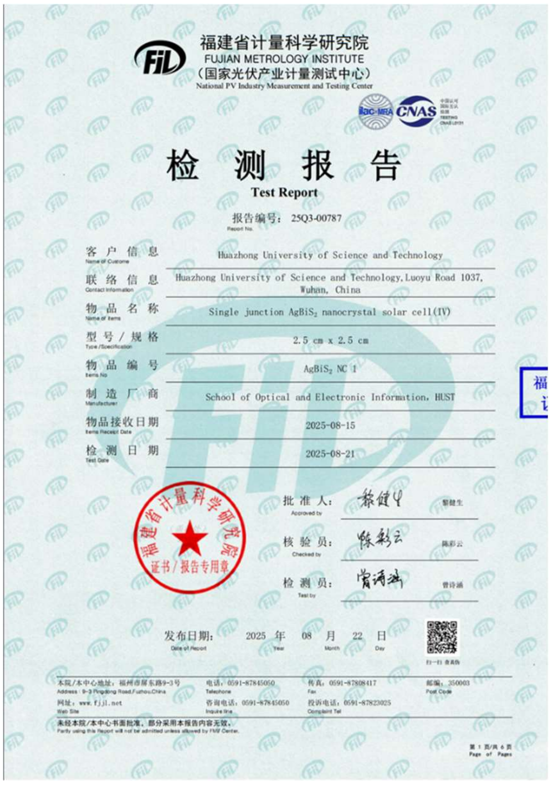

福建省计量科学研究院
FUJIAN METROLOGY INSTITUTE
（国家光伏产业计量测试中心）
National PV Industry Measurement and Testing Center

检　测　报　告
Test Report

报告编号：25Q3-00787
Report No.

客户信息 Name of Custome	Huazhong University of Science and Technology
联络信息 Contact Information	Huazhong University of Science and Technology, Luoyu Road 1037, Wuhan, China
物品名称 Name of Items	Single junction $AgBiS_2$ nanocrystal solar cell(IV)
型号/规格 Type /Specification	2.5 cm x 2.5 cm
物品编号 Items No	$AgBiS_2$ NC 1
制造厂商 Manufacturer	School of Optical and Electronic Information, HUST
物品接收日期 Items Receipt Date	2025-08-15
检测日期 Test Date	2025-08-21

福建省计量科学研究院 证书/报告专用章

批准人：Approved by 黎健生
核验员：Checked by 陈彩云
检测员：Test by 曾诗涵

发布日期：Date of Report 2025 年 Year 08 月 Month 22 日 Day

扫一扫 查真伪

本院/本中心地址：福州市屏东路9-3号 Address: 9-3 Pingdong Road,Fuzhou,China
电话：0591-87845050 Telephone
传真：0591-87808417 Fax
邮编：350003 Post Code
网址：www.fjjl.net Web Site
咨询电话：0591-87845050 Inquire line
投诉电话：0591-87823025 Complaint Tel

未经本院/本中心书面批准，部分采用本报告内容无效。
Partly using this Report will not be admitted unless allowed by FMIF Center.

第 1 页/共 6 页 Page of Pages

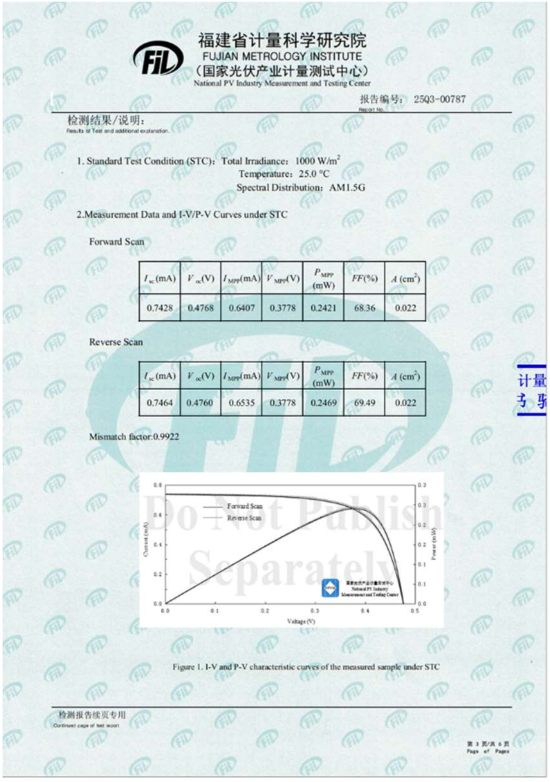

福建省计量科学研究院
FUJIAN METROLOGY INSTITUTE
（国家光伏产业计量测试中心）
National PV Industry Measurement and Testing Center

报告编号：25Q3-00787
Report No.

检测结果/说明：
Results of Test and additional explanation.

1. Standard Test Condition (STC): Total Irradiance: 1000 W/m^2
Temperature: 25.0 °C
Spectral Distribution: AM1.5G

2.Measurement Data and I-V/P-V Curves under STC

Forward Scan

I_{sc} (mA)	V_{oc}(V)	I_{MPP}(mA)	V_{MPP}(V)	P_{MPP} (mW)	FF(%)	A (cm^2)
0.7428	0.4768	0.6407	0.3778	0.2421	68.36	0.022

Reverse Scan

I_{sc} (mA)	V_{oc}(V)	I_{MPP}(mA)	V_{MPP}(V)	P_{MPP} (mW)	FF(%)	A (cm^2)
0.7464	0.4760	0.6535	0.3778	0.2469	69.49	0.022

Mismatch factor:0.9922

Figure 1. I-V and P-V characteristic curves of the measured sample under STC

检测报告续页专用
Continued page of test report

第 3 页/共 6 页 Page of Pages

Supplementary Figure 31. Photovoltaic Cell Performance Certificate for bridged $AgBiS_2$ NCs solar cell from the National PV Industry Measurement and Testing Center.

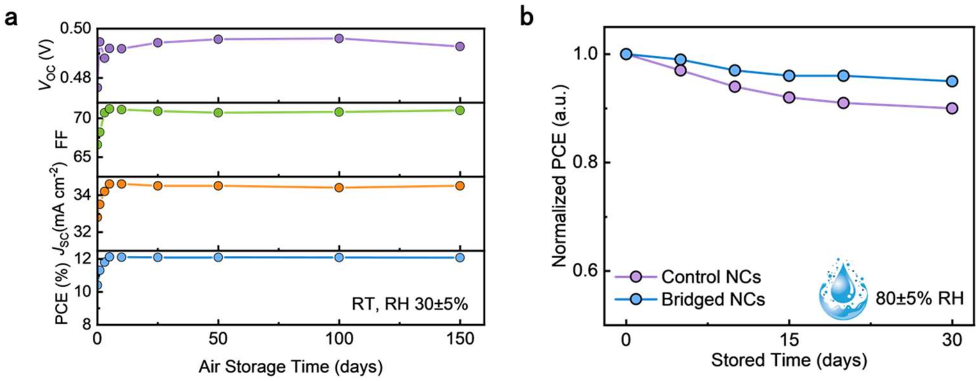

Supplementary Figure 32. (a) Air stability of the bridged NC devices. The devices were stored unencapsulated in ambient air at 25–35% relative humidity (RH) and room temperature for 150 days. (b) Excellent retention of bridged NCs device performance after one-month storage at high humidity (80 ± 5% RH), attributable to the stable, epitaxially anchored molecular perovskite bridge that effectively impedes moisture ingress.

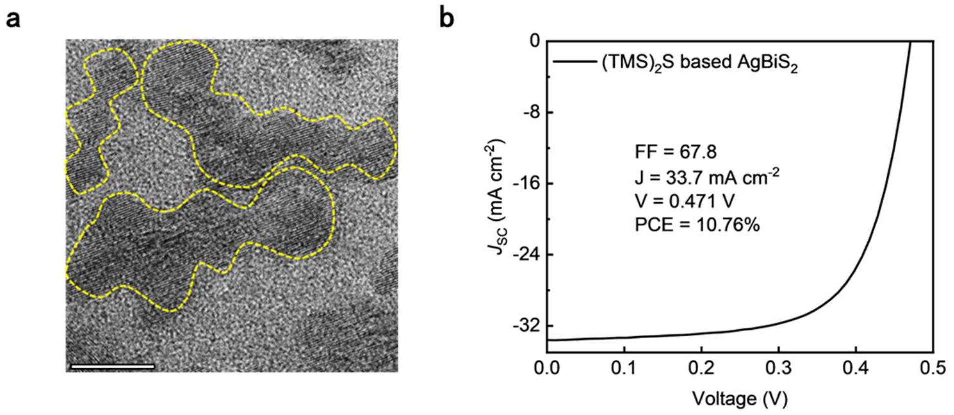

Supplementary Figure 33. (a) HRTEM image of $AgBiS_2$ NCs prepared based on $(TMS)_2S$ after perovskite bridging. TEM analysis shows that these NCs with a wide size distribution can also be bridged, but their morphology is relatively disordered and less uniform compared to those based on S-OLA. Scale bar: 5 nm. (b) *J-V* curves of devices based on $(TMS)_2S$-prepared $AgBiS_2$ NCs after perovskite bridging.

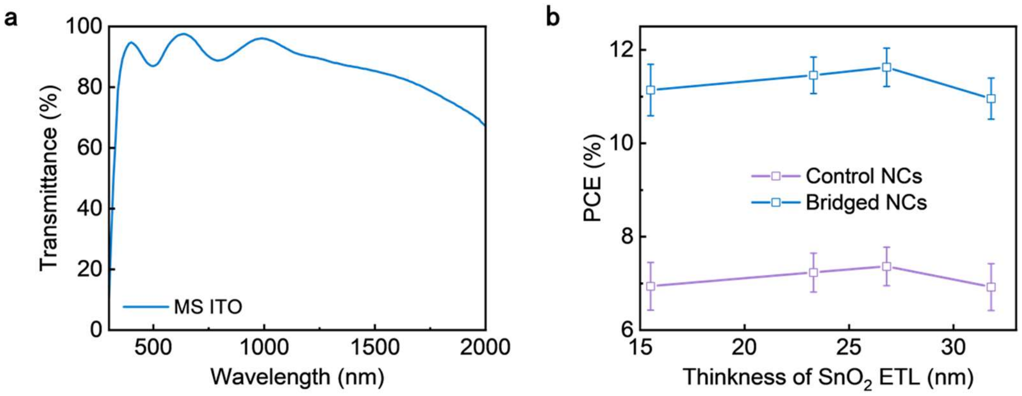

Supplementary Figure 34. (a) The transmittance curves of optimal condition MS ITO films. (b) The performance of devices fabricated with different SnO_2 thicknesses was compared, with the results showing optimal performance at approximately 26 nm. The thickness was measured by atomic force microscopy.

Supplementary Table 1. The adsorption energies (E_{ads}) of $[AgI_2]^-$, $[AgBr_2]^-$, $[PbI_3]^-$, $[PbBr_3]^-$, $[PbI_4]^{2-}$ and $[PbBr_4]^{2-}$ ligands onto (100) and (111) surfaces, respectively.

Ligands / E_{ads}(eV)	AgI_2^-	$AgBr_2^-$	PbI_3^-	$PbBr_3^-$	PbI_4^{2-}	$PbBr_4^{2-}$
(111) facet	-3.72	-3.84	-3.89	-3.95	-4.48	-4.62
(100) facet	-1.82	-2.15	-2.18	-2.34	-2.84	-3.02

Supplementary Table 2. Relative amounts of each element found by XPS for the $AgBiS_2$-OA/OLA NC films, control NC films, P-modified films without washing and P-modified films with washing. The atomic ratio is normalized by the composition of the Bi element [a].

Elements	$AgBiS_2$-OA/OLA	Control	P-modified (W/o washing)	P-modified (Washing 1 time)	P-modified (Washing 2 times)
Ag	1.46	1.98	1.97	1.95	1.98
S	1.71	1.75	1.75	1.77	1.76
O	1.32	0.26	0.08	0.07	0.07
I	-	1.12	1.23	1.16	1.16
Br	-	0.29	0.42	0.35	0.34
Pb	-	-	0.12	0.01	0.01
Cs	-	-	0.91	0.74	0.75
Pb content (ppm)	-	0	34830	2752	2740

[a] Quantities normalized to Bi.

Supplementary Table 3. Relative amount of Pb measured using ICP-OES in control NCs sample, P-modified sample without washing and P-modified sample with washing.

	P-modified (W/o washing)	P-modified (Washing 1 time)	P-modified (Washing 2 times)
Sample mass/g	0.00389	0.00618	0.00618
Constant volume/ml	15	15	15
Dilution factor	1	1	1
Tested element	Pb	Pb	Pb
Instrument reading (ug/L)	7600	315	312
Calculated content (ppm)	29430	761	754

Supplementary Table 4. TOF analysis of electron- and hole-only devices of control NC and bridged NC films after annealing at 115 °C and 150 °C, respectively.

Control NCs films (115 °C)

bias (V)	L^2/τ_{Elec}	L^2/τ_{Hole}	τ_{Elec}	τ_{Hole}
0.3	4.84	6.79	1.29	0.92
0.4	5.29	8.01	1.18	0.78
0.5	6.01	9.06	1.04	0.69
0.6	6.79	9.92	0.92	0.63
0.7	7.27	11.16	0.86	0.56

Control NCs films (150 °C)

bias (V)	L^2/τ_{Elec}	L^2/τ_{Hole}	τ_{Elec}	τ_{Hole}
0.3	4.34	5.79	1.44	1.08
0.4	4.77	6.87	1.31	0.91
0.5	5.43	7.72	1.15	0.81
0.6	6.07	8.68	1.03	0.72
0.7	6.72	9.62	0.93	0.65

Bridge NCs films (115°C)

bias (V)	L^2/τ_{Elec}	L^2/τ_{Hole}	τ_{Elec}	τ_{Hole}
0.3	8.5	9.07	0.74	0.69
0.4	10.8	12.8	0.58	0.49
0.5	13.2	16.3	0.47	0.38
0.6	15.3	20.1	0.41	0.32
0.7	17.6	23.7	0.36	0.26

Bridge NCs films (150°C)

bias (V)	L^2/τ_{Elec}	L^2/τ_{Hole}	τ_{Elec}	τ_{Hole}
0.3	12.02	12.76	0.52	0.49
0.4	16.45	20.16	0.38	0.31

0.5	21.55	28.41	0.29	0.22
0.6	26.04	34.72	0.24	0.18
0.7	31.25	41.67	0.20	0.15

Supplementary Table 5. The corresponding carrier mobilities obtained from TOF analysis of electron-only and hole-only devices of control NC and bridged NC films annealed at 115 °C and 150 °C, respectively.

	μ_e [cm^2 V^{-1} s^{-1}]	μ_h [cm^2 V^{-1} s^{-1}]
Control NCs (115 °C)	6.36×10^{-4}	1.06×10^{-3}
Control NCs (150 °C)	6.06×10^{-4}	9.47×10^{-4}
Bridged NCs (115 °C)	3.67×10^{-3}	2.23×10^{-3}
Bridged NCs (150 °C)	7.24×10^{-3}	4.81×10^{-3}

Supplementary Table 6. Trap-filled limit voltage (V_{TFL}), trap density (N_{trap}), and electron mobility (μ_e) are derived from the SCLC curves of the electron-only devices of control NC and bridged NC films annealed at 115 °C and 150 °C, respectively.

	V_{TFL} [V]	N_{trap} [cm^{-3}]	μ_e [cm^2 V^{-1} s^{-1}]
Control NCs (115°C)	0.66	6.95×10^{17}	5.42×10^{-4}
Control NCs (150°C)	0.94	9.89×10^{17}	4.89×10^{-4}
Bridged NCs (115°C)	0.17	1.79×10^{17}	3.25×10^{-3}
Bridged NCs (150°C)	0.12	1.26×10^{17}	6.86×10^{-3}

Supplementary Table 7. V_{TFL}, N_{trap}, and hole mobility (μ_h) are derived from the SCLC curves of the hole-only devices of control NC and bridged NC films annealed at 115 °C and 150 °C, respectively.

	V_{TFL} [V]	N_{trap} [cm^{-3}]	μ_h [cm^2 V^{-1} s^{-1}]
Control NCs (115°C)	0.48	5.04×10^{17}	9.36×10^{-4}
Control NCs (150°C)	0.82	8.61×10^{17}	8.94×10^{-4}
Bridged NCs (115°C)	0.26	2.72×10^{17}	1.89×10^{-3}
Bridged NCs (150°C)	0.23	2.41×10^{17}	4.24×10^{-3}

Supplementary Table 8. The calculated carrier (electron and hole) diffusion length of control NC and bridged NC films after annealing 115 °C and 150 °C.

Carriers diffusion length (nm)	Electron	Hole
Control NCs (115°C)	39.5	50.9
Control NCs (150°C)	31.1	39.7
Bridged NCs (115°C)	138.7	108.1
Bridged NCs (150°C)	204.3	166.5

Supplementary Table 9. The thicknesses (T) of the control NC and bridged NC films estimated by a profilometer at different concentrations (C) of the NCs inks.

C (mg mL^{-1}) / T (nm)	150	200	250	300	350	400	450	500	550	600	650	700
Control NCs	28	38	52	68	88	102	124	139	162	178	196	208
Bridged NCs	42	58	79	102	129	160	185	203	230	254	271	292

Supplementary Table 10. Photovoltaic performance of $AgBiS_2$ solar cells fabricated with different ligand combinations [a].

Ligands ensemble	AgX+MAX	AgX+CsX	AgX+PbX_2
J_{SC} (mA cm^{-2})	24.7 (24.2 ± 0.6)	23.2 (22.8 ± 0.5)	22.6 (21.7 ± 0.7)
V_{OC} (V)	0.482 (0.479 ± 0.004)	0.457 (0.448 ± 0.006)	0.445 (0.439 ± 0.005)
FF (%)	65.3 (64.1 ± 0.9)	61.2 (60.3 ± 0.8)	60.2 (59.6 ± 0.7)
PCE (%)	7.77 (7.43 ± 0.41)	6.49 (6.06 ± 0.47)	6.05 (5.58 ± 0.49)

[a] Values in parentheses indicate average and standard deviation of each parameter collected from 15 devices.

Supplementary Table 11. A summary of reported device parameters for $AgBiS_2$ NCs solar cells, categorized based on ligand-exchange methods utilized in the device fabrication.

Ligand exchange method	PCE (%)	Year	Ref.
Solid-state	6.3 (certified 6.3%)	2016	[14]
	6.4	2020	[15]
	9.1 (certified 8.85%)	2022	[17]
	8.95	2024	[19]
	10.1	2024	[20]
	9.17	2025	[21]
	10.02	2025	[8]
	10.11	2025	[22]
Solution-phase	4.08	2020	[23]
	7.3	2022	[16]
	8.1	2023	[18]
	10.84 (certified 9.4%)	2024	[1]
	8.26	2025	[12]
	12.13 (certified 11.22%)	2026	This work

Supplementary Reference

[1] Oh, J. T. et al. Post-deposition in situ passivation of $AgBiS_2$ nanocrystal inks for high-efficiency ultra-thin solar cells. *Energy Environ. Sci*. **17**, 8885-8892 (2024).

[2] Blöchl, P.E. Projector augmented-wave method. *Phys. Rev. B* **50**, 17953 (1994).

[3] Kresse, G. et al. Efficiency of ab-initio total energy calculations for metals and semiconductors using a plane-wave basis set**.** *J. Comput. Mater. Sci.* **6**, 15 (1996).

[4] Momma, K. et al. *VESTA* 3 for three-dimensional visualization of crystal, volumetric and morphology data. *J. Appl. Crystallogr.* **44**, 1272 (2011).

[5] Chen, D. et al. Passivating {100} facets of PbS colloidal quantum dots via perovskite bridges for sensitive and stable infrared photodiodes. *Adv. Funct. Mater.* **33**, 2210158 (2023).

[6] Lowry, G. et al. Guidance to improve the scientific value of zeta potential measurements in nano-EHS. *Environ. Sci. Nano* **3**, 953-965 (2016).

[7] Ding, C. et al. Over 15% efficiency PbS quantum-dot solar cells by synergistic effects of three interface engineering: reducing nonradiative recombination and balancing charge carrier extraction. *Adv. Energy Mater.* **12**, 2201676 (2022).

[8] Yang, W. et al. Boosting Open-Circuit Voltage of $AgBiS_2$ Quantum Dot Solar Cells through Post-treatment Passivation. *ACS Energy Lett.* **10**, 58-67 (2025).

[9] Wang, J. et al. 21.15%-Efficiency and Stable $CsPbI_3$ Perovskite Solar Cells Enabled by an Acyloin Ligand. *Adv. Mater.* 2210223 (2025).

[10] Zhou, Q. et al. Tailored Lattice "Tape" to Confine Tensile Interface for 11.08%-Efficiency All-Inorganic $CsPbBr_3$ Perovskite Solar Cell with an Ultrahigh Voltage of 1.702 V. *Adv. Sci.* **8**, 2101418 (2021).

[11] Chen, J. et al. Ligand-Tuned $AgBiS_2$ Planar Heterojunctions Enable Efficient Ultrathin Solar Cells. *ACS Nano* **18**, 49, 33348–33358 (2024).

[12] Kim, H. J. et al. Homogeneously Blended Donor and Acceptor $AgBiS_2$ Nanocrystal Inks Enable High-Performance Eco-Friendly Solar Cells with Enhanced Carrier Diffusion Length. *Adv. Energy Mater.* 2404552 (2025).

[13] Viñes, F. et al. Bandgap engineering by cationic disorder: case study on AgBiS2. *Phys. Chem. Chem. Phys.* **19**, 27940-27944 (2017).
[14] Bernechea, M. et al. Solution-processed solar cells based on environmentally friendly $AgBiS_2$ nanocrystals. *Nat. Photonics* **10**, 521 (2016).
[15] Burgués-Ceballos, I. et al. Colloidal $AgBiS_2$ nanocrystals with reduced recombination yield 6.4% power conversion efficiency in solution-processed solar cells. *Nano Energy* **75**, 104961 (2020).
[16] Wang, Y. et al. Environmentally Friendly $AgBiS_2$ Nanocrystal Inks for Efficient Solar Cells Employing Green Solvent Processing. *Adv. Energy Mater.* **12**, 2200700 (2022).
[17] Wang, Y. et al. Cation disorder engineering yields $AgBiS_2$ nanocrystals with enhanced optical absorption for efficient ultrathin solar cells. *Nat. Photonics* **16**, 235 (2022).
[18] Kim, D. et al. Multi-Facet Passivation of Ternary Colloidal Quantum Dot Enabled by Quadruple-Ligand Ensemble toward Efficient Lead-Free Optoelectronics. *Adv. Energy Mater.* **14**, 2302579 (2023).
[19] Zhong, Q. et al. Solvent-Engineering-Assisted Ligand Exchange Strategy for High-Efficiency $AgBiS_2$ Quantum Dot Solar Cells. Angew. Chem. Int. Ed. 63.52: e202412590 (2024).
[20] Lee, J. et al. High Efficiency (>10%) $AgBiS_2$ Colloidal Nanocrystal Solar Cells with Diketopyrrolopyrrole-Based Polymer Hole Transport Layer. *Adv. Mater.* **37**, 2413081 (2025).
[21] Yuan, L. et al. Direct Synthesis of Semiconductive $AgBiS_2$ NC Inks toward High-Efficiency, Low-Cost and Environmental-Friendly Solar Cells. *Angew. Chem. Int. Ed.* **64**, e202416369 (2025).
[22] Liu, Y. et al. Vacancy-Catalyzed Cation Homogenization for High-Performance $AgBiS_2$ Nanocrystal Solar Cells. *ACS Energy Lett.* **10**, 2068-2074 (2025).
[23] Bae, S. et al. Improved Eco-Friendly Photovoltaics Based on Stabilized $AgBiS_2$ Nanocrystal Inks. *Chem. Mater.* **32**, 10007 (2020).
