# Supplementary material for: Molecular lead halide perovskite layer bridged AgBiS2 nanocrystals for efficient thin film solar cells
Source: Nat Commun. 2026 Apr 24;17:5687. doi: 10.1038/s41467-026-72272-4 (PMC13319432; doi:10.1038/s41467-026-72272-4)
Supplement: Supplementary file 2 — Reporting Summary [file 41467_2026_72272_MOESM2_ESM.pdf]

nature portfolio

Corresponding author(s): Mingkui Wang

Solar Cells Reporting Summary

Nature Portfolio wishes to improve the reproducibility of the work that we publish. This form is intended for publication with all accepted papers reporting the characterization of photovoltaic devices and provides structure for consistency and transparency in reporting. Some list items might not apply to an individual manuscript, but all fields must be completed for clarity.

For further information on Nature Research policies, including our data availability policy, see Authors & Referees.

Experimental design

Please check the following details are reported in the manuscript, and provide a brief description or explanation where applicable.

1. Dimensions

Area of the tested solar cells — [x] Yes [ ] No
aperture area: 0.022 cm-2
Explain why this information is not reported/not relevant.

Method used to determine the device area — [x] Yes [ ] No
Automatic image measuring instrument
Explain why this information is not reported/not relevant.

2. Current-voltage characterization

Current density-voltage (J-V) plots in both forward and backward direction — [x] Yes [ ] No
Figure 5f and Figure S29

Voltage scan conditions — [x] Yes [ ] No
Scan direction: forward and backward
Explain why this information is not reported/not relevant.

Test environment — [x] Yes [ ] No
Temperature: 27.4°C RH:38%
Explain why this information is not reported/not relevant.

Protocol for preconditioning of the device before its characterization — [x] Yes [ ] No
Prior to the certification test, it was placed in a vacuum chamber with no additional preconditioning.
Explain why this information is not reported/not relevant.

Stability of the J-V characteristic — [x] Yes [ ] No
Maximum power point tracking test under continuous AM1.5G illumination for 500 min in ambient air and room temperature .
Explain why this information is not reported/not relevant.

3. Hysteresis or any other unusual behaviour

Description of the unusual behaviour observed during the characterization — [x] Yes [ ] No
With negligible hysteresis
Explain why this information is not reported/not relevant.

Related experimental data — [x] Yes [ ] No
In the certification efficiency test, the efficiency value for the forward scan was 11%, and that for the reverse scan was 11.22%. The hysteresis (of the J-V curve) was as low as 1.9%.
Explain why this information is not reported/not relevant.

4. Efficiency

External quantum efficiency (EQE) or incident photons to current efficiency (IPCE) — [x] Yes [ ] No
The integrated JSC values derived from the EQE spectra (Figure 5g) shows excellent agreement with those extracted from the J-V curves, with discrepancies below 3%
Explain why this information is not reported/not relevant.

April 2023

A comparison between the integrated response under the standard reference spectrum and the response measure under the simulator	☒ Yes ☐ No	During our certification process, two types of spectra were calibrated, with a calibration factor of 0.9922. *Explain why this information is not reported/not relevant.*
For tandem solar cells, the bias illumination and bias voltage used for each subcell	☐ Yes ☒ No	*Provide a description of the measurement conditions.* this is not about tandem cells

5. Calibration

Light source and reference cell or sensor used for the characterization	☒ Yes ☐ No	Solar Simulator: Spectral Match (300-1200) nm: Urel=8.0% (k =2); Irradiance Ratio: U =1.2% (k =2), Fujian Metrology Institute.<br>WPVS Reference Solar Cell: Urel=1.3% (k =2), National Institute of Metrology, China.<br>Automatic Image Measuring Instrument: MPE: ±(2.5+L /300)um, Fujian Metrology Institute. *Explain why this information is not reported/not relevant.*
Confirmation that the reference cell was calibrated and certified	☒ Yes ☐ No	Fujian Metrology Institute is a national statutory institution, and all its measuring instruments comply with relevant standards. *Explain why this information is not reported/not relevant.*
Calculation of spectral mismatch between the reference cell and the devices under test	☒ Yes ☐ No	During our certification process, two types of spectra were calibrated, with a calibration factor of 0.9922. *Explain why this information is not reported/not relevant.*

6. Mask/aperture

Size of the mask/aperture used during testing	☒ Yes ☐ No	aperture area: 0.022 cm^2 *Explain why this information is not reported/not relevant.*
Variation of the measured short-circuit current density with the mask/aperture area	☒ Yes ☐ No	When the area of the mask is less than 0.022 cm^2, the current decreases. When the area exceeds 0.022 cm^2, the current increases slightly. *Explain why this information is not reported/not relevant.*

7. Performance certification

Identity of the independent certification laboratory that confirmed the photovoltaic performance	☒ Yes ☐ No	National PV Industry Measurement and Testing Center (Fujian Metrology Institute) *Explain why this information is not reported/not relevant.*
A copy of any certificate(s)	☒ Yes ☐ No	Figure S29 *Explain why this information is not reported/not relevant.*

8. Statistics

Number of solar cells tested	☒ Yes ☐ No	1 *Explain why this information is not reported/not relevant.*
Statistical analysis of the device performance	☒ Yes ☐ No	Relevant information can be found in the description of Figure 5f in the main text. *Explain why this information is not reported/not relevant.*

9. Long-term stability analysis

Type of analysis, bias conditions and environmental conditions	☒ Yes ☐ No	The unencapsulation device exhibited 99.7% retention of its initial PCE after a 150-day storage period at 25-35% relative humidity and room temperature (Figure S30) *Explain why this information is not reported/not relevant.*
