# Supplementary material for: Molecular lead halide perovskite layer bridged AgBiS2 nanocrystals for efficient thin film solar cells
Source: Nat Commun. 2026 Apr 24;17:5687. doi: 10.1038/s41467-026-72272-4 (PMC13319432; doi:10.1038/s41467-026-72272-4)
Supplement: Supplementary file 3 — Transparent Peer Review file [file 41467_2026_72272_MOESM3_ESM.pdf]

nature portfolio

Peer Review File

Molecular Lead Halide Perovskite Layer Bridged AgBiS2 Nanocrystals for Efficient Thin Film Solar Cells

Corresponding Author: Professor Mingkui Wang

This manuscript has been previously reviewed at another journal. This document only contains information relating to versions considered at Nature Communications.

This file contains all reviewer reports in order by version, followed by all author rebuttals in order by version.

Version 0:

Reviewer comments:

Reviewer #1

(Remarks to the Author)
Most of the comments previously raised in the Nature Photonics review have been satisfactorily addressed, and I consider the current version suitable for publication in Nature Communications without further revision.

Reviewer #2

(Remarks to the Author)
The authors have thoroughly addressed the comments raised by the reviewers in the previous round, and the overall quality of the manuscript has been significantly improved. In particular, the authors have achieved the highest certified efficiency for $AgBiS_2$ nanocrystal solar cells. However, the so-called "Monolayer Perovskite Bridged" structure and its claimed remarkable effect remain unconvincing based on the current data and explanations provided in the manuscript. The authors introduced the heavy metal Pb into the environmentally friendly $AgBiS_2$ NC system. Although the authors claim that the amount is minimal, this still greatly undermines the environmental advantages of the $AgBiS_2$ NCs. Moreover, regarding novelty, the strategy and interpretations presented in this work closely follow those reported for PbS QD systems (doi.org/10.1016/j.joule.2020.05.011), and therefore lack sufficient originality. In summary, although this work achieves high device performance, substantial revisions are still required before it can be considered for publication in Nature Communications.

1. The authors claim that the NCs in Figure 3a are "bridged," acting as passivation centers, while the control NCs tend to fuse and aggregate, thereby introducing traps. This conclusion seems to lack experimental evidence. The large NCs shown in Figure 3a could very well result from the fusion of multiple NCs. In fact, the images in Figure 3c clearly indicate fused NCs. If the proposed bridging truly exists, one would expect to observe connections between multiple spherical NCs as schematically illustrated in Figure 1d and 1f, rather than a large crystal.

2. Both XPS and SIMS results show that the Ag content in the "Bridged NC" sample is hundreds of times higher than that of Pb; however, the TEM-EDX mapping in Figure 3c still exhibits a pronounced Pb signal, which is questionable. Please provide the corresponding EDX spectra and quantitative elemental ratios.

3. In the XPS spectra of the washed samples, the Pb signal is much weaker than those of Cs and I. How, then, could a perovskite structure form at the interface? This observation also contradicts the schematic illustration in Figure 1. The manuscript repeatedly emphasizes a Monolayer Perovskite Bridged configuration, yet no solid evidence is provided. The authors should not simply adopt the conclusions from the referenced PbS QD paper (doi.org/10.1016/j.joule.2020.05.011), since $AgBiS_2$ and PbS possess fundamentally different properties.

4. The authors state that GISAXS provides key information about the interparticle spacing under different conditions, from which they deduce the existence of a Monolayer Perovskite Bridged structure. However, the GISAXS data shown in Figures S9d–f do not reveal any clear differences. Please describe in detail how the results in Figures S9d–f were used to derive the conclusions presented in Figure 2d. Furthermore, the manuscript lacks specific experimental details for both GIWAXS and

GISAXS measurements.

5. The statement "The objective of this approach is to achieve efficient passivation on nonpolar (100) facets of $AgBiS_2$ NCs, while the (111) facets remain passivated by halides" is not sufficiently supported by evidence. Similarly, the claim that "prepared NC films can be annealed above 150 °C to achieve greater cation disorder, thus fully activating their light absorption capability while avoiding defect formation triggered by NC fusing" requires further justification. What is the basis for assuming that higher annealing temperature induces greater cation disorder, and how does this necessarily lead to enhanced light absorption capability?

6. In Figure S9a, the P-modified sample shows a noticeable shift of the (200) diffraction peak in GIWAXS compared to the control, whereas no obvious shift is observed in the XRD pattern (Figure S9b). Please explain this discrepancy.

Reviewer #3

(Remarks to the Author)
The authors describe a CsPbBr/I perovskite surface treatment for films of AgBiS2 nanocrystals for use as the active layer in a solar cell. They claim a peak certified efficiency of 11.22 % PCE (12.4 uncertified), which is higher than the previous highest claim for devices using AgBiS2 9.4 % certified (10.8 % uncertified). However, those other works didn't use lead perovskite, and the record for lead perovskite solar cells is >26%. Despite this issue in motivation, the work is interesting, appropriate to the readership of Nature Communications and will be of great interest to chemists and materials scientists working on AgBiS2 quantum dots. Additionally, there are several issues that must be addressed prior to publication, plus those issues raised by other reviewers.

1. The motivation for using AgBiS2 (environmentally friendly) is greatly weakened by adding lead. The authors need a different motivation to be more convincing. How is this 12% PCE solar cell better than other reported 12% solar cells (e.g. CZTS)? Why is this more interesting (to a general audience) than the far superior pure perovskite cells at more than 25%?

2. How much light is absorbed by the perovskite? The authors state this combination is "more than the sum of its parts", however it would be useful to see how much photocurrent is contributed given the relative amounts of each material. I think there is a risk that, even with the washing, the authors have produced two cells essentially in parallel, one made from perovskite, the other from AgBiS2. The perovskite one is much more efficient, but there is much less of it. The increase in PCE does not prove necessarily that the AgBiS2 is working better, just that they've added a material that makes good solar cells into the void space in the film. A stronger evaluation of the morphology of the thin film with perovskite, before and after washing (increasing void space?) is needed.

3. If the film is working as the authors suggest, with very little lead left over after washing, then it may be possible the CsX and PbX2 are not both necessary. Sargent et al, showed many years ago that simply adding halide salts to the surface of PbS QDs in solar cells would improve mobility and performance. I would suggest the authors try a few more controls, adding CsBr and CsI, and PbBr2 and PbI2, sequentially. Is it the Br or the I that is most effective? A range of mixtures should have been attempted. This is Nature, is it expected that you have explored the parameter space. It is entirely possible that you are building a halide layer between neighboring QDs and the perovskite in the film is incidental.

4. Some data on optimization of the transport layers is also expected in the SI.

5. The stability data is not very convincing. Solar cells in industry are tested according to very specific standards, like high temperature and humidity over many months. 25% humidity is fairly low. Given the propensity of perovskite to react with water and air, adding it to AgBiS2 should make the devices LESS stable not more, so this result is surprising, if indeed perovskite is forming. The argument that it is stabilized between QD surfaces is very vague, it's still a polar crystalline material and should still be soluble in polar solvents.

6. Why did the authors use ACN and not DMF or DMSO (or NMP) to wash away excess perovskite? Did they try other solvents?

7. If there is a crystalline structure being formed, then it should be visible in the XRD. If it is molecular than the corresponding shifts in the Pb and halide XPS should be visible. It is also possible to do Pb and Br NMR to prove the local environment (attached to the Ag/Bi rich surface) is what they say.

8. If the attachment interface is type I, why is charge separation improved. Shouldn't the exciton be trapped in the AgBiS2 core?

Version 1:

Reviewer comments:

Reviewer #2

(Remarks to the Author)
The authors have provided comprehensive responses to most of the reviewers' questions, and I generally agree with its

publication in Nature Communications. However, I still have several remaining concerns.

The authors have devoted significant effort to distinguishing between "Bridge" and "Fuse," yet the related explanations remain unconvincing. In fact, the TEM images in Figure 3a and 3b are typical of what is often observed in QDs even capped with long-chain ligands. Regarding Figure S13, why is the Pb signal undetectable at the fusion site? This seems unreasonable, as lead halide acts as a ligand and should theoretically be uniformly distributed across the entire NC.

Furthermore, why do the authors assume that NC fusion introduces traps (Figure 1f)? While this viewpoint is understandable for traditional PbS and PbSe QDs, where strong quantum confinement causes fusion to significantly alter the bandgap and potentially introduce mid-gap states. AgBiS2 exhibits much weaker quantum confinement, meaning fusion has a negligible effect on its bandgap. Therefore, I suggest the authors de-emphasize these descriptions. The record, certified PCE achieved in this work represents a significant advancement in the field, demonstrating the effectiveness of the proposed surface treatment, even if it is simply a matter of improved surface passivation.

Since the description has been revised to a "Molecular Perovskite layer" structure, is the lattice spacing matching still relevant? If the authors maintain that this is critical, please provide HRTEM images for other perovskite compositions after the exchange.

Additionally, I believe the authors should explicitly specify "lead halide perovskite" in both the title and the main text.

Reviewer #3

(Remarks to the Author)
This manuscript reports a method of improving the PCE of silver bismuth sulphide nanocrystal solar cells by adding lead perovskite to the active layer. Aside from the mildly questionable decision to add lead to a lead-free material, the film does appear to enable higher efficiency in the nanocrystalline solar cells, an improvement of between 1-2% depending on which previous/current measurements are to be believed. At this stage, the manuscript has been reviewed at least once and the authors have responded to all the questions, mostly successfully. Although a few minor questions remain, the major impediment to publication has been the use of perovskite as a additive transport material in the functional layer which has to some degree already been implemented previously with nanocrystal (lead sulphide) films. In my opinion, the small increase in efficiency, over previously reported lead-free cells, perhaps is not enough to warrant publication in this journal. It is clear the perovskite helps a little, but the mechanism does not appear very unusual or novel nor is it clear how this will lead to improvements beyond 12% PCE, which are critical to achieving the goal of supplanting other solar technologies (>20 % PCE). The following issues should also be addressed, prior to publication anywhere.

1) the authors suggest that the conduction is improved by adding the perovskite and bonding the NCs together, however the conduction mechanism is not strongly studied in this work or, to my knowledge, in previous works in adequate detail to discuss the charge transfer mechanism. Authors should cite previous work if possible, otherwise the mechanism (tunneling vs hopping) is vague as are the states involved in transport between nearest neighbors. What they suggest is reasonable, given what is known about perovskite, but this work is primarily an "additive leads to higher PCE" manuscript, not a transport study. The conduction mechanisms of silver bismuth sulphide nanocrystals, including low temperature and trap filling experiments, need to be better understood to make strong generalizations here.

2) Why lead perovskite? Why not Sn, since one imagines the improvement might be similar.

Version 2:

Reviewer comments:

Reviewer #2

(Remarks to the Author)
The authors have addressed my questions. I recommend publishing the manuscript in Nature Communications now.

Open Access This Peer Review File is licensed under a Creative Commons Attribution 4.0 International License, which permits use, sharing, adaptation, distribution and reproduction in any medium or format, as long as you give appropriate credit to the original author(s) and the source, provide a link to the Creative Commons license, and indicate if changes were made.
In cases where reviewers are anonymous, credit should be given to 'Anonymous Referee' and the source.
The images or other third party material in this Peer Review File are included in the article's Creative Commons license, unless indicated otherwise in a credit line to the material. If material is not included in the article's Creative Commons license and your intended use is not permitted by statutory regulation or exceeds the permitted use, you will need to obtain permission directly from the copyright holder.
To view a copy of this license, visit https://creativecommons.org/licenses/by/4.0/

Reviewer: 1

General comments: Most of the comments previously raised in the Nature Photonics review have been satisfactorily addressed, and I consider the current version suitable for publication in Nature Communications without further revision.

Author response: We would like to express our gratitude for the comment provided by this reviewer.

Reviewer: 2

General comments: The authors have thoroughly addressed the comments raised by the reviewers in the previous round, and the overall quality of the manuscript has been significantly improved. In particular, the authors have achieved the highest certified efficiency for $AgBiS_2$ nanocrystal solar cells. However, the so-called "Monolayer Perovskite Bridged" structure and its claimed remarkable effect remain unconvincing based on the current data and explanations provided in the manuscript. The authors introduced the heavy metal Pb into the environmentally friendly $AgBiS_2$ NC system. Although the authors claim that the amount is minimal, this still greatly undermines the environmental advantages of the $AgBiS_2$ NCs. Moreover, regarding novelty, the strategy and interpretations presented in this work closely follow those reported for PbS QD systems (doi.org/10.1016/j.joule.2020.05.011), and therefore lack sufficient originality. In summary, although this work achieves high device performance, substantial revisions are still required before it can be considered for publication in Nature Communications.

Author Response:

We are appreciative of the reviewer's contribution and suggestion.

In response to the reviewer's request, we have supplemented additional TEM characterization in the revised manuscript, and refined the corresponding descriptions to verify the existence of the perovskite bridge structure.

Also, taking in account the comments of the third reviewer, we have changed the description from <u>monolayer perovskite</u> to <u>molecular perovskite layer</u> throughout the text, and the dimensionality of the latter has been further confirmed by experimental evidence. Furthermore, we have revised the motivation from environmental considerations to emphasize its exceptional stability and the advantages of cost-effectiveness as suggested (doi.org/10.1016/j.scib.2023.01.031).

It should be noted that although the concept of perovskite bridging in this work is similar to that reported for PbS systems (doi.org/10.1016/j.joule.2020.05.011), there are several distinct differences. Based on the properties of $AgBiS_2$, we selected the $CsPbBr_3$ perovskite composition for its more appropriate lattice constant. The introduction of $CsPbBr_3$ was conducted by ligands, which is different from other methods.

With regard to the modulation of material properties, the introduction of the molecular perovskite layer between $AgBiS_2$ NCs primarily serves two key functions. Firstly, it enhances the carrier mobility of NCs film, thereby increasing the carrier diffusion length. This is also targeted in that PbS work (doi.org/10.1016/j.joule.2020.05.011). However, due to the long intrinsic carrier diffusion length of PbS and its propensity to form thick films, further thickness optimization (from 350 nm to 450 nm) via perovskite bridging results in only a marginal photocurrent enhancement (~2 mA cm^{-2}), indicating diminishing returns. In contrast, this approach leads to a much more significant enhancement for $AgBiS_2$, enabling the film thickness to be substantially increased from about 40 nm to 185 nm. This greatly improves its near-infrared EQE response, boosting the photocurrent by ~7 mA cm^{-2} (Figure 5g).

A more pronounced distinction lies in the second role of the molecular perovskite in the $AgBiS_2$ NCs film. The light absorption and carrier mobility of $AgBiS_2$ NCs show a positive correlation with the degree of cation disorder. While a higher annealing temperature increases cation disorder, excessively high temperatures can induce NC fusion and defect generation (doi.org/10.1002/adma.202305009; doi.org/10.1038/s41566-021-00950-4). We utilized the high thermal stability of the inorganic molecular perovskite layer between NCs to suppress their fusion along the (100) facets. This allows the annealing temperature to be increased to fully exploit the ultra-high light absorption potential of $AgBiS_2$ NCs without introducing defects caused by NC fusion. As a result, a further photocurrent increases of ~3 mA cm^{-2} was achieved by optimizing the annealing temperature from 115°C to 150°C (Figure 5d). This increase is attributed to a doubling of the carrier mobility and a substantial boost in light absorption (Figures 1e and 18a). This represents a feature unique to our $AgBiS_2$ system,

not observed in that PbS work involving $FAPbX_3$ incorporation.

The subsequent section details our meticulous response, addressing the concerns raised by the reviewers.

C2.1 The authors claim that the NCs in Figure 3a are "bridged" acting as passivation centers, while the control NCs tend to fuse and aggregate, thereby introducing traps. This conclusion seems to lack experimental evidence. The large NCs shown in Figure 3a could very well result from the fusion of multiple NCs. In fact, the images in Figure 3c clearly indicate fused NCs. If the proposed bridging truly exists, one would expect to observe connections between multiple spherical NCs as schematically illustrated in Figure 1d and 1f, rather than a large crystal.

Author response:

We thank the reviewer for the comments.

As suggested, we supplemented TEM images of spherical NCs with localized directional bridging (Figure 3a), whereas the control sample without molecular perovskite layer displays a disordered and fused morphology (Figure S13a). It is acknowledged that the schematic in Figure 1d represents an idealized bridged structure. In practice, during the process of formation, NCs not only bridge directionally but also occasionally along other directions of the (100) crystal plane family. This phenomenon may result in the manifestation of certain "large NCs" within the TEM images, as illustrated in the figures contained within the previous version of the submission. In the revised manuscript, we have supplemented the additional data and appropriately modified the relevant descriptions.

Furthermore, we suggest that the EDS of the Pb signal in Figure 3c serves as key evidence to confirm the bridged structure rather than a fused configuration. The lack of coverage with the ligand layer is a pivotal factor in the fusion of the nanocrystal interface, as illustrated in Figure 1f. It is well established that the Pb signal at the fused

interface would be significantly weaker than other regions with lead halide ligand adsorption, if the elongated NCs were formed by fusion. Our experimental observations revealed the aggregation of the Pb element precisely at the interfaces between nanocrystals (Figures 3b and 3c). This finding indicates the presence of higher concentrations of Pb-containing ligands at these interfaces. Consequently, the "molecular perovskite layer bridged $AgBiS_2$ nanocrystals" was proposed to describe this observation.

In order to further validate this effect, a control group of $AgBiS_2$ NCs was prepared and subjected to ligand exchange with a mixture of AgI, NaI and PbI_2. Rather than forming a perovskite phase, this specific mixture facilitates the adsorption of lead halide ligands onto the NC surfaces. As shown in the Figures S13d-e, the EDS analysis of the dimers formed with this ligand combination confirms the uniformly distributed Pb signals without specific aggregation. In addition, the Pb signal at the interfaces is significantly weaker than in the surrounding areas. The present findings provide further evidence in support of the hypothesis that the interfacial Pb accumulation in the bridged structure is unique.

The following sentences have been revised in the main text on pages 8-9: "The high-angle annular dark-field scanning TEM image and energy-dispersive X-ray spectroscopy (EDS) elemental mapping in Figure 3c reveal the uniform distributions of Ag and Bi throughout the bridged NCs. The Ag signal detected surrounding the bridged NCs (outside the yellow-framed area) originates from the adsorption of AgX_2^- ligands on the NC surface. Concurrently, the trace Pb signal exhibited a pronounced concentration, manifesting specifically at the bridging regions between individual NCs (Figures 3c and S13c). This interfacial Pb signal accumulation is indicative of a configuration formed *via* NC bridging rather than NC fusion, as interfacial ligand depletion in fused structures would diminish Pb signal (Figures S13d and S13e)."

Figures 3a and S13 were revised in the revised manuscript accordingly.

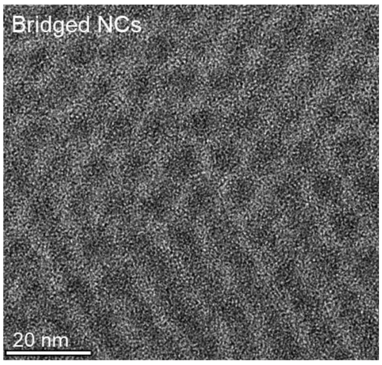

Figure 3. (a) High-resolution transmission electron microscopy (HR-TEM) image of the P-modified NCs after washing with ACN. Scale bar: 20 nm.

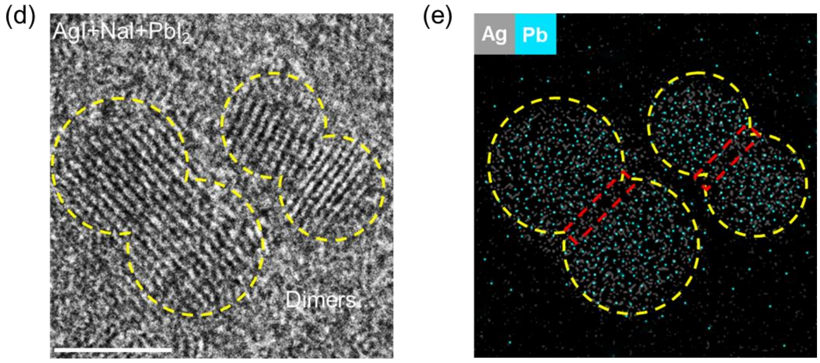

Figure S13. (d) HRTEM image of the fusing NCs dimer subjected to ligand exchange with the mixture of AgI, NaI, and PbI_2. This particular ligand combination does not permit the formation of a perovskite phase, but does allow for the adsorption of lead halide ligands onto the NC surfaces. Scale bars: 5 nm. (e) The corresponding EDS mapping for Pb and Ag elements. The images reveal a scarcity of Pb element at the fusion site, as highlighted within the red rectangular area.

C2.2 Both XPS and SIMS results show that the Ag content in the "Bridged NC" sample is hundreds of times higher than that of Pb; however, the TEM-EDX mapping in Figure 3c still exhibits a pronounced Pb signal, which is questionable. Please provide the corresponding EDX spectra and quantitative elemental ratios.

Author response:

We thank the reviewer for the comments.

In view of the markedly low Pb content, the dwell time for Pb was intentionally extended during EDS mapping in order to clearly resolve its accumulation at the interfaces between NCs, as noted in the corresponding figure caption.

As suggested, we have supplemented the EDS mapping and elemental quantification data of the Pb signal scanned concurrently with Ag and Bi in Figure S13c. However, due to the inherent limitations of this method for accurate quantification of trace elements, and the minimal amount of Pb present, the quantification results are subject to significant error and are provided for reference purposes only. A more precise quantitative dataset can be found in Table S3 of the ICP-OES results.

The following information have been added to the figure caption in Figure 3c: "The dwell time for the trace Pb signal was increased with the aim of improving the characterization of its spatial distribution at the interfaces."

Figures S13c were added in the revised manuscript accordingly.

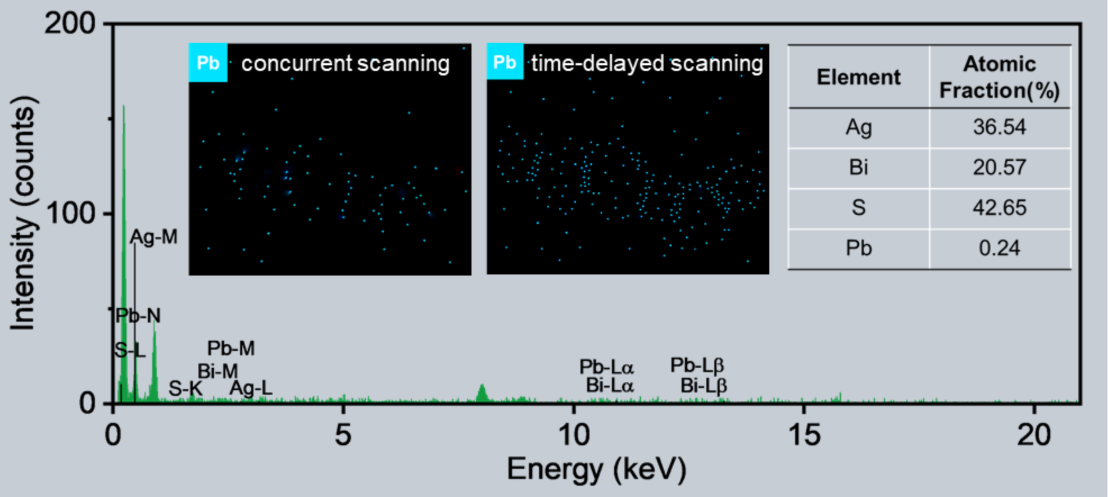

Figures S13. (c) EDS analysis of P-modified NCs after washing with ACN. The inset presents EDS mapping of Pb scanned simultaneously with Ag and Bi (Figure 3c), along with time-delayed EDS mapping of Pb (adapted from Figure 3c). The table shows the atomic percentages of each element during simultaneous scanning. Compared to the Ag content in the $AgBiS_2$-OA/OLA sample before ligand exchange (Table S2), the significant excess of Ag is attributable to the presence of silver halide ligands adsorbed on the NC surface.

C2.3 In the XPS spectra of the washed samples, the Pb signal is much weaker than those of Cs and I. How, then, could a perovskite structure form at the interface? This observation also contradicts the schematic illustration in Figure 1. The manuscript repeatedly emphasizes a Monolayer Perovskite Bridged configuration, yet no solid evidence is provided. The authors should not simply adopt the conclusions from the referenced PbS QD paper (doi.org/10.1016/j.joule.2020.05.011), since $AgBiS_2$ and PbS possess fundamentally different properties.

Author response:

We thank the reviewer for the comments.

We agree with the reviewer that the ligand layer on the NC surface is complex and diverse. This phenomenon is evidenced by the presence of the epitaxially grown perovskite monolayer ($CsPbX_3$) on the (100) facets, as well as other ligands on other surfaces such as adsorbed silver halides, lead halides, and Cs cations. These ligands provide elemental signals related to perovskite, which complicates the accurate distinction of signals originating from the perovskite monolayer from those stemming from other ligands. Therefore, direct and precise elemental analysis of the ultrathin molecular layer at the bridging interface is challenging. Following the suggestions from other reviewers, we have revised the description of the "monolayer perovskite" in the text to "molecular perovskite layer" for more precise characterization. We have a series of complementary experimental evidence that collectively supports the conclusion of the "molecular perovskite layer" structure.

The results of GISAXS measurements indicate that the inter-NC spacing after the washing process is reduced to 5.04 nm, and the calculated thickness of the bridging matrix is about 0.33 nm (Figure 2d). This is in close agreement with the dimensions of a monolayer perovskite (approximately half the lattice constant). This provides physical size evidence for the "molecular layer" structure.

In the results from the TAS testing on the samples before the washing process, the GSB

signal exhibited a peak near 560 nm. This is a typical characteristic peak of $CsPbBr_3$ perovskite. After the washing process, a weak GSB signal blue-shifted to 495-525 nm was observed (Figure 2f). This phenomenon can be attributed to the quantum confinement effect within the molecular layer. This result indicates that the perovskite material exists in a highly confined state rather than a bulk phase. Furthermore, the absence of perovskite crystallization peaks in the XRD pattern (Figure S10), combined with the shifted signals of Pb and Cs elements in the XPS analysis (Figure S9), collectively confirm the existence of the perovskite in the form of a molecular layer.

The HR-TEM image characterization on the samples clearly shows that the NCs are connected along the (100) facets through epitaxial growth, featuring with coherent and defect-free lattices at the interface (Figures 3b and 3c). This indicates that the lattice spacing of the molecular layer at the bridging interface exhibits a high degree of consistency with that of $AgBiS_2$. The lattice spacing for both of them is about 0.29 nm, a value that is almost identical to the (100) interplanar spacing of $CsPbX_3$ perovskite.

The aforementioned results suggest the existence of a molecular perovskite layer at the bridging interface. Furthermore, we have supplemented the explanation in the relevant part of the manuscript, emphasizing that this conclusion is based on the mutual confirmation of multiple characterization techniques, rather than a single piece of evidence or a simple inference based on the literature. As addressed in our response to the general comments, it is precisely this molecular-scale perovskite bridging that enables our $AgBiS_2$ NCs film to achieve not only a significant enhancement in carrier mobility, allowing the fabrication of thick films, but also a remarkable improvement in thermal stability due to the robust nature of the inorganic perovskite bridge matrix. This, in turn, unlocks the full potential of the $AgBiS_2$ NCs film in terms of both light absorption and charge transport properties.

The following changes have been made in the main text on pages 7: "The thickness of the perovskite matrix between the NCs (after washing) was estimated to be about 0.33 nm. This value was obtained by subtracting the diameter of the NC from the measured

inter-dot distance of near-monodisperse spherical NCs. This value closely matches the dimensions of the perovskite monolayer, i.e. about half of its lattice constant."

The following changes have been made to the main text on pages 8: "This phenomenon is attributed to the exciton confinement effect, indicating that the perovskite exists in a highly confined state rather than a bulk phase."

"In summary, the measured thickness of the perovskite matrix between the NCs (after a thorough washing process), the blue shift of the GSB signal, and the TEM images confirm that a molecular perovskite layer bridges the NCs *via* epitaxial growth along the (100) facet."

C2.4 The authors state that GISAXS provides key information about the interparticle spacing under different conditions, from which they deduce the existence of a Monolayer Perovskite Bridged structure. However, the GISAXS data shown in Figures S9d-f do not reveal any clear differences. Please describe in detail how the results in Figures S9d-f were used to derive the conclusions presented in Figure 2d. Furthermore, the manuscript lacks specific experimental details for both GIWAXS and GISAXS measurements.

Author response:

We thank the reviewers for their valuable comments.

By performing radial averaging on the two-dimensional scattering patterns (Figure S10d–f), the annular features were converted into a one-dimensional curve of intensity I(q) as a function of the scattering vector q, as shown in Figure 2d. The peak positions in the I(q) curve correspond to the inter-NC spacing. The relationship between the peak position q_{peak} and the inter-NC distance d is given by the modified Bragg's law:

$$d = \frac{2\pi}{q_{peak}}$$

By determining q_{peak}, the inter-NC distance between individual NCs can be obtained. Since the as-synthesized $AgBiS_2$ NCs are approximately spherical, the thickness of the

intermediate perovskite layer can be estimated by subtracting the NCs diameter (approximately 4.71 nm, obtained from Figure S5b) from this inter-NC distance.

As suggested, we have revised the GISAXS figures accordingly, with further elaboration provided on the procedure for calculating the interparticle distances in the corresponding figure captions.

Furthermore, comprehensive experimental parameters for both the GIWAXS and GISAXS measurements have been delineated in the Characterization section of the Supporting Information.

The following information have been added to the Characterization section of the Supporting Information: "The GIWAXS and GISAXS measurements were carried out with the Xeuss 3.0. The incident angle was set at 0.2°."

Figures S10 was revised in the revised manuscript accordingly.

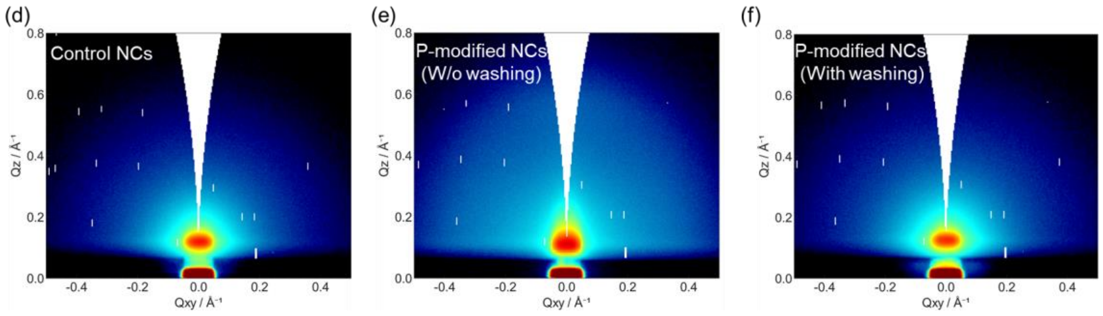

Figure S10. GISAXS 2D pattern of the control NC films (d), P-modified NC films without washing (e) and with washing (f). The horizontal linecut GISAXS profiles of the corresponding NC films are presented in Figure 2d. The inter-NC distance (d) was calculated using the formula $d = 2\pi/q$, q is the peak positions in the I(q) curve exhibited in Figure 2d.

C2.5 The statement "The objective of this approach is to achieve efficient passivation on nonpolar (100) facets of $AgBiS_2$ NCs, while the (111) facets remain passivated by halides" is not sufficiently supported by evidence. Similarly, the claim that "prepared NC films can be annealed above 150 °C to achieve greater cation disorder, thus fully

activating their light absorption capability while avoiding defect formation triggered by NC fusing" requires further justification. What is the basis for assuming that higher annealing temperature induces greater cation disorder, and how does this necessarily lead to enhanced light absorption capability?

Authors response:

We thank the reviewer for their comment.

As indicated by DFT calculations (Figure S7), the adsorption energy of halide salts on the (111) facets is significantly higher than that on the (100) facets. We suggested that, the washing process with ACN would result in the removal of perovskite molecules that are not chelated to the (100) facets, while the (111) facets would remain passivated by halide salts.

This finding is further substantiated by EDS mapping characterization (Figure 3c), which reveals that the overall distribution of the Ag signal appears slightly larger in the outer region compared to that of Bi. This observation confirms the adsorption of AgX_2^- ligands on the (111) facets, a finding consistent with the EDS quantitative analysis (Figure S13c), which indicates a significantly higher Ag content relative to Bi (Ag/Bi ≈ 1.8). In contrast, the Ag/Bi ratio in the pristine $AgBiS_2$-OA/OLA sample without ligand exchange is approximately 1.46 (Table S2).

As suggested, we have added corresponding boxes (yellow) in the Figure 3c in order to clearly indicate the adsorption and passivation by the outer AgX_2^- ligand layer on the surface. This discussion was moved to the Figure S13c caption in order to provide support for the argument being presented.

The following changes have been made in the main text on pages 8: "<u>The Ag signal detected surrounding the bridged NCs (outside the yellow-framed area) originates from the adsorption of AgX_2^- ligands on the NC surface."</u>

Figures 3c was revised in the revised manuscript accordingly.

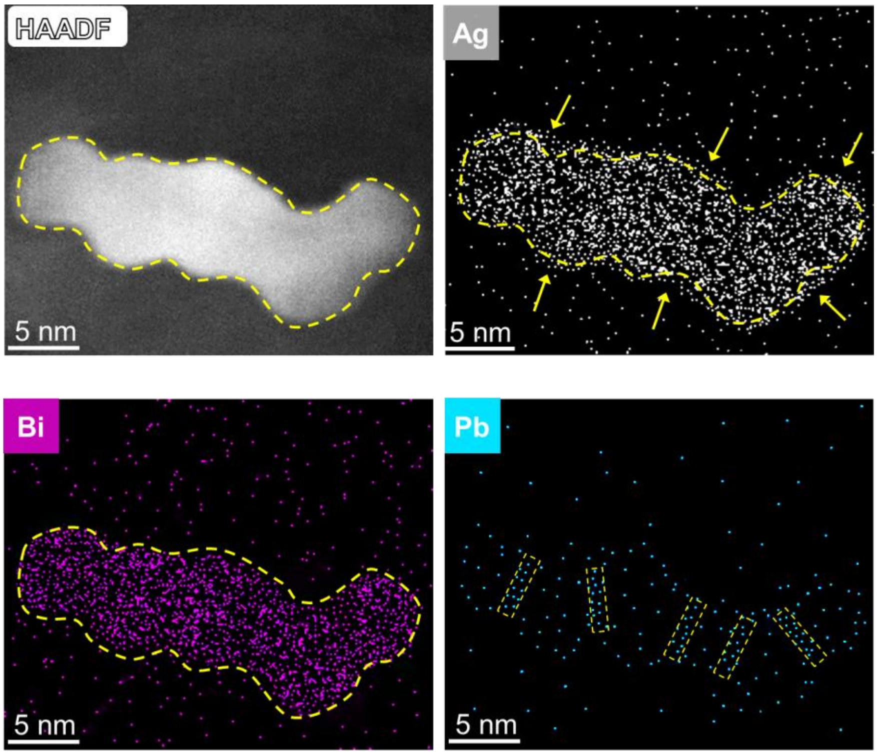

Figures 3. (c) High-resolution high-angle annular dark-field scanning TEM (HAADF-STEM) image and energy-dispersive X-ray spectroscopy (EDS) mapping of P-modified NCs after ACN washing. The dwell time for the trace Pb signal was increased with the aim of improving the characterization of its spatial distribution at the interfaces.

The following changes have been made to the Figure S13c caption: "Compared to the Ag content in the $AgBiS_2$-OA/OLA sample before ligand exchange (Table S2), the significant excess of Ag is attributable to the presence of silver halide ligands adsorbed on the NC surface."

Regarding the cation disorder induced enhancement of light absorption, this phenomenon has been extensively demonstrated in previous reports (doi.org/10.1038/s41566-021-00950-4). Increasing the annealing temperature can effectively promote cation disorder in $AgBiS_2$ NCs, which in turn modifies the band structure, increases the inter-band transition dipole moment, and ultimately results in enhanced light absorption. This cation disordering process exerts an influence on the

bond length distribution. Specifically, the cation disorder has been observed to shorten the average bond length, manifesting as a slight shift of XRD peaks toward higher angles.

As suggested, the supplementary XRD patterns of films annealed at varying temperatures provide further evidence to support the theory that temperature-induced cation disordering is the primary cause of the observed phenomenon. Figure S4 was added in the revised manuscript accordingly.

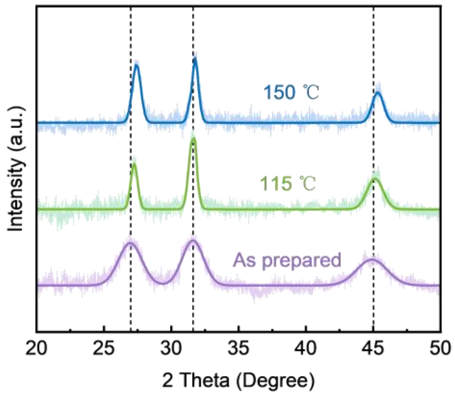

Figure S4. XRD patterns of the $AgBiS_2$ NCs films at various annealing temperatures. The occurrence of a cation disorder-induced contraction of the average bond length is evidenced by a consistent shift of the diffraction peaks to higher angles.

The following changes have been made in the main text on pages 5: "Supplementary XRD patterns of films annealed at varying temperatures provide further evidence for the temperature-induced cation disordering process (Figure S4)."

C2.6 In Figure S9a, the P-modified sample shows a noticeable shift of the (200) diffraction peak in GIWAXS compared to the control, whereas no obvious shift is observed in the XRD pattern (Figure S9b). Please explain this discrepancy.

Authors response:

We thank the reviewer for the comments.

In the 2D GIWAXS pattern, the (100) peak of the control sample is located at

approximately $q = 22.15$ nm^{-1}, corresponding to a lattice spacing of 2.84 nm ($d = 2\pi/q$). For the P-modified (W/o washing) sample, the value of q shifts to about 22.65 nm^{-1} (with $\Delta q \approx 0.50$ nm^{-1}), corresponding to a lattice spacing of 2.90 nm. In the XRD pattern, the (100) peak of the control sample appears at about $2\theta = 31.68°$, corresponding to a lattice spacing d of 2.82 nm ($2d$◊$\sin(\theta)$= 1.54 Å). For the P-modified (W/o washing) sample, the (100) peak shifts to $2\theta = 31.25°$ (with $\Delta 2\theta \approx 0.43°$), correspond to a lattice spacing d of 2.86 nm. Therefore, the XRD result is consistent with the GIWAXS data within a reasonable error margin. In the GIWAXS pattern, the q-axis spans in the range of 5–25 nm^{-1}. The percentage of change of q can thus be evaluated to be 2.5% (= Δq/q = 0.50/20) in the whole measurement range. In the XRD pattern, the 2θ-axis spans in the range of 10–60°. The percentage of change of 2θ can thus be evaluated to be 0.83% (=$\Delta(2\theta)/(2\theta)$ = 0.43/50)) in the whole measurement range. This indicates that the visual difference in the peak shift is more pronounced in the GIWAXS plot.

As suggested, we have marked the positions for the corresponding peaks in Figure S10 for easier comparison. Figures S10a-b was revised in the revised manuscript accordingly.

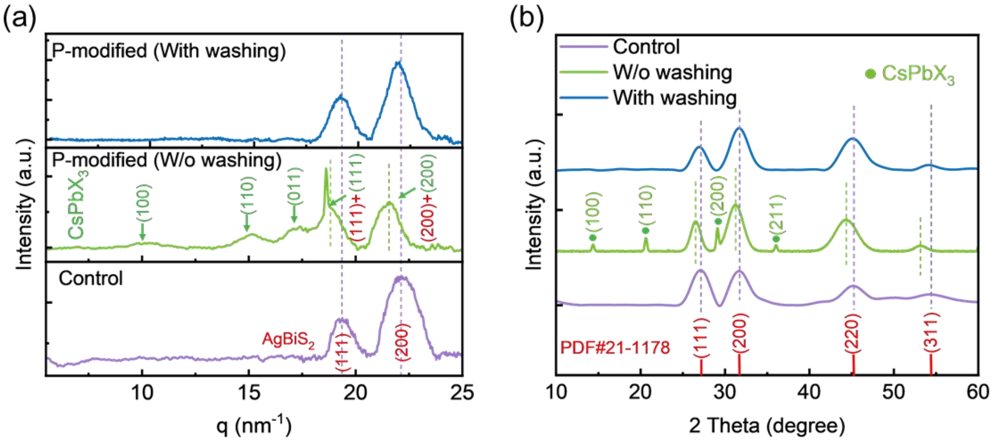

Figures S10. (a) GIWAXS line profiles of the control NCs film, P-modified NC films with/without ACN washing. The shift of the patterns (dash line marked, corresponding to $AgBiS_2$ diffraction) to lower q values in P-modified NC films with /without ACN washing suggests the $CsPbX_3$ perovskite layer growth on NC surfaces, forming the

strain due to the lattice mismatch between perovskite and $AgBiS_2$, where the lattice constant of $CsPbBr_3$ (5.85 Å) and $CsPbI_3$ (6.21 Å) have larger value than $AgBiS_2$ (5.69 Å). (b) XRD results of control NC films and P-modified NC with/without ACN washing films.

Reviewer: 3

General comments: The authors describe a CsPbBr/I perovskite surface treatment for films of AgBiS2 nanocrystals for use as the active layer in a solar cell. They claim a peak certified efficiency of 11.22 % PCE (12.13% uncertified), which is higher than the previous highest claim for devices using AgBiS2 9.4 % certified (10.8 % uncertified). However, those other works didn't use lead perovskite, and the record for lead perovskite solar cells is >26%. Despite this issue in motivation, the work is interesting, appropriate to the readership of Nature Communications and will be of great interest to chemists and materials scientists working on AgBiS2 quantum dots. Additionally, there are several issues that must be addressed prior to publication, plus those issues raised by other reviewers.

Response:

We appreciate the reviewer's suggestions.

In the revised manuscript, we have shifted the focus of the motivation from environmental considerations to emphasize its cost-effective, excellent stability and the advantages of low-temperature solution processability. Below are our detailed responses to each comment.

C3.1 The motivation for using AgBiS2 (environmentally friendly) is greatly weakened by adding lead. The authors need a different motivation to be more convincing. How is this 12% PCE solar cell better than other reported 12% solar cells (e.g. CZTS)? Why is this more interesting (to a general audience) than the far superior pure perovskite cells at more than 25%?

Author response:

We thank the reviewer for their comment.

$AgBiS_2$ exhibits an ultrahigh absorption coefficient, a property that facilitates the

reduction of the thickness of active layers to a substantially less than that achievable with conventional photovoltaic materials (doi.org/10.1016/j.scib.2023.01.031). Furthermore, the fabrication process under consideration employs comparatively low temperatures (i.e. < 150 °C). The material's high tolerance to compositional and structural disorder makes the synthesis of $AgBiS_2$ NCs and the preparation of high-quality thin films, rendering the process simpler and more reproducible. It is noteworthy that the $AgBiS_2$ material exhibits excellent ambient stability. CZTS typically necessitates micron-thick active layers. In the fabrication of highly efficient solar cell, the processes is highly sensitive to chemical composition and involve complex processing at elevated temperatures (> 400 °C).

We fully acknowledge that the incorporation of lead (Pb) into the film has the potential to compromise the assertion of environmental sustainability. However, the Pb content in NCs films (after the washing process) is extremely low and complies with the EU RoHS standard. From a lifecycle perspective, $AgBiS_2$ is a more environmentally sustainable alternative than CZTS (which contains toxic cadmium or selenium) or lead-based perovskites (where lead is a primary component).

As suggested, and combined with the established characteristics of $AgBiS_2$ summarized in the literature (doi.org/10.1039/D4EE03266G; doi.org/10.1016/j.scib.2023.01.031), we have revised the manuscript to highlight low-cost fabrication, low-temperature solution processability, and high stability as key motivations for this study.

The following changes have been made to the abstract on pages 1: "Ternary chalcogenide $AgBiS_2$ nanocrystals have emerged as an environmentally friendly and stable material for ultra-thin film lightweight low-cost solar cells."

The following changes have been made to the main text on pages 2: "$AgBiS_2$ nanocrystals (NCs) have attracted considerable interest for high efficiency and stable photovoltaics due to their cost-effective, high absorption (>10^5 cm^{-1}) and low-temperature solution processability.[1-3]"

C3.2 How much light is absorbed by the perovskite? The authors state this combination is "more than the sum of its parts", however it would be useful to see how much photocurrent is contributed given the relative amounts of each material. I think there is a risk that, even with the washing, the authors have produced two cells essentially in parallel, one made from perovskite, the other from AgBiS2. The perovskite one is much more efficient, but there is much less of it. The increase in PCE does not prove necessarily that the AgBiS2 is working better, just that they've added a material that makes good solar cells into the void space in the film. A stronger evaluation of the morphology of the thin film with perovskite, before and after washing (increasing void space?) is needed.

Authors response:

We are very grateful for the reviewer's suggestions.

The experimental data obtained in this study consistently demonstrate that the enhancement in performance is attributed to the enhanced intrinsic properties of the bridged $AgBiS_2$ NCs, rather than to a parallel perovskite cell mechanism.

The enhancement of photocurrent is primarily observed in the infrared region (>800 nm), as demonstrated by the EQE spectrum (Figure 5g). It should be noted that the $CsPbBr_3$ perovskite absorbs only below 550 nm (Figure S8f), precluding its contribution to infrared photocurrent generation. This spectral mismatch confirms that the observed current gain is attributed to enhanced infrared absorption within $AgBiS_2$ film itself.

Structurally, GISAXS measurements reveal a bridge thickness of ~0.33 nm (Figure 2d), which corresponds to the dimension of a molecular perovskite rather than that of a bulk-phase perovskite cell with independent functionality. It is of particular significance that the unwashed film containing a greater proportion of perovskite shows a lower efficiency (Figure S2), thereby directly contradicting the parallel cell hypothesis. Collectively, these results collectively verify that interface engineering, rather than

perovskite contribution, enables the performance enhancement. We have incorporated these clarifications into the revised manuscript to strengthen the discussion.

As suggested, we have supplemented the absorption spectrum of pure $CsPbBr_3$ perovskite films and a comparison of the light absorption between the perovskite-modified NC films before and after washing, along with control samples annealed at the same temperature (Figure S8f). The results show that the unwashed perovskite-modified NCs film exhibits only a marginal increase in absorption within the sub-550 nm region, likely originating from minimal absorption by the trace $CsPbX_3$ perovskite species. In contrast, no discernible difference in light absorption was observed between the washed perovskite-modified NCs film and the control sample, thus contributing negligibly to the overall photocurrent.

The following changes have been made to the main text on pages 5-6: "It is noteworthy that the freshly prepared inks of the control and P-modified NCs showed almost identical UV-Vis spectra, with no absorption features related to perovskite observed in the latter (Figure S8f).[34-36] Furthermore, the perovskite-modified NCs film before ACN washing exhibits a slight enhancement in light absorption at wavelengths below 550 nm, likely originating from minimal absorption by the trace $CsPbX_3$ perovskite. The absorption became virtually identical to the control sample after washing with ACN."

The following changes have been made to the main text on pages 14: "The enhancement of J_{SC} is primarily attributable to the substantial improvement in EQE within the near-infrared region (800–1200 nm), far outside the absorption range of $CsPbX_3$ perovskite."

Figures S8f was added in the revised manuscript accordingly.

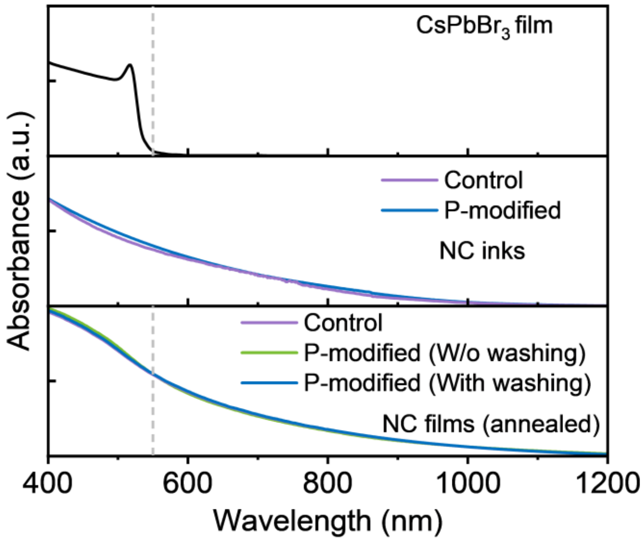

Figures S8. (f) (Top) Absorption spectra of $CsPbBr_3$ perovskite films. (Middle) Absorption spectra of the control and P-modified NC inks in the freshly prepared state. The almost identical UV-Vis spectra can be attributed to the fact that the perovskite precursors are unable to adsorb on the surface of NCs in the form of photoactive phase, and the amount of adsorber is too small to affect absorption. (Bottom) Comparison of the light absorption between the P-modified NC film before and after washing, and the control NCs film. All samples were annealed at 150°C.

C3.3 If the film is working as the authors suggest, with very little lead left over after washing, then it may be possible the CsX and PbX2 are not both necessary. Sargent et al, showed many years ago that simply adding halide salts to the surface of PbS QDs in solar cells would improve mobility and performance. I would suggest the authors try a few more controls, adding CsBr and CsI, and PbBr2 and PbI2, sequentially. Is it the Br or the I that is most effective? A range of mixtures should have been attempted. This is Nature, is it expected that you have explored the parameter space. It is entirely possible that you are building a halide layer between neighboring QDs and the perovskite in the film is incidental.

Authors response:

We are very grateful for the reviewer's suggestions.

The fundamental principle for selecting halide salt ligands in NCs involves matching the cation with the constituent elements. For example, lead halides are widely used in the preparation of PbS NCs, while silver halides are employed for the $AgBiS_2$ NCs. The utilization of a hetero-cation as the main ligand carries the potential risk of its diffusion in solutions and the adsorption onto the NC surface, which may result in lattice distortion or unintended ion exchange. This, in turn, could introduce new defects rather than merely passivation of existing ones. Therefore, it is highly desirable to employ a combination of suitable cation and halide anion, which can minimize this risk. In view of these observations, we propose the incorporation of additional halide salts, taking into consideration of the poor solubility of silver halides in DMF. These complexes can facilitate the formation of soluble AgX_2^- complexes, thereby enabling effective ligand exchange. The reported ligand systems for $AgBiS_2$ mainly comprise AgX + MAX or AgX + NaX. Therefore, the utilization of AgX + MAX as the control was deemed appropriate. In this study, we used CsX for two distinct purposes: provide halides for the dissolution of AgX and to supply Cs^+ for the formation of $CsPbX_3$ perovskite bridges.

The effectiveness of passivation varies with different halides, as shown in Figure S6f. Bromide salts exhibit stronger adsorption on the nanocrystal surface compared to iodide salts. This finding suggests the former may offer superior passivation properties. However, the extremely low solubility of bromide salts in solvents presents a practical limitation, necessitating the use of a predominantly iodide-based ligand solution.

As suggested, we have supplemented the efficiency distribution statistics for devices fabricated using both the control (AgX+MAX) and target (AgX+CsX+PbX_2) groups with varying halide ratios (Figure S25). The results indicate that both systems achieve optimal device performance at an I:Br ratio of ~5:1. Furthermore, an investigation was conducted into the effect of perovskite component (CsBr+$PbBr_2$) dosage in the target group, revealing an optimal addition amount of 0.02 mmol, corresponding to a $PbBr_2$/AgI molar ratio of 1/50.

The following changes have been made to the main text on pages 11-12: "An optimal halide ratio of I to Br at approximately 5:1 was used in the ligand solution for device fabrication, complemented by the addition of 0.02 mmol $CsPbBr_3$ (Figure S25)."

Figure S25 was added in the revised manuscript accordingly.

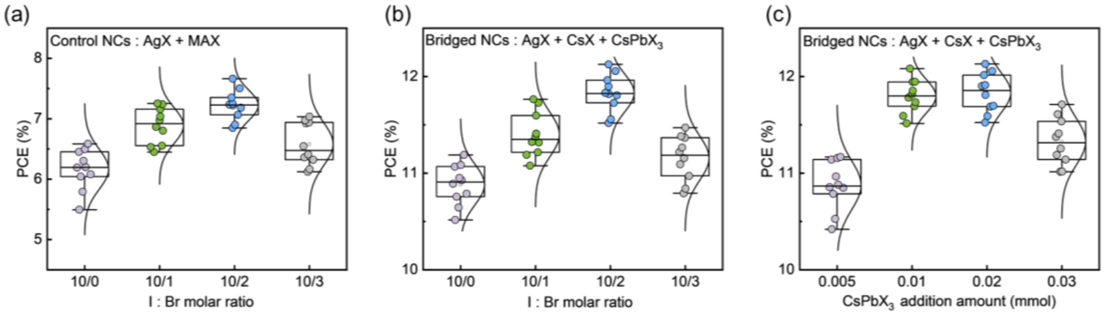

Figure S25. Optimization of halide composition in ligand systems for (a) control and (b) bridged NCs, with optimal AgI: AgBr ratio of 5:1 for both systems. The perovskite component in the bridged NCs ligand system was fixed at 0.02 mmol CsBr + 0.02 mmol $PbBr_2$. (c) Optimization of perovskite component dosage in bridged NCs, showing optimal performance at 0.02 mmol $CsPbBr_3$ equivalent.

As suggested, we prepared additional control ligand solutions: AgX + CsX and AgX + PbX_2. The former combination (AgX + CsX) has been verified to exhibit poor ligand exchange efficiency (Figure S6e). Devices fabricated using these two ligand combinations showed significantly reduced efficiency compared to the (AgX + MAX) control group. Relevant performance parameters are summarized in Table S10. These controlled experiments further confirm that the performance enhancement observed in this study is specifically attributed to the molecular perovskite bridge structure, rather than generic lead halide ligand passivation.

The following changes have been made to the main text on pages 12: "Control experiments with alternative ligand combinations demonstrate that the performance enhancement specifically stems from the designed molecular perovskite bridge structure, rather than from simple lead halide ligand passivation (Table S10)."

Table S10 was added in the revised manuscript accordingly.

Table S10. Photovoltaic performance of $AgBiS_2$ solar cells fabricated with different ligand combinations [a].

Ligands ensemble	AgX+MAX	AgX+CsX	AgX+PbX_2
J_{SC} (mA cm^{-2})	24.7<br>(24.2 ± 0.6)	23.2<br>(22.8 ± 0.5)	22.6<br>(21.7 ± 0.7)
V_{OC} (V)	0.482<br>(0.479 ± 0.004)	0.457<br>(0.448 ± 0.006)	0.445<br>(0.439 ± 0.005)
FF (%)	65.3<br>(64.1 ± 0.9)	61.2<br>(60.3 ± 0.8)	60.2<br>(59.6 ± 0.7)
PCE (%)	7.77<br>(7.43 ± 0.41)	6.49<br>(6.06 ± 0.47)	6.05<br>(5.58 ± 0.49)

[a] Values in parentheses indicate average and standard deviation of each parameter collected from 15 devices.

C3.4 Some data on optimization of the transport is also expected in the SI.

Authors response:

Based on established methodologies from high-performance $AgBiS_2$ NC photovoltaic devices, we employed SnO_2 as the electron transport layer and PTAA as the hole transport layer. A thin layer of $AgBiS_2$ NCs treated with 1,2-ethanedithiol (EDT) was used as a buffer interlayer. SnO_2 has been demonstrated to outperform alternative electron transport layers such as ZnO, while PTAA enables superior interfacial contact and charge transport compared to other polymers like PTB7. The selected transport layer provides optimal energy level alignment and enhanced interfacial contact, representing the currently preferred configuration for $AgBiS_2$ solar cells. (doi.org/10.1039/D4EE03266G; doi.org/10.1038/s41566-021-00950-4; doi.org/10.1021/acs.chemmater.0c03126)

Corresponding experimental details have been added to the Supporting Information.

The ETL process parameters have been slightly optimized.

As suggested, we supplemented the influence of SnO_2 thickness (controlled by spin speed) on device efficiency. The optimized SnO_2 layer thickness is approximately 26 nm (Figure S34).

The following changes have been made to the "Device Fabrication" part in Supporting Information: "After assembling $AgBiS_2$ NC films, a buffer layer of 1,2-ethanedithiol (EDT)-capped $AgBiS_2$ NCs, prepared via the SSLE method, was subsequently deposited on top."

Figure S34 was added in the revised manuscript accordingly.

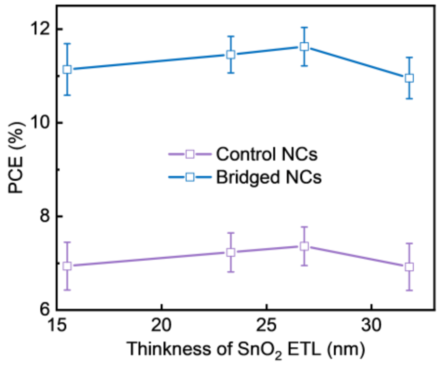

Figure S34. The performance of devices fabricated with different SnO_2 thicknesses was compared, with the results showing optimal performance at approximately 26 nm. The thickness was measured by atomic force microscopy.

C3.5 The stability data is not very convincing. Solar cells in industry are tested according to very specific standards, like high temperature and humidity over many months. 25% humidity is fairly low. Given the propensity of perovskite to react with water and air, adding it to AgBiS2 should make the devices LESS stable not more, so this result is surprising, if indeed perovskite is forming. The argument that it is stabilized between QD surfaces is very vague, it's still a polar crystalline material and should still be soluble in polar solvents.

Authors response:

We thank the reviewer for their valuable comments.

Whilst the conventional perovskite bulk films encounter stability issue, the present study exhibits a distinctly different behavior due to its unique structural configuration. The $CsPbBr_3$ molecular employed in this study is recognized as one of the most stable halide perovskite. The fabrication of $CsPbBr_3$ under ambient atmospheric conditions has demonstrated considerable resistance to moisture and oxygen degradation.

The perovskite in this study exists as an atomically coherent molecular bridge epitaxially grown between $AgBiS_2$ NCs, rather than as a conventional bulk film. This architectural design under discussion here enables enhanced stability through two key mechanisms. The epitaxial lattice anchoring to the (100) facets of $AgBiS_2$ NCs, requires substantial energy to disrupt the interface. Furthermore, the spatial confinement within the ~0.33 nm gap between NCs minimizes environmental exposure.

The washing process with ACN plays a critical role in this stability. This process is effective in the removal of excess, bulk-like perovskite matrix, resulting in the retention of a coverage of molecular perovskite layer. The resulting structure is characterized by the absence of extensive surface area, which are typically presented in polycrystalline films and serve as infiltration pathways for moisture and oxygen. Furthermore, the observation that the perovskite bridges withstand the polar ACN washing process itself provides direct evidence of their anchoring and dissolution resistance, thus demonstrating their stability.

As suggested, we have added corresponding discussions and stability tests, showing excellent device performance retention after one month at 80±5% RH, thus further validating the stability claims. The following changes have been made to the main text on pages 14: "Furthermore, the bridged NC devices also exhibited excellent stability under high humidity conditions (75–85%) over a one-month period (Figure S32)."

Figure S32b was added in the revised manuscript accordingly.

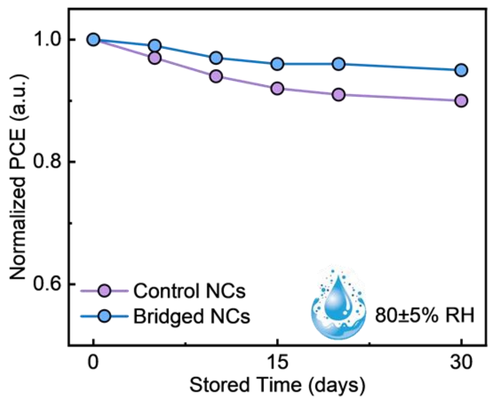

Figure S32. (b) Excellent retention of bridged NCs device performance after one-month storage at high humidity (80 ± 5% RH), attributable to the stable, epitaxially anchored molecular perovskite bridge that effectively impedes moisture ingress.

C3.6 Why did the authors use ACN and not DMF or DMSO (or NMP) to wash away excess perovskite? Did they try other solvents?

Authors response:

We thank the reviewer for their comment regarding solvent selection.

The selection of a suitable washing solvent was a meticulous process, undertaken with the utmost care and consideration. The surface of the bridged NCs contains not only the molecular perovskite layer but also a substantial amount of halide salt ligands (such as AgX and PbX) adsorbed on other surfaces for passivation. The utilization of a highly polar protic solvent during the washing process causes significant ligand desorption and compromises the integrity of the NCs film. The key advantage of the relatively weakly polar aprotic solvent ACN is its capacity to effectively dissolve excess free perovskite matrix without disrupting the molecular perovskite layer bridges epitaxially anchored to the $AgBiS_2$ NC surfaces or causing damage to the $AgBiS_2$ NC film itself. Furthermore, the relatively low boiling point of ACN (82°C) is significantly lower than

those of DMF (153°C), DMSO (189°C), and NMP (202°C). After the washing process, ACN rapidly evaporates from the film, thereby minimizing its residual time within the film and avoiding potential adverse effects such as film swelling, structural reorganization, or secondary crystallization that could result from prolonged immersion.

As suggested, we have conducted the DMF and DMSO control experiment. The results obtained from this study unequivocally demonstrate that these highly polar solvent solvents have a complete dissolution effect on NCs. This finding indicates that utilizing these solvents during the washing process would not only result in the removal of the perovskite material but also lead to significant damage to the structural integrity of the NC film itself.

Figure S3a was added in the revised manuscript accordingly.

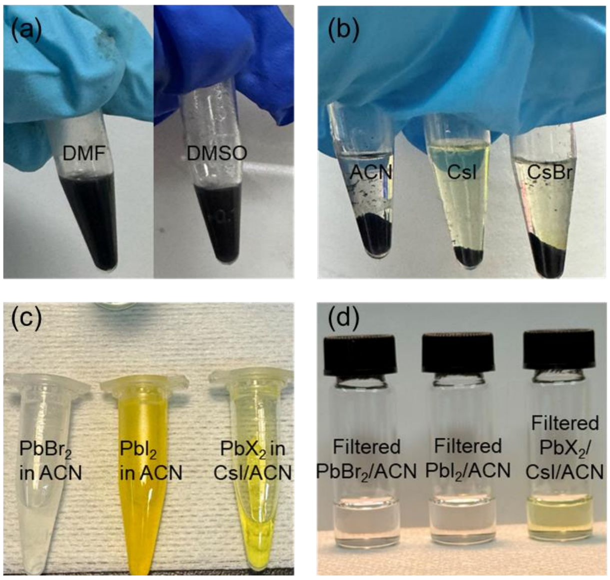

Figure S3. (a) (a) AgX+PbX capped $AgBiS_2$ NC solids merged in pure DMF and DMSO solvent, respectively. Both solvents completely dissolved the NC solids. (b) AgX+PbX capped $AgBiS_2$ NC solids merged in pure ACN solvent (left), CsI/ACN (middle, 5mg/mL), and CsBr/ACN (right, 5mg/mL); this indicates that ACN does not dissolve PbX ligand. To verify the insolubility of PbX ligands on the NC surface in ACN, we replaced CsX in the ligand solution with NaX to eliminate interference from color changes caused by perovskite dissolution. (c) 20mg $PbBr_2$ (left) pellets, 20mg

PbI_2 (middle) pellets in 1 mL of ACN solvent and CsI/ACN (5mg/mL) after 30 s vortex; this indicates that CsI /ACN dissolves PbX_2 and dynamically converts into perovskite. (d) Colorless solution of $PbBr_2$ (left) and PbI_2 (middle) in ACN after filter, transparent light-yellow solution of PbX_2 in CsI/ACN (right) after filter. (c) and (d) indicate that ACN can partially dissolve perovskite.

C3.7 If there is a crystalline structure being formed, then it should be visible in the XRD. If it is molecular than the corresponding shifts in the Pb and halide XPS should be visible. It is also possible to do Pb and Br NMR to prove the local environment (attached to the Ag/Bi rich surface) is what they say.

Authors response:

Thank you very much for your suggestion.

As suggested, we have revised the description of the "monolayer perovskite" in the text to "molecular perovskite layer" for more precise characterization. We prepared a pure $CsPbBr_3$ thin film and characterized its Cs 3d and Pb 4f signals using XPS. A comparative analysis was conducted with the Cs and Pb spectral peaks of perovskite molecules incorporated into the $AgBiS_2$ NC film, revealing distinct shifts in the binding energies of Cs and Pb signals towards higher energy levels in the latter case. This observation provides clear evidence of interfacial interaction between the molecule perovskite layer and $AgBiS_2$ NCs. Furthermore, the binding energies of Ag 3d and Bi 4f in the P-modified NC films shift significantly to lower values compared to those in the control NC films. This shift is attributed to the electron-rich state of Ag and Bi atoms, which is the result of the introduction of perovskite within the NC films. The results obtained demonstrate the modified electronic environment at the interface.

Figure S9 has been revised accordingly in the manuscript, and its caption has been updated to incorporate the corresponding descriptions.

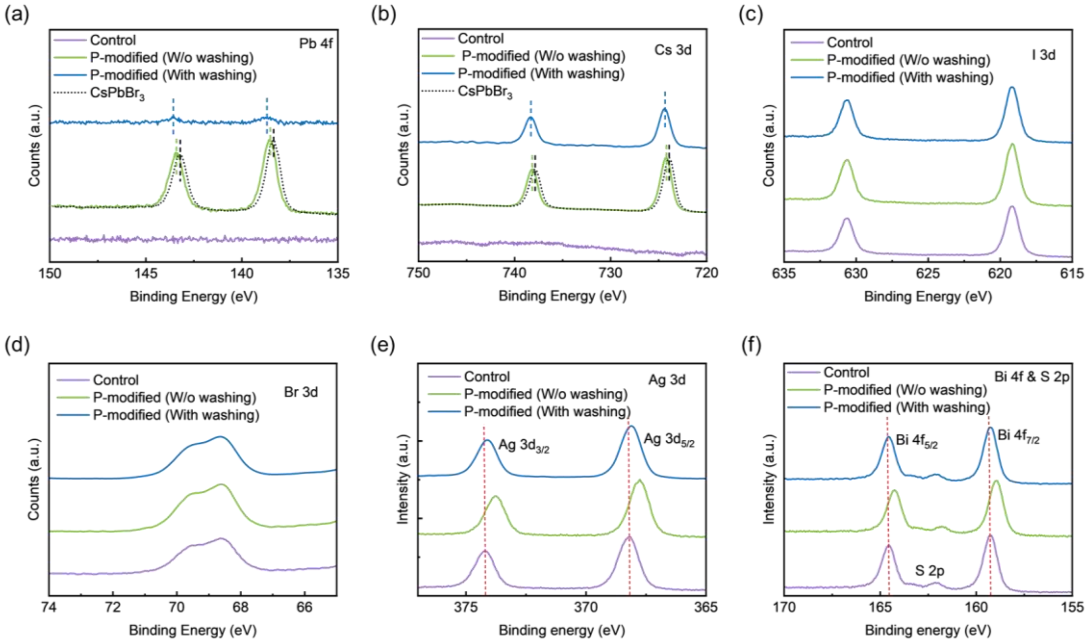

Figure S9. High-resolution XPS spectra of (a) Pb 4f, (b) Cs 3d, (c) I3d, (d) Br 3d, (e) Ag 3d, and (f) Bi 4f & S 2p for the control NCs film, P-modified NC films with/without ACN washing. Figures S9a and S9b additionally display the Cs 3d and Pb 4f XPS signals from a pure $CsPbBr_3$ thin film for reference. Comparison with the corresponding peaks in the P-modified NC films reveals that both the Cs and Pb signals in the latter shift toward higher binding energies. This observation confirms the interaction between the molecule perovskite layer and $AgBiS_2$ NCs. Furthermore, the binding energies of Ag 3d and Bi 4f in the P-modified NC films shift significantly to lower values compared to those in the control NC films. This shift is attributed to Ag and Bi atoms becoming electron-rich due to the introduction of perovskite within the NC films. These results collectively demonstrate the modified electronic environment at the interface.

C3.8 If the attachment interface is type I, why is charge separation improved. Shouldn't the exciton be trapped in the AgBiS2 core?

Authors response:

We fully agree that a Type-I band alignment is unfavorable for the process of charge separation in bulk heterojunctions, a finding that is in alignment with the observations made. For these NC film fabricated without the washing process (i.e. thick perovskite

coverage), charge carriers are confined within the $AgBiS_2$ cores (Type-I localization) and must also traverse a relatively thick inter-NC barrier. These factors lead to bad performance, as shown in Figure S3.

In these NC films fabricated with the washing process (i.e. the bridged NCs structure), the enhancement in performance is primarily attributable to a fundamental optimization of the charge-transport pathway. In conventional NC films, carriers travel via a hopping mechanism across the insulating barriers between NCs. The height of these barriers is exponentially dependent on the inter-NC spacing (Figure S18c). The molecular perovskite bridge formed after the washing process significantly reduces the inter-NC spacing from about 5.59 nm (unwashed) to about 5.04 nm (Figure 2d). This geometrical change greatly lowers the tunneling barrier that carriers must overcome, thereby markedly enhancing inter-dot charge transport. The data presented in Figure 4c, which illustrates the Arrhenius fit of the activation energy for carrier migration, provides direct evidence for this hypothesis. Therefore, within this bridged system, the pronounced enhancement in charge transport prevails over the deleterious effect of carrier localization imposed by the Type I band alignment.

As suggested, we have integrated the relevant descriptions into the manuscript to clarify and strengthen the chain of evidence.

The following information have been added to the figure caption in Figure S18c "The height of the potential barrier (activation energy) that needs to be overcome for hopping transport in the control NC films is significantly higher than that in the bridged NC films, making the transport process prone to being captured by defect states. Bridging reduces the spacing, which drastically lowers the tunneling barrier and activation energy. This, in turn, enhances transport efficiency despite the presence of Type-I band alignment."

(c)

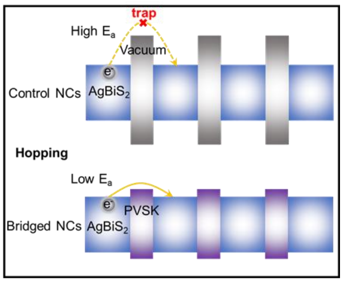

Figure S18. (c) Schematic diagrams of charge transport in the control NC and bridged NC films. The height of the potential barrier (activation energy) that needs to be overcome for hopping transport in the control NC films is significantly higher than that in the bridged NC films, making the transport process prone to being captured by defect states. Bridging reduces the spacing, which drastically lowers the tunneling barrier and activation energy. This, in turn, enhances transport efficiency despite the presence of Type-I band alignment.

Reviewer #2

General comments: The authors have provided comprehensive responses to most of the reviewers' questions, and I generally agree with its publication in Nature Communications. However, I still have several remaining concerns.

Author Response:

We would like to express our gratitude for the comment provided by this reviewer. The subsequent section details our meticulous response, addressing the concerns raised by the reviewers.

C2.1 The authors have devoted significant effort to distinguishing between "Bridge" and "Fuse," yet the related explanations remain unconvincing. In fact, the TEM images in Figure 3a and 3b are typical of what is often observed in QDs even capped with long-chain ligands. Regarding Figure S13, why is the Pb signal undetectable at the fusion site? This seems unreasonable, as lead halide acts as a ligand and should theoretically be uniformly distributed across the entire NC.

Author Response:

We would like to express our gratitude to the reviewer for the valuable suggestions.

Theoretically speaking, both the fusion along the ligand-deficient (100) crystal planes and the bridging along the (100) planes of $AgBiS_2$ NCs essentially involve the connection of multiple nanocrystals. Therefore, from a morphological perspective alone, it is indeed difficult to distinguish between these two connection mechanisms. However, the elemental mapping analysis of the elongated nanocrystals provides significant evidence that supports the bridging mechanism, specifically the aggregation of Pb elements between the nanocrystals (Figure 3c). To further highlight this characteristic of the bridged nanocrystals, we have supplemented additional Cs-corrected scanning dark-field TEM images and the corresponding EDS elemental line scans to confirm the aggregation of Pb elements at the bridging interface (Figure S13a).

Furthermore, as the reviewer noted, the spontaneous fusion of nanocrystal materials along ligand-deficient planes can result in the formation of similar morphologies. However, in experiments conducted with $AgBiS_2$ nanocrystals devoid of added perovskite, the well-defined oriented attachment morphology exhibited in Figures 3a and 3b was not observed. Conversely, the presence of such oriented attachment morphology was observed in $AgBiS_2$ nanocrystals with added perovskite.

With regard to the diminished Pb signal at the fusion interface (Figure S13f), it is hypothesized that this is attributable to two primary factors. Firstly, the fusion interface itself is ligand-deficient, a prerequisite for fusion to occur. Secondly, the fusion process has the potential to generate an unfavorable spatial environment, including grooves around the interface and atomic rearrangements. These phenomena may further inhibit ligand adsorption, consequently leading to a reduction in the Pb signal.

The following sentences have been modified in the main text on page 9 lines 5-9: "The HR-TEM images show that the NCs are effectively bridged to one another (Figures 3a and S13a), while the control NCs are significantly fused and aggregated (Figures S13b and S13c). The presence of such dimers has been shown to introduce numerous trap states at the interface, with the consequence that this affects the charge transport."

As suggested, the following sentence has been added to the caption of Figure S13f: "The images reveal a scarcity of Pb element at the fusion site, as highlighted within the red rectangular area. This can be attributed to the lack of ligand coverage at the fusion interface and the presence of spatial environments unfavorable for ligand adsorption, such as grooves formed during the process."

Figure S13a was added in the revised supporting information.

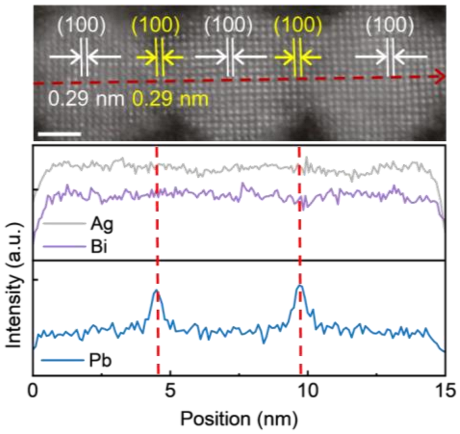

Figure S13. (a) Dark-field scanning TEM image of bridged NCs and EDS line scanning of Ag, Bi, Pb elements. Pb accumulates at the bridging region.

C2.2 Furthermore, why do the authors assume that NC fusion introduces traps (Figure 1f)? While this viewpoint is understandable for traditional PbS and PbSe QDs, where strong quantum confinement causes fusion to significantly alter the bandgap and potentially introduce mid-gap states. AgBiS2 exhibits much weaker quantum confinement, meaning fusion has a negligible effect on its bandgap. Therefore, I suggest the authors de-emphasize these descriptions. The record, certified PCE achieved in this work represents a significant advancement in the field, demonstrating the effectiveness of the proposed surface treatment, even if it is simply a matter of improved surface passivation. Since the description has been revised to a "Molecular Perovskite layer" structure, is the lattice spacing matching still relevant? If the authors maintain that this is critical, please provide HRTEM images for other perovskite compositions after the exchange.

Author Response:

Thank you for the reviewer's suggestion.

Extensive research has demonstrated that fused nanocrystal dimers constitute the primary source of optically active trap states in nanocrystal solids (doi.org/10.1016/j.matt.2019.05.015). Specifically, in the $AgBiS_2$ nanocrystal system, it has been reported that fusion can introduce lattice defects, such as point defects and

dislocations at the interfaces, leading to detrimental effects (doi.org/10.1038/s41566-021-00950-4). As the reviewer noted, $AgBiS_2$ exhibits a weak quantum confinement effect, but we believe that in the $AgBiS_2$ system, the fusion behavior does not introduce defects primarily through influencing the bandgap.

In many studies involving the introduction of perovskite into nanocrystal films, whether as a shell, a bridging layer, or a filling matrix, the importance of lattice matching with the host nanocrystal material has been emphasized (DOI: 10.1021/acs.nanolett.5b03271; doi.org/10.1016/j.joule.2020.05.011; DOI: 10.1021/acsnano.6b04721; doi.org/10.1021/ja5006288). In the present study, lattice matching between the perovskite molecular layer and $AgBiS_2$ nanocrystals remains crucial. It is one of the prerequisites enabling the epitaxial growth of the perovskite layer along the (100) planes of the nanocrystals, thereby bridging them. In fact, during the initial exploratory phase of this work, we attempted ligand exchange by introducing additional lead halide into the control ligand solution (MAI + AgI), with the aim of forming $MAPbI_3$ perovskite (6.31 Å) to bridge the nanocrystals. However, this approach proved to be unsuccessful.

As suggested, we have added an additional Cs-corrected scanning dark-field TEM image as shown in Figure S13a to clearly visualize the atomic-scale morphology of nanocrystal bridging. The corresponding line scan confirms the enrichment of Pb at the bridging region, verifying the effective bridging of nanocrystals by lead halide perovskite molecular layer.

As suggested, we have also supplemented the corresponding HRTEM images (Figure S13g), which show that oriented bridging of the nanocrystals did not occur, further confirming the importance of lattice matching.

The following sentences have been modified in the main text on page 9 lines 5-9: "The HR-TEM images show that the NCs are effectively bridged to one another (Figures 3a and S13a), while the control NCs are significantly fused and aggregated (Figures S13b and S13c). The presence of such dimers has been shown to introduce numerous trap states at the interface, with the consequence that this affects the charge transport."

The following sentences have been added in the main text on pages 10 lines 1-2: "Furthermore, lattice matching is also a prerequisite for bridging NCs (Figure S13g)."

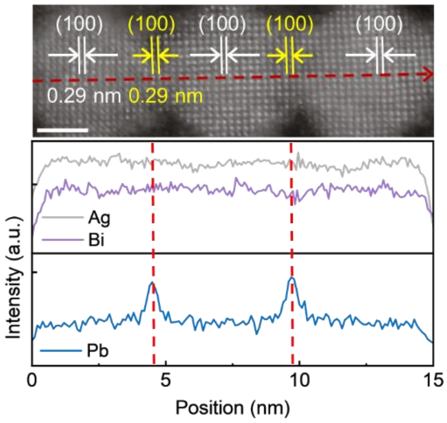

Figure S13. (a) Dark-field scanning TEM image of bridged NCs and EDS line scanning of Ag, Bi, Pb elements. Pb accumulates at the bridging region. Scale bars: 2 nm.

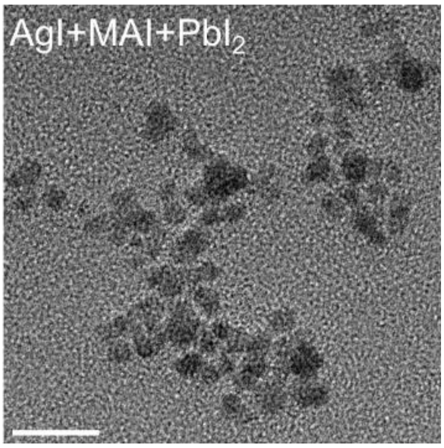

Figure S13. (g) HRTEM image of the NCs subjected to ligand exchange with ($AgI+MAI+PbI_2$). The absence of oriented bridging in the NCs confirms the importance of lattice matching ($MAPbI_3$: 6.31 Å *vs*. $AgBiS_2$: 5.69 Å). Scale bars: 20 nm.

C2.3 Additionally, I believe the authors should explicitly specify "lead halide perovskite" in both the title and the main text.

Author Response:

As suggested, we have modified the description related to perovskites to refer specifically to lead halide perovskites, especially in the title and the main text.

Reviewer #3

General comments: This manuscript reports a method of improving the PCE of silver bismuth sulphide nanocrystal solar cells by adding lead perovskite to the active layer. Aside from the mildly questionable decision to add lead to a lead-free material, the film does appear to enable higher efficiency in the nanocrystalline solar cells, an improvement of between 1-2% depending on which previous/current measurements are to be believed. At this stage, the manuscript has been reviewed at least once and the authors have responded to all the questions, mostly successfully. Although a few minor questions remain, the major impediment to publication has been the use of perovskite as a additive transport material in the functional layer which has to some degree already been implemented previously with nanocrystal (lead sulphide) films. In my opinion, the small increase in efficiency, over previously reported lead-free cells, perhaps is not enough to warrant publication in this journal. It is clear the perovskite helps a little, but the mechanism does not appear very unusual or novel nor is it clear how this will lead to improvements beyond 12% PCE, which are critical to achieving the goal of supplanting other solar technologies (>20 % PCE). The following issues should also be addressed, prior to publication anywhere.

Response:

The comments provided by the reviewer are greatly appreciated.

The concept of embedding perovskite into nanocrystal films was first proposed by Prof. Zhijun Ning as early as 2015 (doi.org/10.1038/nature14563). Building upon this idea, various strategies and methods for perovskite incorporation have been reported over the following decade, significantly advancing the development of nanocrystal solar cells (doi.org/10.1038/s41586-019-1239-7, doi.org/10.1016/j.joule.2020.05.011, DOI: 10.1021/acsnano.6b04721, doi.org/10.1021/ja5006288). The issue of novelty is recognized as a source of concern. However, it should be noted that for different material systems, the underlying motivations, specific implementation schemes, and the resulting outcomes each possess distinct characteristics.

This investigation is directed towards the methodology associated with the inherent properties of $AgBiS_2$, which exhibits unambiguous methodological and mechanistic innovation in comparison to the PbS system (doi.org/10.1016/j.joule.2020.05.011). The present study focuses on the introduction of lattice-matched $CsPbBr_3$ via a ligand-assisted route, where the $CsPbBr_3$ molecular perovskite layer performs two critical, synergistic functions in $AgBiS_2$ nanocrystal films.

Firstly, it has been demonstrated that the material under investigation significantly enhances charge carrier mobility and diffusion length. While perovskite bridging has been demonstrated to deliver marginal photocurrent gains in PbS systems (which have inherently long diffusion lengths and are typically processed as 350-450 nm thick films), the present strategy has a transformative effect on $AgBiS_2$. It overcomes the material's intrinsic short diffusion length, enabling the active layer thickness to increase from ~40 nm to 185 nm. This results in a greatly improved near-infrared external quantum efficiency and a ~7 mA • cm^{-2} boost in photocurrent (Figure 5g).

Secondly, and more fundamentally, this perovskite layer enables precise cation-disorder engineering of $AgBiS_2$, a key innovation not demonstrated in prior work. The optoelectronic performance of $AgBiS_2$ has been shown to correlate positively with cation disorder, a phenomenon promoted by elevated annealing temperatures. It has been also demonstrated that elevated temperatures can lead to deleterious nanocrystal fusion. The excellent thermal stability of the $CsPbBr_3$ layer was exploited in order to suppress fusion along the (100) facets, thus enabling the annealing temperature to be safely raised from 115 ° C to 150 ° C. This fully unlocks the ultrahigh light absorption potential of $AgBiS_2$ without introducing fusion defects (doi.org/10.1038/s41566-021-00950-4), leading to a doubling of carrier mobility, further enhanced light absorption, and an additional ~3 mA • cm^{-2} photocurrent gain (Figure 5d). This pioneering approach of utilizing a perovskite matrix to stabilize and enhance the cation-disorder state has not been previously documented in the study of PbS systems, thereby substantiating the originality of our research in this nascent photovoltaic system.

The innovation of our work lies in designing a perovskite bridged strategy specifically

for $AgBiS_2$. This approach directly addresses the core bottlenecks of the process, namely the short diffusion length and the trade-off between thermal processing and performance optimization. The result is a breakthrough enhancement through a distinct and material-specific mechanism.

This aforementioned approach allows us to fabricate $AgBiS_2$ nanocrystal thick films featuring high light-harvesting capability and high carrier mobility, leading to a drastically enhanced photocurrent and a resulting PCE exceeding 12%. This entire process has been elaborated in detail in the manuscript.

With regard to the reviewer's observation on the modest enhancement in efficiency, we respectfully submit a divergent viewpoint. In the field of $AgBiS_2$ nanocrystal photovoltaics, two seminal studies published in *Nature Photonics* in 2016 and 2022 achieved certified efficiencies of 6.3% and 8.95%, respectively (doi.org/10.1038/nphoton.2016.108; doi.org/10.1038/s41566-021-00950-4). By the end of 2024, this certified efficiency was further advanced to 9.4% (doi.org/10.1039/D4EE03266G). The implementation of the strategy outlined in our work has resulted in the attainment of **a new certified efficiency record of 11.22%,** signifying an approximate enhancement of 20%. We believe this enhancement signifies a substantial progression for this system, which exhibits considerable potential and is in its nascent stages of development.

C3.1 the authors suggest that the conduction is improved by adding the perovskite and bonding the NCs together, however the conduction mechanism is not strongly studied in this work or, to my knowledge, in previous works in adequate detail to discuss the charge transfer mechanism. Authors should cite previous work if possible, otherwise the mechanism (tunneling vs hopping) is vague as are the states involved in transport between nearest neighbors. What they suggest is reasonable, given what is known about perovskite, but this work is primarily an "additive leads to higher PCE" manuscript, not a transport study. The conduction mechanisms of silver bismuth sulphide nanocrystals, including low temperature and trap filling experiments, need to be better understood to make strong generalizations here.

Author response:

We thank the reviewer for the constructive suggestions.

It should be noted that the core focus of this work is not to fundamentally name a new charge transport mechanism in nanocrystal films. Our temperature-dependent mobility results show that the charge transport in the nanocrystal films, with and without perovskite bridging, both follows the classical hopping transport model (Figure S18, manifested as reduced mobility at lower temperatures), as detailed on Page 11 of the manuscript. Our work aims to lower the hopping transport barrier between nanocrystals via perovskite bridging to enhance carrier mobility.

Following the reviewer's suggestion, we have added a citation to relevant studies on charge hopping transport in nanocrystal films and modified the corresponding discussion on page 11 lines 7-10: "Temperature-dependent mobility analysis (Figure S18b) reveals thermally activated "hopping" transport in both bridged NC films and control NC films,[41,42] with carrier mobility decreasing as temperature declines."

A new refence was added in the revised manuscript.

[42] Emin, D. Lattice relaxation and small-polaron hopping motion. *Phys. Rev. B* **1971**, *4*, 3639-3651.

C3.2 Why lead perovskite? Why not Sn, since one imagines the improvement might be similar.

Authors response:

It should be clarified that the $AgBiS_2$ NC films under discussion are fabricated in ambient air. Tin-based perovskites have intrinsically poor moisture and oxygen stability, making them highly prone to degradation in air via phase transition and Sn^{2+}-to-Sn^{4+} oxidation; their introduction will inevitably impair film stability and charge transport performance. By contrast, lead-based perovskites, particularly $CsPbBr_3$, possess

outstanding ambient stability. This is a primary consideration when selecting perovskite materials.

The discussion of the aforementioned innovative points has been strengthened in the revised manuscript, with corresponding modifications made throughout the text to more clearly highlight the scientific contributions of this work. We would like to express our gratitude once again for the valuable time and professional review you have provided.

With Best Regards.
